# Exploring *Monacha* species from the island of Corfu (NW Greece) continues: redescription of *M. claustralis*, *M. parumcincta*, and some allied Italian species (Gastropoda, Eupulmonata, Hygromiidae)

**DOI:** 10.3897/zookeys.1290.183303

**Published:** 2026-08-18

**Authors:** Giuseppe Manganelli, Joanna R. Pieńkowska, Debora Barbato, Andrea Benocci, Katarzyna Sosnowska-Kałasz, Andrzej Lesicki, Folco Giusti

**Affiliations:** 1 Dipartimento di Scienze Fisiche, della Terra e dell'Ambiente, Università di Siena, Via Mattioli 4, 53100 Siena, Italy Department of Cell Biology, Institute of Experimental Biology, Faculty of Biology, Adam Mickiewicz University in Poznan Poznań Poland; 2 NBFC (National Biodiversity Future Center), Piazza Marina 61, 90133 Palermo, Italy NBFC (National Biodiversity Future Center) Palermo Italy; 3 Department of Cell Biology, Institute of Experimental Biology, Faculty of Biology, Adam Mickiewicz University in Poznan, Uniwersytetu Poznańskiego 6, 61-614 Poznań, Poland Dipartimento di Scienze Fisiche, della Terra e dell’Ambiente, Università di Siena Siena Italy; 4 Museo di Storia Naturale dell'Accademia dei Fisiocritici, Piazzetta S. Gigli 2, 53100 Siena, Italy Museo di Storia Naturale dell’Accademia dei Fisiocritici Siena Italy

**Keywords:** Genitalia, integrative approach, mitochondrial and nuclear genes, nomenclature, shell, taxonomy, variation

## Abstract

Four species of the hygromiid genus *Monacha* (*M. claustralis*, *M. cartusiana*, *M. parumcincta*, *M. manfredonica*) are redescribed using an integrative approach including analysis of morphological (shell and genitalia) and molecular features (nucleotide sequences of mitochondrial and nuclear genes) of specimens collected from topotypical localities to resolve their nomenclatural problems. Redescriptions are accompanied by a historical analysis of the names previously associated with these species and study of any available type material. Neotypes are designated for *M. claustralis* and *M. parumcincta*. Some allied species from southern Italy and Sicily, i.e. *M. gregaria*, *M. rizzae*, *M. cf. consona*, and an unnamed species from central and southern Calabria are also described and revised, comparing them with the others on a morphological and molecular basis.

## Introduction

Two species belonging to the highly diverse hygromiid genus *Monacha* Fitzinger, 1833, are reported historically from the island of Corfu (Kérkyra, Ionian Islands, NW Greece) under various names (Mousson 1859a; Hesse 1882; Paganetti-Hummler 1905). These species are currently recognised as *Monacha claustralis* (Rossmässler, 1834) and *Monacha parumcincta* (Rossmässler, 1834) but have complex taxonomic and nomenclatural histories with some unresolved aspects.

To address their taxonomy and nomenclature, we previously investigated new populations from their type locality (Corfu/Kérkyra) with an integrative approach which included morphological (shell and genitalia) and molecular data (mitochondrial and nuclear gene sequences) (Manganelli et al. 2025). The aims of the research were to address the relationships between topotypical *Monacha claustralis* and the presumably related *Monacha cartusiana* (Müller, 1774) and between topotypical *Monacha parumcincta* and the Italian populations so far assigned to this species. The results proved that shell features did not differentiate the pairs analysed, i.e. *Monacha claustralis* vs *M. cartusiana* and Corfiot vs Italian *M. parumcincta*, whereas features of distal genitalia structure and nucleotide sequences of mitochondrial genes distinguished them significantly. Nuclear gene features also differentiated between Corfiot and Italian *M. parumcincta*. We therefore postulated (Manganelli et al. 2025) that these two pairs were composed of four distinct species: *M. claustralis*, *M. cartusiana*, Corfiot *M. parumcincta*, and Italian *M. parumcincta*.

Now we conclude the work by redescribing these four and other related species, mainly from southern Italy and Sicily, with new morphological and molecular data, and a detailed study of nomenclatural issues and type materials.

## Material and methods

### Taxonomic sample

Beyond the taxa already investigated with integrative approach based on morphological (shell and genitalia) and molecular analyses by Manganelli et al. (2025), this paper also considers others, such as *Monacha gregaria* (Rossmässler, 1839) and *Monacha rizzae* (Aradas, 1843) in order to discuss the relationships of the Italian populations so far assigned to *Monacha parumcincta*. Many of the nominal taxa introduced or proposed in the early literature on this group of *Monacha* were also considered and revised.

New sequences of some genes of *Monacha cf. rizzae* from Basilicata (southern Italy) and *Monacha* sp. 3 from Calabria (southern Italy) were deposited in GenBank (Table 1). Sequences deposited in GenBank for *M. claustralis*, *M. parumcincta*, *M. cartusiana*, *M. cantiana* (Montagu, 1803) s.l., and *M. pantanellii* (De Stefani, 1879), as well as for Sicilian *Monacha* sp. 1 and *Monacha* sp. 2 during previous studies (Neiber and Hausdorf 2017; Pieńkowska et al. 2018a, 2018b, 2019, 2020, 2022, 2024; Manganelli et al. 2025) were also used in molecular analysis. Sequences of *Trochulus hispidus* (Linnaeus, 1758) from GenBank (Neiber and Hausdorf 2017) were used as an outgroup to construct phylogenetic trees.

**Table 1.** GenBank sequences used in molecular analysis.

**COI**	**16S rDNA**	**5.8S rDNA + ITS2 + 28S rDNA**	**References**
***Monacha cartusiana***			
KX507189, KX507235,	KX495378, KX495429	KX495431, KX495479	Neiber and Hausdorf 2017 (KX)
ON332653, ON332655	ON350961, ON350963, ON350964	ON332790	Pieńkowska et al. 2022 (ON)
PP947906, PP947907, PP947910, PP947912, PP947913, PP947914, PP947919, PP947920, PP947923	PP949426, PP949429, PP949431, PP949432, PP949433, PP949438, PP949439, PP949442	PP947989, PP947990, PP947991	Manganelli et al. 2025 (PP)
***Monacha claustralis***			
KX507199	KX495388	KX495441	Neiber and Hausdorf 2017 (KX)
PP947873, PP947874, PP947875, PP947876, PP947879, PP947880, PP947881, PP947882, PP947883, PP947884, PP947886, PP947887	PP949387, PP949388, PP949389, PP949390, PP949393, PP949394, PP949395, PP949396, PP949397, PP949398, PP949400, PP949401	PP947951, PP947952, PP947953, PP947954, PP947955	Manganelli et al. 2025 (PP)
PX460009	PX621078	PX460016,	This paper (PX)
PZ535806 (neotype)	PZ539312 (neotype)	PZ539314 (neotype)	This paper (PZ)
***Monacha manfredonica***			
MG208944, MG208947, MG208949, MG208950, MG208956, MG208959			Pieńkowska et al. 2018b (MG)
	PP949420, PP949421, PP949422, PP949423, PP949424, PP949425	PP947984, PP947985, PP947986	Manganelli et al. 2025 (PP)
***Monacha pantanellii***			
MT380014, MT380015, MT380045, MT380048, MT380061, MT380062			Pieńkowska et al. 2020 (MT)
	PP949446	PP948011	Manganelli et al. 2025 (PP)
	PV470045, PV470046, PV470047, PV470048, PV470049	PV463530, PV463531, PV463532, PV463533, PV463534	Pieńkowska et al. 2025 (PV)
***Monacha parumcincta***			
PP947890, PP947891, PP947892, PP947895, PP947896, PP947897, PP947898, PP947899, PP947900, PP947901, PP947902, PP947903	PP949404, PP949405, PP949406, PP949409, PP949410, PP949411, PP949412, PP949413, PP949414, PP949415, PP949416, PP949417	PP947968, PP947969, PP947973	Manganelli et al. 2025 (PP)
PZ535807 (neotype)	PZ539313 (neotype)	PZ539315 (neotype)	This paper (PZ)
***Monacha cantiana* s.s. CAN-1**			
MG208910, MG208905			Pieńkowska et al. 2018b (MG)
	OR918428, OR918429	OR917402, OR917403	Pieńkowska et al. 2024 (OR)
***Monacha cantiana* s.s. CAN-2**			
MG208925, MG208928			Pieńkowska et al. 2018b (MG)
	OR918435, OR918436	OR917406, OR917407	Pieńkowska et al. 2024 (OR)
***Monacha cantiana* s.l. CAN-3**			
MG208936, MG208938			Pieńkowska et al. 2018b (MG)
	OR918437	OR917408	Pieńkowska et al. 2024 (OR)
	PV470025	PV463510, PV463511, PV463512	Pieńkowska et al. 2025 (PV)
***Monacha cantiana* s.l. CAN-4**			
MG208939, MG208940			Pieńkowska et al. 2018b (MG)
	OR918438, OR918439	OR9174409, OR917410	Pieńkowska et al. 2024 (OR)
***Monacha cantiana* s.l. CAN-5**			
MK066938, MK066939			Pieńkowska et al. 2019 (MK)
	PP949443	PP948008	Manganelli et al. 2025 (PP)
	PV470028	PV463513, PV463514, PV463515, PV463516, PV463517	Pieńkowska et al. 2025 (PV)
***Monacha cantiana* s.l. CAN-6**			
MK066943, MK066944			Pieńkowska et al. 2019 (MK)
	PP949444, PP949445	PP948009, PP948010	Manganelli et al. 2025 (PP)
**Sicilian *Monacha* sp. 1**			
KX507230, KX507231	KX495424, KX495425	KX495474, KX495475	Neiber and Hausdorf 2017 (KX)
**Sicilian *Monacha* sp. 2**			
KX507229	KX495423	KX495473	Neiber and Hausdorf 2017 (KX)
Italian Monacha cf. rizzae*			
population 1			
PQ566664, PQ566665, PQ566666, PQ566667, PQ566668, PQ566669	PV659011, PV659012	PV659024, PV659025, PV659026	This paper (PQ, PV)
population 2			
PQ566670, PQ566671, PQ566672, PQ566673, PQ566674	PV659013, PV659014, PV659015, PV659016, PV659017	PV659027, PV659028, PV659029, PV659030, PV659031	This paper (PQ, PV)
Fiume Alli *Monacha* sp. 3**			
PX651375, PX651376			This paper (PX)
***Trochulus hispidus***			
KX507209	KX495398	KX495451	Neiber and Hausdorf 2017 (KX)

* two populations examined: 1) Strada statale di Sapri, at km 18; 2) Strada statale Fondo Valle del Noce, at km 23. For other sampling details, see Material examined of *M. rizzae*. ** one population examined: Fiume Alli, downstream confluence with Fosso Sarbato. For other sampling details, see Material examined of *Monacha* sp. 3.

### Material examined

Data on the material examined with integrative approach based on morphological (shell and genitalia) and molecular analysis were presented in tables 1 of previous papers (Pieńkowska et al. 2018a, 2018b, 2019, 2020, 2022, 2024; Manganelli et al. 2025). In these tables there is material examined listed as follows for each species: number of shells and specimens; locality; georeference in latitude and longitude when exact collection site is known and in MGRS UTM 10 × 10 km when it is only approximately known; collectors and date; location of specimens and inventory number. Additional new material, studied only morphologically, is reported under the redescriptions of the taxa below.

Acronyms of institutions containing voucher specimens: **AIC** A. Irikov coll. (University of Plovdiv, Bulgaria), **DCBC** Department of Cell Biology coll. (Adam Mickiewicz University, Poznań, Poland), **FGC** F. Giusti coll. (University of Siena, Italy), **NHMD** Natural History Museum of Denmark (Copenhagen, Denmark), **SMF** Senckenberg Forschungsinstitut (Frankfurt am Main, Germany), **ZMH** Zoological Museum of the Leibniz Institute for the Analysis of Biodiversity Change, Hamburg (Germany).

### Morphological study

Snail bodies were dissected under a light microscope (Wild M5A or Zeiss SteREO Lumar V12). Anatomical details were drawn using a Wild camera lucida. The anatomical nomenclature and descriptive and directional terms are used as explained by Manganelli et al. (2023, with references). Shell and genital sections measured as reported by Pieńkowska et al. (2018b: fig. 1). Whorl number counted from the apex of the spire (Kerney and Cameron 1979: fig. on p. 13). Abbreviations/Acronyms are as follows (see also Pieńkowska et al. 2018b: figs 1, 2): **AH** aperture height, **AW** aperture width, **BC** bursa copulatrix (gametolytic gland), **BW** body wall, **DBC** duct of bursa copulatrix, **DG** digitiform glands, **DV** distal vagina (from digitiform glands to genital atrium), **E** epiphallus (from base of flagellum to beginning of penial sheath), **F** flagellum, **FO** free oviduct, **GA** genital atrium, **NW** number of whorls, **P** penis, **PP** penial papilla, **PSO** prostatic section of spermoviduct, **SD** shell diameter, **SH** shell height, **SOD** spermoviduct, **UD** umbilicus diameter, **USO** uterine section of spermoviduct, **V** vagina, **VA** vaginal appendix (appendicula), **VD** vas deferens, **VR** vaginal refringent ring, **VS** vaginal sac.

### Molecular study

The molecular methods are described in the previous papers (Pieńkowska et al. 2015, 2018b, 2022; Manganelli et al. 2025) except for use of MEGA12 (Kumar et al. 2024) instead of MEGA7 program to determine phylogenetic relationships together with IQ-Tree (Nguyen et al. 2015), and MrBayes v. 3.2.6 (BI) (Ronquist et al. 2012). For phylogenetic analysis, best substitution models were inferred by the Bayesian Information Criterion (BIC) for the various partitions using ModelFinder (Kalyaanamoorthy et al. 2017). Phylogenetic analysis by IQ-Tree was performed for two sets of concatenated sequences, dividing particular data sets into two or three partitions (Chernomor et al. 2016). The set COI+16SrDNA was divided into two partitions, COI and 16SrDNA with best substitution models: HKY+F+I+G4 for partition 1, and GTR+F+I+G4 for partition 2. The set of ITS2 with flanking sequences of 5.8S and 28SrDNA gene fragments was divided into three partitions, 5.8SrDNA, ITS2, and 28SrDNA with best substitution models: F81+F for partition 1, K2P+G4 for partition 2, and F81 for partition 3. Bayesian analyses were performed with the same partitions. Bayesian analysis was conducted with four Monte Carlo Markov chains running for 1 million generations, sampling every 100 generations (the first 25% of trees were discarded as ‘burn-in’) (Manganelli et al. 2025). The robustness of the ML trees generated by MEGA12 was assessed by bootstrap analysis with 1000 replicates (Felsenstein 1985). ML trees obtained with IQ-Tree were constructed under SH-aLRT and 1000 ultrafast bootstrap replicates (Hoang et al. 2018). Finally, BI trees were supported by posterior probability (pp) values. Bootstrap support values from ML analysis as well as pp values obtained on a 50% majority-rule consensus Bayesian tree were mapped onto the ML tree obtained by IQ-Tree.

## Results

### Redescriptions of *M. claustralis*, *M. parumcincta*, and allied species

***Monacha claustralis* (Rossmässler, 1834)**

**Figure 1.**
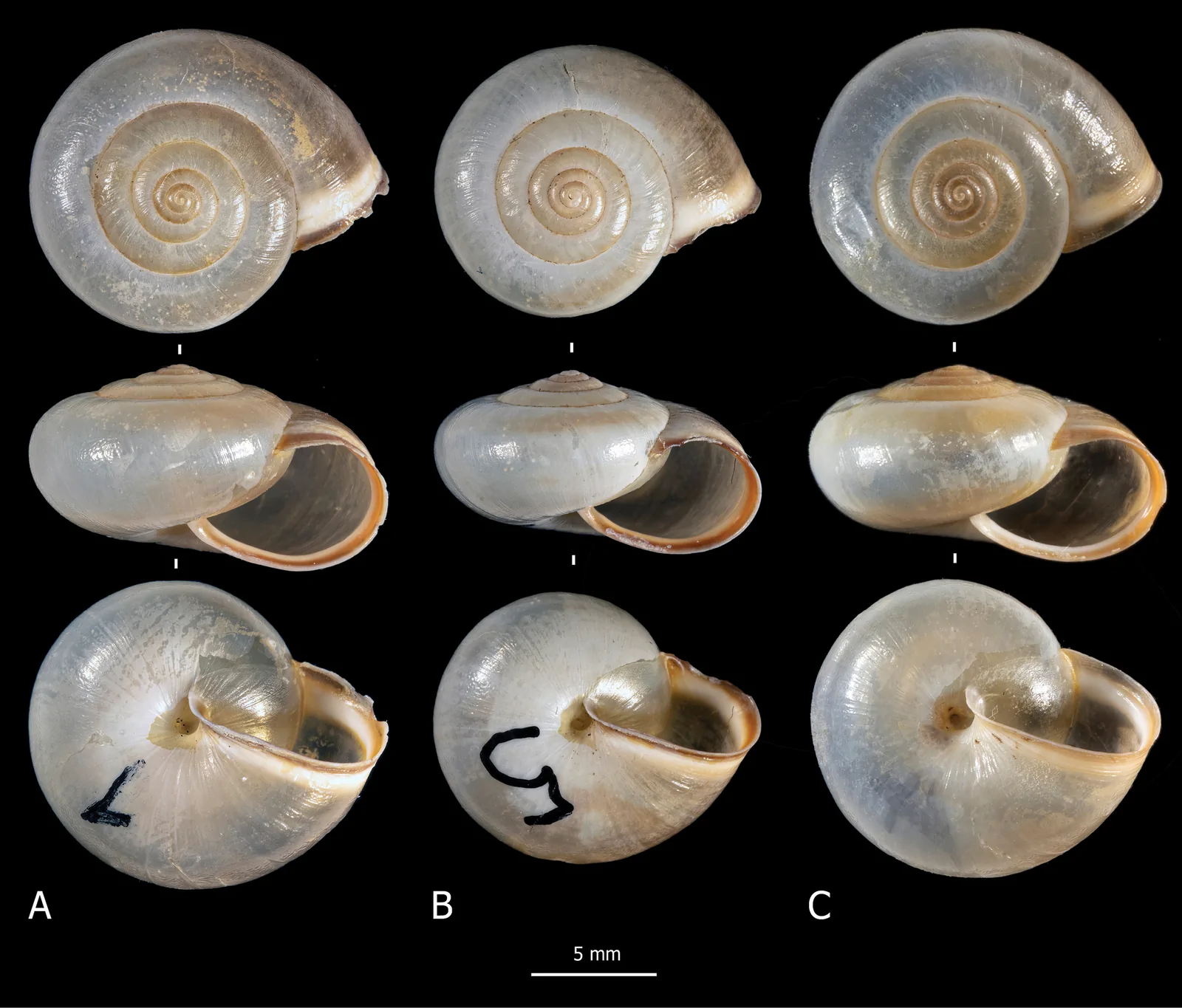
Shells of *Monacha claustralis* from Corfu. Specimens from Molos cemetery, A. Benocci, G. Manganelli & L. Manganelli leg. 26 Oct. 2022 (FGC 52301) (**A, B**) and (SMF 383165, neotype) (**C**).

**Figure 2.**
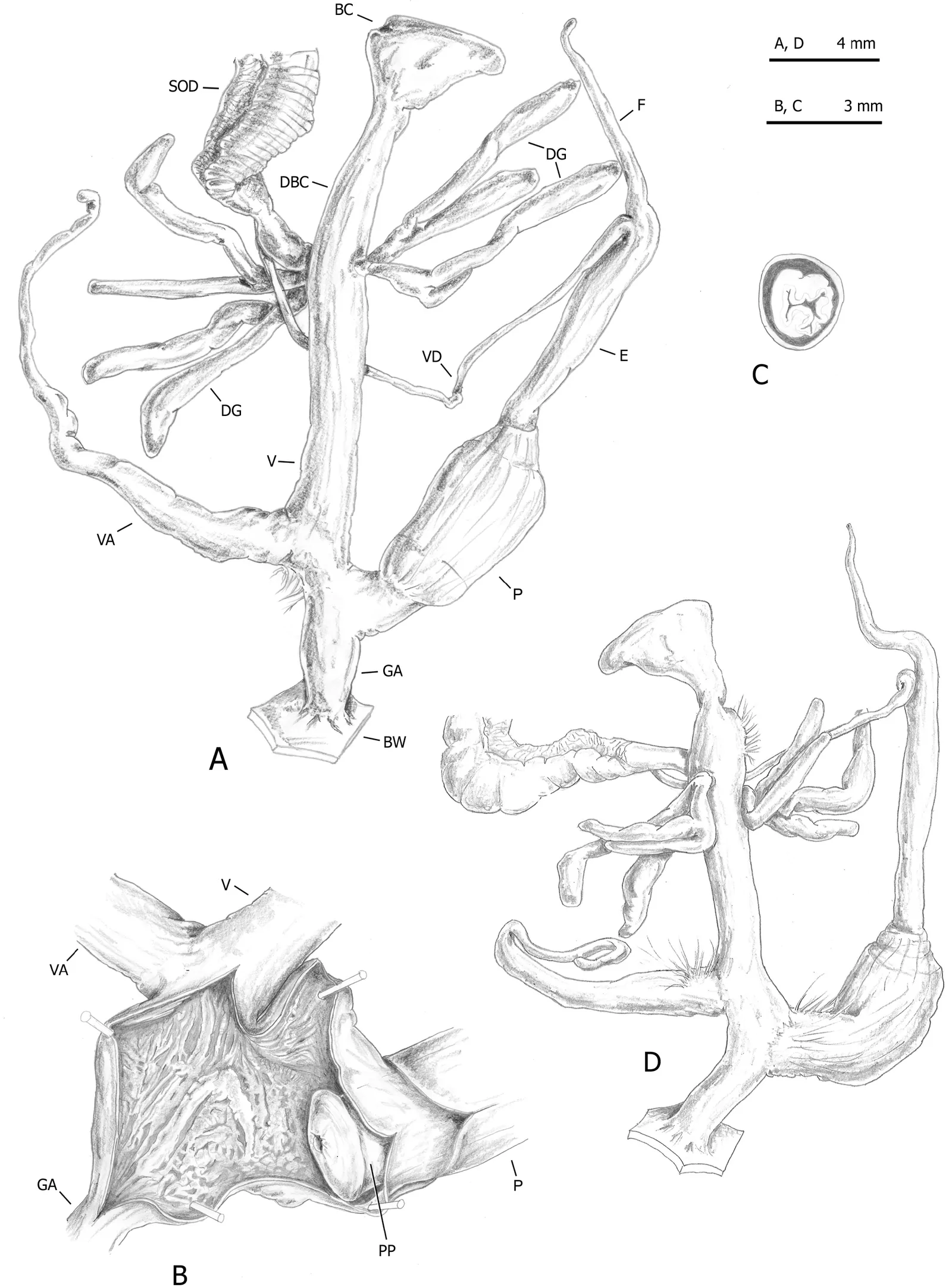
Genital anatomy of *Monacha claustralis*. Specimens from Molos cemetery, A. Benocci, G. Manganelli & L. Manganelli leg. 26 Oct. 2022 (FGC 52301) (**A–C**) and (SMF 383165, neotype) (**D**). Distal genitalia (**A, D**), internal structure of vagina and penis (**B**) and transverse section of apical penial papilla (**C**).

Figs 1, 2, 20, 21, 27

*Helix claustralis* Rossmässler, 1834: 5, as variety of *Helix carthusianella*. Type locality: Corfu, Greece. Type material: unknown and probably lost (see below and Remarks). Neotype selected SMF 383165 (Figs 1C, 2D).

**Justification for neotype designation (ICZN Art. 75.3)**

75.3.1. Purpose. A neotype for *Monacha claustralis* (Rossmässler, 1834) is here designated with the purpose of stabilising and clarifying the taxonomic status and type locality of this nominal species-group taxon.

75.3.2. Differential diagnosis. *Monacha claustralis* is distinguished from the other species of the genus by the characters listed/discussed in the Remarks (below).

75.3.3. Data ensuring recognition of the neotype. The shell is illustrated in Fig. 1C (apical, apertural and umbilical views); the distal genitalia are illustrated in Fig. 2D. Dimensions of the shell and distal genitalia are summarised in Table 2. Mitochondrial and nuclear sequence data are deposited in GenBank under accession numbers (Table 1). The following additional features distinguish the neotype: shell with a small dent on upper surface of last whorl, ~ 1/2 whorl from aperture, and small sub-peripheral chip in external margin of peristome; body devoid of hind part of foot which was used for molecular analysis; distal genitalia with very short duct of bursa copulatrix, featuring bottleneck before the bursa copulatrix.

**Table 2.** Shell and genitalia dimensions (in mm) of the neotype of *Monacha claustralis* (SMF 383165).

**Shell**						**Genitalia**					
SH	SD	AH	AW	UD	NW	DBC	V	VA	F	E	P
7.9	14.4	5.0	7.9	0.9	5 ^3^/_4_	2.4	6.0	12.2	6.2	6.6	6.2

75.3.4. Evidence that the original name-bearing type is lost, and steps taken to trace it. Rossmässler’s collection is at the Senckenberg Forschungsinstitut (Frankfurt am Main) (Zilch 1967). In 2024, we contacted Sigrid Hof of the Section Malacology of SMF for specimens of *Helix claustralis* or Helix carthusianella var. claustralis from Corfu in Rossmässler’s collection. Since no specimen was found (Sigrid Hof pers. comm. 15 July 2024) we can reasonably conclude that the original name-bearing type is lost.

75.3.5. Consistency with the former name-bearing type. The neotype is consistent with the description of the former name-bearing type characterised by a shell 8" in width and 5" in height, with the last whorl uniformly rounded (see Rossmässler’s description: “*Claustralis* aus Corfu (8" breit und 5" hoch) unterscheidet sich nur durch die Grösse und durch den völlig gleich gerundetetn letzten Umgang, da man bei der Wiener *carthusianella* auf der Höhe des letzten Umgangs eine Spur eines ganz abgerundeten Kiels wahrnimmt, unterhalb dessen sich der Umgang merklich einzieht”). A lectotype was designated by Hausdorf (2000) for another concept of the species, namely that based on the assumption that the taxon name had been made available since Menke (1828) under Art. 11.6.1 by Mousson (1859a). However, we dispute this interpretation, since Menke (1828) reported it as a synonym of variant “a”, for which no name was used as valid (ICZN 1999, Art. 11.6). Even if it were accepted that Menke’s (1828) synonym was made available later under Art. 11.6.1, Mousson’s specimens were not syntypes, because the name-bearing type would have to be taken from Rossmässler (1834) who was the first to use this name validly for a taxon (ICZN 1999, Art. 72.4.3). Authorship controversies aside, the concept of the species does not change with respect to Hausdorf (2000). The lectotype designated by Hausdorf (2000) is not known in anatomical or molecular terms, although it came from an area where populations studied anatomically occur. The neotype designated here is better defined, since it is characterised both morphologically and in molecular terms. A neotype molecularly characterised is very important for the further development of research on this species, whose non-native populations appear to hybridise with those of *Monacha cartusiana* in some areas (see Remarks below).

75.3.6. Provenance relative to the type locality. The species was disseminated as *Helix claustralis* by the shell dealer Franz A. Ziegler. Rossmässler (1834) was responsible for the first valid use of Ziegler’s handwritten name for a taxon from Corfu, based on materials disseminated by Ziegler. The species is widespread on Corfu, especially in the central-southern sector; the choice fell on this location due to the availability of material.

75.3.7. Deposition. The neotype was deposited in the malacological collection of the Senckenberg Forschungsinstitut (Frankfurt am Main, Germany), to be kept with what remains of Rossmässler’s collection. The catalogue number is SMF 383165.

**Neotype**. SMF 383165 (Figs 1C, 2D), a specimen collected near Molos cemetery, Corfu [Kérkyra], Greece (39°26'14.67"N, 20°03'19.77"E), by A. Benocci, G. Manganelli and L. Manganelli on 26 October 2022. The body of the neotype was extracted from the shell and dissected to isolate the distal genitalia; the hind part of the foot was used for molecular analysis. Shell and body are preserved separately. The body and distal genitalia are in two distinct 0.5 ml plastic centrifuge tubes with snap caps.

**Topotypes**. FGC 52301 (Figs 1A, 1B, 2A–C), 8 specimens consisting of three shells and five spirit specimens collected near Molos cemetery, Corfu [Kérkyra], Greece (39°26'14.67"N, 20°03'19.77"E), by A. Benocci, G. Manganelli and L. Manganelli on 26 October 2022.

**Other material examined**. Bulgaria • 4 spirit specimens; Plovdiv; 42°08'35.72"N, 24°44'59.51"E; 19 Sept. 2006; A. Irikov leg.; AIC; Montenegro • 8 spirit specimens; Petrovac near Budva, road to Lake Skadar; 42°13'00.3"N, 18°58'43.8"E; 685 m asl; 24 Aug. 2014; J. Pieńkowska leg.; DCBC; Greece • 28 shells, 5 spirit specimens; Corfu [Kérkyra], Gardiki; 39°28'36.32"N, 19°53'06.07"E; 40 m asl; 26 Oct. 2022; A. Benocci, G. Manganelli & L. Manganelli leg.; FGC 52297 • 9 spirit specimens; Corfu [Kérkyra], Benitses; 39°32'29.98"N, 19°54'50.49"E; 5–10 m asl; 24 Oct. 2022; A. Benocci, G. Manganelli & L. Manganelli leg.; FGC 52237; Poland • 54 shells, 30 spirit specimens; Poznań-Morasko; 52°27'57.35"N, 16°55'22.60"E; 05 Oct. 2011; J. Pieńkowska leg.; DCBC and FGC 60107 • 6 spirit specimens; same locality; 15 Sept. 2020; A. Lesicki leg.; DCBC.

**Morphological description**. Shell (1A–C; Manganelli et al. 2025: fig. 2) dextral, small to medium in size, subdiscoidal to subglobose, rather fragile, sub-transparent, milky to horn-yellow in colour, with white and reddish collabral band near aperture. Whorls 5–6, slightly convex and separated by superficial sutures; last whorl ~ 9/10 of shell height, slightly dilated and descending slightly near aperture. Aperture rather large, oval to elliptical (AW/AH ~ 1.7) and slightly prosocline. Peristome interrupted, slightly reflected on columella, thickened and with variably evident whitish callous rim on internal outer margin. Umbilicus open, very small to small. Protoconch and teleoconch smooth, with very faint scattered collabral growth lines. Shell dimensions: H – 7.4 ± 0.9 mm; D – 12.5 ± 1.8 mm (*n* = 12).

Female distal genitalia (Fig. 2A, B, D; Manganelli et al. 2025: figs 8–10) include free oviduct, bursa copulatrix and its duct, digitiform glands, vagina and vaginal appendix. Free oviduct short and variably wide. Bursa copulatrix rather large, triangular, shoe- or bean-like, with short to rather long duct (usually as long as vagina). Vagina rather long to long, without refringent ring or lateral sac between digitiform glands and vaginal appendix, and with series of narrow irregular internal pleats, one of which is sometimes larger. Digitiform glands disposed on opposite sides of vagina in two groups of one or two tufts, each with one or two branches. Vaginal appendix usually rather long (~ 1.5× length of vagina), inserted near distal end of vagina and with dilated basal portion (1/3 of total length) and slender digit-like distal portion (2/3 of total length).

Male distal genitalia (Fig. 2A–D; Manganelli et al. 2025: figs 8–10) include vas deferens, flagellum, epiphallus, and penis. Vas deferens very long and very slender. Flagellum short, slender, and pointed at tip. Epiphallus rather long and wide. Penis short and wide, enveloped by thin sheath. Penial papilla cylindrical, dilated at tip, with apical opening, usually thin external walls and internal lumen occupied by variably slender central duct, round in section.

Genital atrium (Fig. 2A, B, D; Manganelli et al. 2025: figs 8–10) rather short and wide, internally smooth or with variably developed, faintly defined pleats.

**Molecular description**. Neotype sequences (COI – PZ535806, 16SrDNA – PZ539312, ITS2 with flanking fragments – PZ539314) and other molecular sequences obtained from specimens from the type locality of *M. claustralis* are treated as typical of this species: COI – PP947873–PP947889, PX460009, 16SrDNA – PP949387–PP949403, PX621078, ITS2 (with flanking fragments of 5.8SrDNA and 28SrDNA) – PP947951–PP947967, PX460016 (see Table 1).

**Remarks**. The nomenclatural status of this nominal species has been broadly discussed by Welter-Schultes (2012a), whose conclusions are substantially adopted here.

Menke (1828: 12, 1830: 21) introduced *Helix claustralis* as synonym of a subordinate taxon (variant “a”) of *Helix carthusianella* Draparnaud, 1801, without any other information, not even with a locality, attributing it to the shell dealer Franz A. Ziegler.

Some years later, Rossmässler (1834: 5) mentioned *Helix claustralis* from Corfu and *Helix lucernalis* from Dalmatia, stating that both should probably be considered as varieties of *Helix carthusianella*, giving a short diagnosis for each of them and again assigning them to Ziegler without any explicit reference to Menke (1828, 1830), even if Rossmässler was evidently aware of Menke’s contributions. Although these names were proposed conditionally, this having occurred before 1961, they are regarded to have been used as valid (Art. 11.5.1) (Welter-Schultes 2012b).

With clear reference to his contribution of 1834 (“Rossm., Diagnos. II. No. 25"), Rossmässler (1837: 37–38, pl. 27, fig. 366[a]) first listed *Helix claustralis* and *Helix lucernalis* as synonyms of *Helix carthusianella* and then stated that *Helix claustralis* from Italy was one of its varieties.

*Helix claustralis* Zgl. was subsequently listed in the synonymy of *Helix carthusianella* by Pfeiffer (1841: 55) or in that of *Helix cartusiana* by Pfeiffer (1842: 71, 1848a: 124, 1848b: 132) without any reference to Menke (1828, 1830) or Rossmässler (1834, 1837).

Finally, commenting on *Helix cartusiana*, Mousson (1859a: 254) stated that the variety called var. *claustralis* by M. Parreiss (i.e. Ludwig Parreyss) occurred in Preveza. He gave a brief description of this variety, omitting any reference to previous uses of this name.

In his review of the Turkish *Monacha* species, Hausdorf (2000) stated that the name *Helix claustralis* “Ziegler” was first published by Menke (1828) in synonymy of *Helix carthusianella* and subsequently mentioned by several authors, however Mousson (1859a) was the first to use the name as valid for a taxon, thus making the name available. Hausdorf (2000: 82, pl. 4, fig. 19) selected a lectotype for *Helix claustralis* Menke, 1828 as made available by Mousson (1859a) according to ICZN (1999, Art. 72.4.3), from the specimens from Preveza studied by Mousson (Zoologisches Museum Zürich, 505571a). We agree with Welter-Schultes (2012a), who disagreed with Hausdorf’s (2000) conclusions about Menke’s (1828) authorship of *Monacha claustralis* and his selection of lectotype. Moreover, even if it were accepted that Menke’s (1828) synonym was made available later under Art. 11.6.1, Mousson’s specimens were not syntypes, because the name-bearing type would have to be taken from Rossmässler (1834) who was the first to have used this name as valid for a taxon (ICZN 1999, Art. 72.4.3).

The authorship of *Helix claustralis* must be attributed to Rossmässler (1834) contrary to Hausdorf’s claim (2000). Indeed the name cannot be assigned to Menke (1828, 1830) because he reported it as a synonym of variant “a”, for which no name was used as valid (ICZN 1999, Art. 11.6).

Conversely, Rossmässler (1834) is responsible for the first valid use of this name for a taxon from Corfu based on materials disseminated by Ziegler.

In what remains of Rossmässler’s collection at Senckenberg Forschungsinstitut (Frankfurt am Main), there are no specimens of *Helix claustralis* or Helix carthusianella var. claustralis from Corfu (Sigrid Hof pers. comm. 15 July 2024). As for the shell depicted in fig. 366[a] by Rossmässler (1837), if it really is from Italy, it cannot belong to *Monacha claustralis*, because this species is absent in Italy, but must belong to *Monacha cartusiana*. Since this is a subsequent reference, it does not affect the type material (or the type locality) of *Helix claustralis*, which consists of the specimens reported when it was first established (i.e. Rossmässler 1834).

Since the name *Monacha claustralis* is widely used, e.g. in MolluscaBase eds (2025) and the original material which was the basis for the description of the species by Rossmässler (1834) is lost, we believe that redescription of this species should be supplemented by designation of a neotype from the specimens we collected in Corfu. In order to definitively clarify the identity of Rossmässler’s variety, a neotype was selected from specimens collected at Molos (A. Benocci, G. Manganelli and L. Manganelli leg. 26 October 2022). Data ensuring recognition of the neotype are given above. The neotype is kept in the malacological collection of SMF with the registration number 383165.

The species was first studied anatomically and differentiated with respect to *Monacha cartusiana* by Pintér (1968), who reported it as a new species: *Monacha dissimulans* Pintér, 1968. In his exhaustive revision of the Turkish *Monacha* species, Hausdorf (2000) proposed a detailed redescription based on Greek and Turkish populations. He confirmed that it can only be distinguished from *Monacha cartusiana* on the basis of some anatomical features: absence of lateral vaginal sac, smaller epiphallus/penis, and epiphallus/vagina ratios. Pieńkowska et al. (2015) performed a detailed comparative morphological and molecular analysis (using nucleotide sequence polymorphism in selected mitochondrial and nuclear genes) on ~ 80 specimens of the two species from 16 sites in Poland, Czech Republic, Hungary, Bulgaria, Italy, and Georgia. They found that the two species differed in a series of very evident characters: distal vagina (longer in *M. claustralis* vs shorter in *M. cartusiana*), lateral vaginal sac (absent in *M. claustralis* vs present in *M. cartusiana*), vaginal appendix (inserted near distal end of vagina in *M. claustralis* vs inserted approximately half way along vagina in *M. cartusiana*), flagellum (longer in *M. claustralis* vs shorter in *M. cartusiana*) and genital atrium (internally with sort of wide, spongy, slightly raised pleat separating penis from vaginal appendix in *M. claustralis* vs rather smooth or with thin pleats in *M. cartusiana*). Three of the six characters differed significantly between the two species (F, P, DV) and one (DV) showed values that did not overlap (Pieńkowska et al. 2015: fig. 24, Table 1). Recent re-examination of the two species by Manganelli et al. (2025) confirms that morphological distinction is possibly only based on qualitative and quantitative differences in the genital tracts. The qualitative differences concern the distal vagina, lateral vaginal sac, and vaginal appendix, as already shown by Pieńkowska et al. (2015). The quantitative differences involve two ratios linked to female genitalia (DV/VA and DBC/DV), the most effective parameters for taxonomic discrimination.

The distinctiveness of *M. claustralis* from other species of the genus *Monacha* was confirmed by our analysis of the nucleotide sequences of selected genes (Manganelli et al. 2025: figs 19–22). This was particularly evident in the case of the analysis of mitochondrial genes (COI and 16SrDNA). In the case of nuclear genes (ITS2 with fragments of the flanking genes 5.8S and 28SrDNA), the sequences obtained from Corfu specimens of *M. claustralis* grouped in a separate clade from the sequences of *M. parumcincta* from Italy and Corfu and from the sequences obtained from specimens representing the lineages of *M. cantiana* s.l. studied. However, the sequences of these nuclear genes do not distinguish *M. claustralis* from *M. cartusiana*. Moreover, one specimen (Ben1) found on Corfu had an ITS2 sequence identical to *M. cartusiana* specimens from Quattrovie (Que1 – Que5, see Manganelli et al. 2025). Similar situations with identical ITS2 sequences in specimens identified anatomically and by mitochondrial sequences as *M. claustralis* and *M. cartusiana* were noted in Prague (Pieńkowska et al. 2015, 2016), Moldova and Romania (Lesicki et al. 2024).

Specimens with similar anatomical or molecular features have been reported from Bosnia-Herzegovina, Montenegro, Greece, Germany, Czech Republic, Poland, Bulgaria, Turkey, Ukraine, and Georgia (Pintér 1968, as *Monacha dissimulans*; Schileyko 1978 as *Monacha cartusiana*; Hausdorf 2000; Irikov 2008; Pieńkowska et al. 2015, 2018a; Neiber and Hausdorf 2017; Hutchinson et al. 2019; Čejka et al. 2020; Gural-Sverlova and Gural 2022, 2023).

However, specimens of uncertain or ambiguous attribution have been reported in non-native populations of *Monacha claustralis* and *Monacha cartusiana* across central and eastern Europe (Čejka et al. 2020; Gural-Sverlova and Gural 2022, 2023; Lesicki et al. 2024; Williams et al. 2024). Čejka et al. (2020) described the presence of a vaginal sac in presumed *M. claustralis* specimens from the Czech Republic, suggesting that *M. claustralis* and *M. cartusiana* in central Europe may be distinct lineages of the same species, originating from different regions of its native range. Subsequent studies have further investigated anatomical and genetic variations in these populations. Gural-Sverlova and Gural (2022, 2023) and Williams et al. (2024) reported overlapping genital ratios and variations in the vaginal sac, raising the possibility of hybridisation between the two species. Lesicki et al. (2024) identified specimens with discrepancies between their anatomical features and molecular data: some exhibited the reproductive structures of *M. cartusiana* but carried *M. claustralis* mtDNA, while others, classified anatomically as *M. claustralis*, displayed *M. cartusiana* haplotypes. Additionally, certain specimens with haplotypes of either species showed an unusual female reproductive system, characterised by a moderately long vagina with relatively indistinct vaginal sac. These findings suggest that hybridisation can be occurring, where the mitochondrial genome, inherited maternally, would retain the characters of one species, while the nuclear genome and anatomical traits would show intermediate features to varying degrees (Lesicki et al. 2024). The issue of hybridisation of *M. claustralis* and *M. cartusiana* was not settled and will be the focus of future research.

#### Monacha cartusiana

Taxon classificationAnimaliaStylommatophoraHygromiidae

(Müller, 1774)

5AB55158-08FF-5B90-A2B3-13A19A51BABD

**Figure 3.**
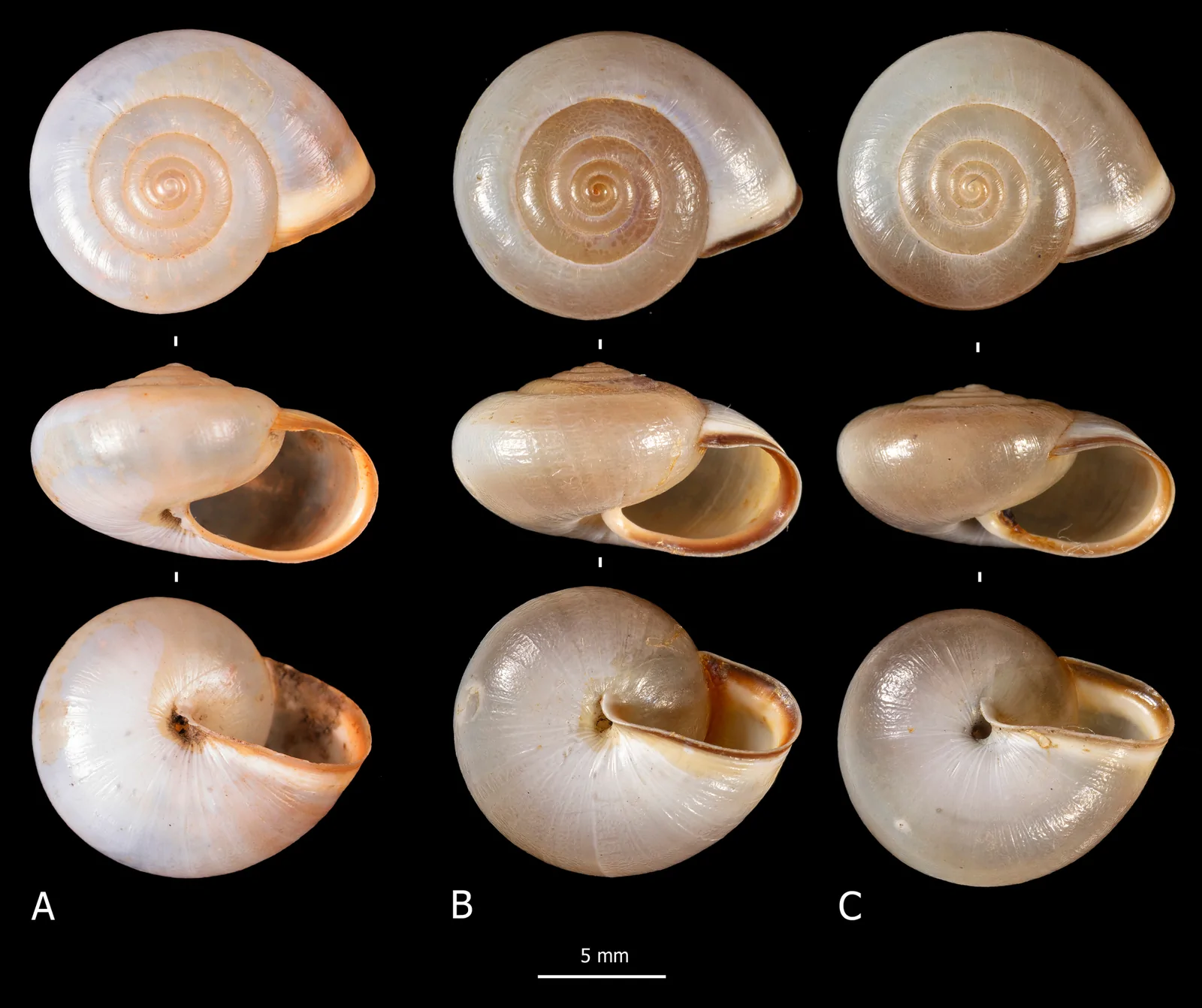
Shells of *Monacha cartusiana*. Syntype from “Gallia” [France], according to the original description; Müller’s collection, Natural History Museum of Denmark (NHMD-1811722) (**A**). Two specimens from Quattrovie, G. Manganelli leg. 24 Sept. 2023 (FGC 55672) (**B, C**). Photo credits: Tom Schiøtte (**A**).

**Figure 4.**
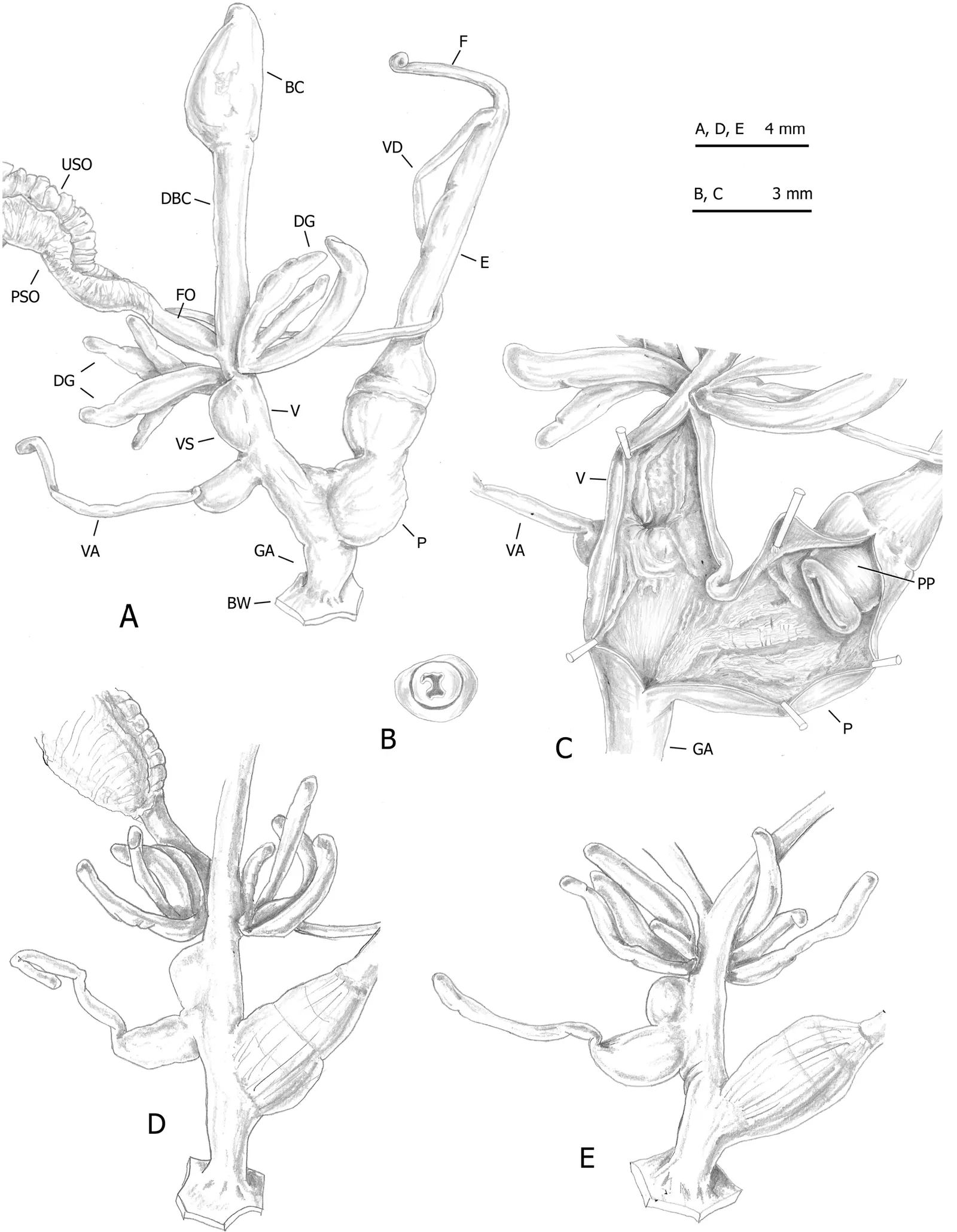
Genital anatomy of *Monacha cartusiana*. Specimens from Quattrovie, G. Manganelli leg. 24 Sept. 2023 (FGC 55672) (**A–C**) and from Valle del Turano, 1.6 km ESE di Turania, A. Hallgass leg. 04 Nov. 2013 (FGC 42970) (**D, E**). Distal genitalia (**A, D, E**), transverse section of apical penial papilla (**B**) and internal structure of vagina and penis (**C**).

Figs 3, 4, 20, 21, 27

Helix cartusiana Müller, 1774: 15. Type locality: France (“Gallia”). Type material: 5 syntypes [shells] in Müller’s collection at the Natural History Museum of Denmark NHMD-1811722 (Tom Schiøtte pers. comm. 10 March 2025) (Fig. 3A).
Helix carthusianella Draparnaud, 1801: 86. Type locality: France. Type material: in Draparnaud’s collection at the Natural History Museum Vienna there are 8 shells (det. var. a/α) and 3 shells (det. var. b/β) all under the same inventory number MO14823_*Helix*_*cartusianella* (Anita Eschner pers. comm. 3 February 2025). Based on photos of one specimen for each lot, var. a/ α corresponds to M. cartusiana and var. b/ β to M. cantiana. Draparnaud (1801: 86) did not report any variety, while Draparnaud (1805: 101) distinguished only the variety b, characterised by “apertura subrotunda”, which matches the shells of M. cantiana. Thus it is possible that the shells labelled “var. a/α” were those on which the species was originally established in 1801, so labelled at a later time to distinguish them from those subsequently assigned to the distinct variety (var. b/β).

##### Material examined.

France • 5 spirit specimens; Cubières-sur-Cinoble, roadside; 42°52'09.7"N, 02°29'06.0"E; 419 m asl; 28 Jun. 2018; M. Proćków leg.; DCBC and MNHW-F.18.38; Italy – **Tuscany** • 5 spirit specimens; Siena province, Quattrovie; 43°24'34.21"N, 11°17'22.76"E; 305 m asl; 11 Jun. 2023; G. Manganelli leg.; FGC 54942 • 6 spirit specimens; same locality; 24 Sept. 2023; G. Manganelli leg.; FGC 55672 • 47 shells, 8 spirit specimens; Siena province, Stazione di Castelnuovo Berardenga; 43°18'27.02"N, 11°28'52.79"E; 210 m asl; 01 Nov. 1981; G. Manganelli leg.; FGC 3430 • 9 spirit specimens; Siena province, Lago di Montepulciano, La Casetta; 43°05'50.21"N, 11°54'59.75"E; 248 m asl; 11 Oct. 1992; G. Manganelli leg.; FGC 42017 – **Latium** • 8 shells, 15 spirit specimens; Rieti province, Lago Lungo; 33TUH20; 370 m asl; 14 Aug. 1966; F. Giusti leg.; FGC 23875 • 4 shells, 1 spirit specimen; same locality; 10 Aug. 1967; F. Giusti leg.; FGC 23877 • 3 spirit specimens; Rieti province, Valle del Turano, 1.6 km ESE of Turania; 42°07'54.84"N, 13°01'39.65"E; 04 Nov. 2013; A. Hallgass leg.; FGC 42970; Poland • 5 spirit specimens; Wrocław, area around the railway tracks; 51°07'03.34"N, 16°59'52.83"E; 20 May 2010; M. Proćków leg.; FGC 41813 • 7 shells; same locality; 15 Sept. 2010; M. Proćków leg.; FGC 41856 • 5 spirit specimens; Wrocław-Pilczyce (Mączna St.), 80 m E of Ślęza River bank; 51°08'30.05"N, 16°56'55.09"E; 120 m asl; 08 Jun. 2023; E. Kowalska leg.; DCBC • 10 spirit specimens; same locality; 17 Sept. 2023; E. Kowalska leg.; DCBC.

##### Morphological description.

Shell (Fig. 3A–C; Manganelli et al. 2025: fig. 3) dextral, small to medium in size, subdiscoidal to subglobose, rather fragile, sub-transparent, milky to horn-yellow in colour, with white and reddish collabral band near aperture. Whorls 5–6, slightly convex and separated by superficial sutures; last whorl ~ 9/10 of shell height, rather dilated and descending slightly near aperture. Aperture rather large, oval to elliptical (AW/AH ~ 1.8) and slightly prosocline. Peristome interrupted, slightly reflected on columella, thickened and with variably evident whitish callous rim on internal outer margin. Umbilicus open, very small to small. Protoconch and teleoconch smooth, with very faint scattered collabral growth lines. Shell dimensions: H – 8.5 ± 1.2 mm; D – 15.2 ± 1.8 mm (*n* = 15).

Female distal genitalia (Fig. 4A, C–E; Manganelli et al. 2025: fig. 11) include free oviduct, bursa copulatrix and its duct, digitiform glands, vagina, and vaginal appendix. Free oviduct short and variably wide. Bursa copulatrix rather large, triangular, shoe- or bean-like, with rather long duct (usually slightly longer than vagina). Vagina short to rather long, without refringent ring but with lateral sac between digitiform glands and vaginal appendix, and with series of narrow irregular internal pleats. Digitiform glands disposed on opposite sides of vagina in two groups of one or two tufts, each with one or two branches. Vaginal appendix rather long, inserted approx. half way along vagina and with dilated basal portion (1/3 of total length) and slender digit-like distal portion (2/3 of total length).

Male distal genitalia (Fig. 4A–E; Manganelli et al. 2025, fig. 11) include vas deferens, flagellum, epiphallus and penis. Vas deferens very long and very slender. Flagellum short, slender, and pointed at tip. Epiphallus rather long and wide. Penis short and wide, enveloped by thin sheath. Penial papilla cylindrical, dilated at tip, with apical opening, usually thin external walls and internal lumen occupied by variably slender central duct, round in section.

Genital atrium (Fig. 4A, C–E; Manganelli et al. 2025: fig. 11) rather short and wide, internally smooth or with variably developed, slightly defined pleats.

##### Molecular description.

Molecular sequences obtained from specimens from France (assumed to be the type locality of *M. cartusiana*) were used to identify the species: COI – ON332652–ON332655, PP947906; 16SrDNA – ON350960–ON350964; ITS2 (with flanking fragments of 5.8SrDNA and 28SrDNA) – ON332790, PP947989–PP947990.

##### Remarks.

*Monacha cartusiana* was the first species of the genus to be described. Unfortunately it was misinterpreted by Draparnaud (1801) who applied this name to a form of *Monacha cantiana* (Montagu, 1803) and described Müller’s species again as *Helix carthusianella* Draparnaud, 1801. Although Férussac (1821b: 69) had already realised the correspondence between *Helix cartusiana* sensu Draparnaud and *Helix cantiana* in the 1820s, the name *Helix carthusianella* continued to be used to denote *Helix cartusiana* up to the 1840s.

*Monacha cartusiana* was also one of the first species of the genus to be studied anatomically. One of the issues disputed by the first investigators was the nature of the vaginal appendix, interpreted as a modified (Moquin-Tandon 1855: 208, pl. 16, fig. 22) or unmodified dart sac (Schmidt 1855: 33). Ashford (1885: 269, pl. 10, fig. 3) was the first author to report the vaginal sac, which he depicted in connection with the vaginal appendix. A connection between vaginal sac and vaginal appendix was also reported by Taylor (1917: 98–99, figs 147, 148), who regarded them as a degenerated dart-sac plus an accessory gland, and by other subsequent authors (Germain 1930: 262, fig. 204; Manga Gonzalez 1983: fig. 41), who described them as a bilobed appendage. Both structures may actually be barely distinct from each other (e.g. Fig. 4A; Manganelli et al. 2025: fig. 11A, D) and more or less united (Fig. 4D, E), however internally they open independently of each other.

Giusti and Manganelli (1987: 127–128) discussed the phylogenetic implications of the two vaginal structures in light of Schileyko’s (1978) hypothesis on the evolution of the dart-sac complex in the helicoideans and hypothesised that species of the nominotypical subgenus of *Monacha* could be separated into two groups, one with vaginal sac and vaginal appendix with enlarged base, the other without vaginal sac and with vaginal appendix without enlarged base. Based on these considerations, Nordsieck (1993) introduced the new subgenus *Eutheba* Nordsieck, 1993 (type species *Helix cantiana* by original designation) for the latter group.

Sequences used in current molecular analyses showed that specimens identified anatomically as *M. cartusiana* were distinct from other species studied in this research (Manganelli et al. 2025: figs 18–21). This applies primarily to mitochondrial gene sequences (Fig. 20; Manganelli et al. 2025: figs 18, 19) obtained from specimens originating from France (COI: ON332653, ON332655, PP947906, and 16SrDNA: ON350961, ON350963, ON350964), Italy (COI: PP947919–PP947923 and 16SrDNA: PP949438–PP949442) and Poland (COI: PP947907–PP947916 and 16SrDNA: PP949426–PP949435). The COI sequences KX507189 and KX507235 as well as 16SrDNA sequences KX495378 and KX495429 obtained by Neiber and Hausdorf (2017) from specimens from Italy and Spain, respectively, were in the same clades with *M. cartusiana*. Although there are ITS2 sequences (with fragments of flanking genes) that can be assigned exclusively to *M. cartusiana* (KX495431, KX495479, ON332790, PP947989, PP947990), in the phylogenetic tree they are grouped in the same clade as the ITS2 sequences of *M. claustralis* (Fig. 21). Pieńkowska et al. (2018a) reported numerous COI and short 16SrDNA sequences in *M. cartusiana*. In the case of the paper by Čejka et al. (2020), there is a discrepancy between the data in this publication and the sequences deposited in GenBank based on it, probably due to hybrid forms of *M. cartusiana* and *M. claustralis* occurring in the Czech Republic. Molecular and anatomical studies are therefore necessary for the correct identification of these two species in central European countries, e.g. Germany, Poland, Czechia, Ukraine, Moldova, Romania (Lesicki et al. 2024; Williams et al. 2024).

*Monacha cartusiana* is reported in almost all of Europe (Bank and Neubert 2017; Pieńkowska et al. 2018a; Ostrovsky 2024) but its native distribution probably only encompasses parts of western and southern Europe where it is quite widespread. On the contrary, it was probably introduced into the UK, where it occurs in the south-eastern sector, and into northern and eastern Europe, where it usually only has scattered occurrences. The introduction into the UK dates back to the Neolithic period (Kerney 1970), whereas that into northern and eastern Europe is recent. Indeed, in the last few decades it has expanded progressively eastward, an increasing number of reports arriving from areas where it was not previously known, such as Czechia (Peltanová et al. 2012), Poland (Pieńkowska et al. 2015), western Russia and southern Belarus (Balashov and Markova 2023; Ostrovsky 2024), inland Ukraine (Gural-Sverlova and Gural 2023), and Georgia (Hausdorf 2000). However some reports may be based on misidentification of *Monacha claustralis*: the two species can only be identified correctly from genital or molecular features (Pieńkowska et al. 2015, 2018a; Neiber and Hausdorf 2017), although identification is even more challenging due to the presence of hybrids (Lesicki et al. 2024; Williams et al. 2024). At the moment, the only reports from central-eastern European countries confirmed on an anatomical and molecular basis come from Germany, Poland, Slovakia, Hungary, Austria, Croatia, Bosnia-Herzegovina, Serbia, and Kosovo (Pieńkowska et al. 2015, 2018a).

Outside Europe, it is said to be found in the United States (North America) (e.g. Robinson 2015), where however the question of species identity remains. GBIF (2025) also reports it from S Sweden, Latvia, Turkey, Lebanon, Israel, and Egypt. A recent review of the global distribution of *M. cartusiana* (Begum et al. 2026) was based solely on populations studied on a molecular basis, without anatomical confirmation. Finally, several papers dealing with molluscicides and pest control indicate *Monacha cartusiana* as a major snail pest in Egypt (Abd El-Wahab 2016; Ahmed et al. 2023) and Saudi Arabia (Murshed et al. 2024) but it is unclear what species are actually involved in these reports.

###### *Monacha parumcincta* (Rossmässler, 1834)

**Figure 5.**
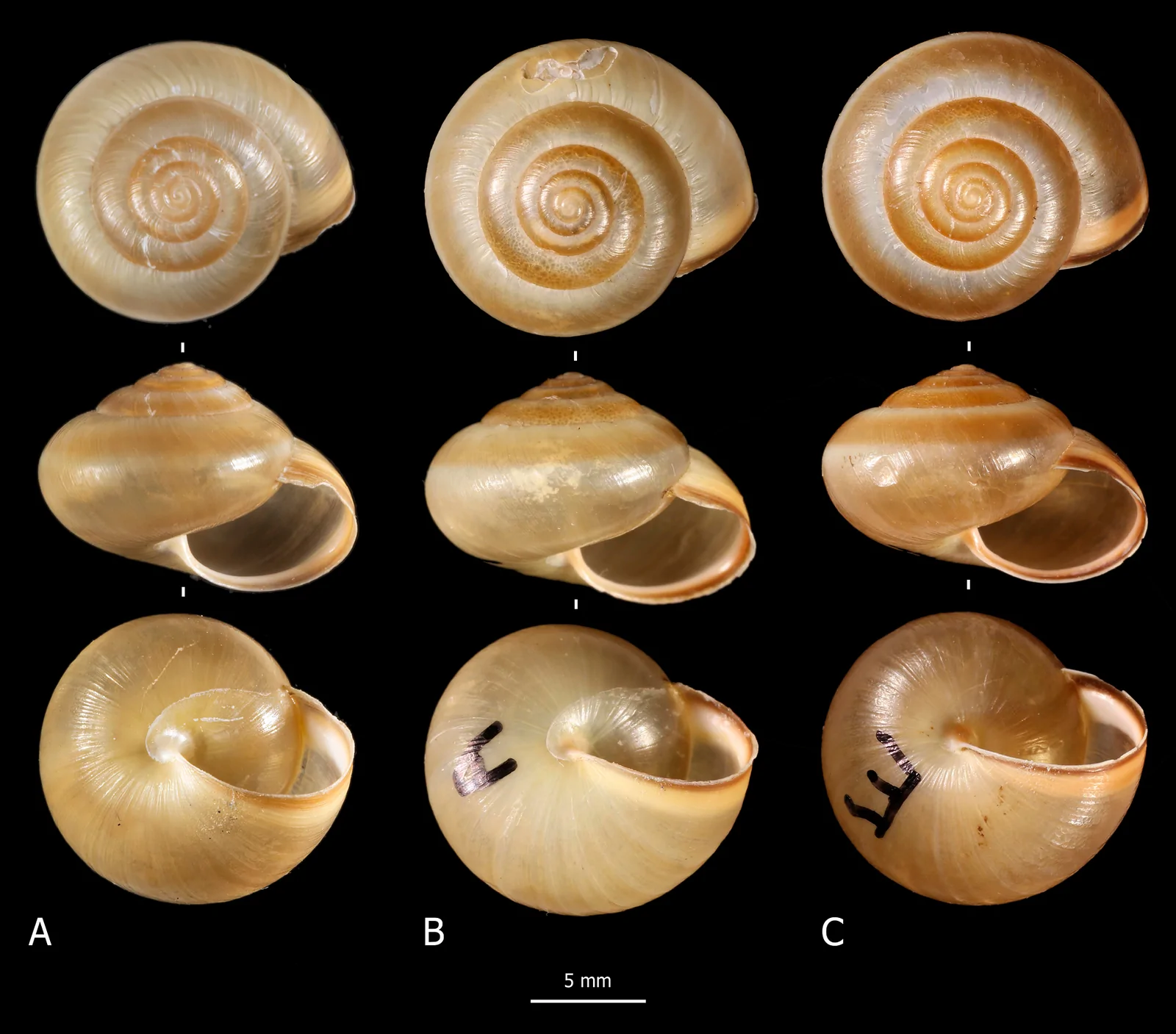
Shells of *Monacha parumcincta* from Corfu. Specimens from Paleokastritsa, along road to monastery of Paleokastritsa, A. Benocci, G. Manganelli & L. Manganelli leg. 25 Oct. 2022 (SMF 383166, neotype) (**A**), (FGC 52333) (**B**) and from Paleokastritsa, Bimbos supermarket, A. Benocci, G. Manganelli & L. Manganelli leg. 25 Oct. 2022 (FGC 52306) (**C**).

**Figure 6.**
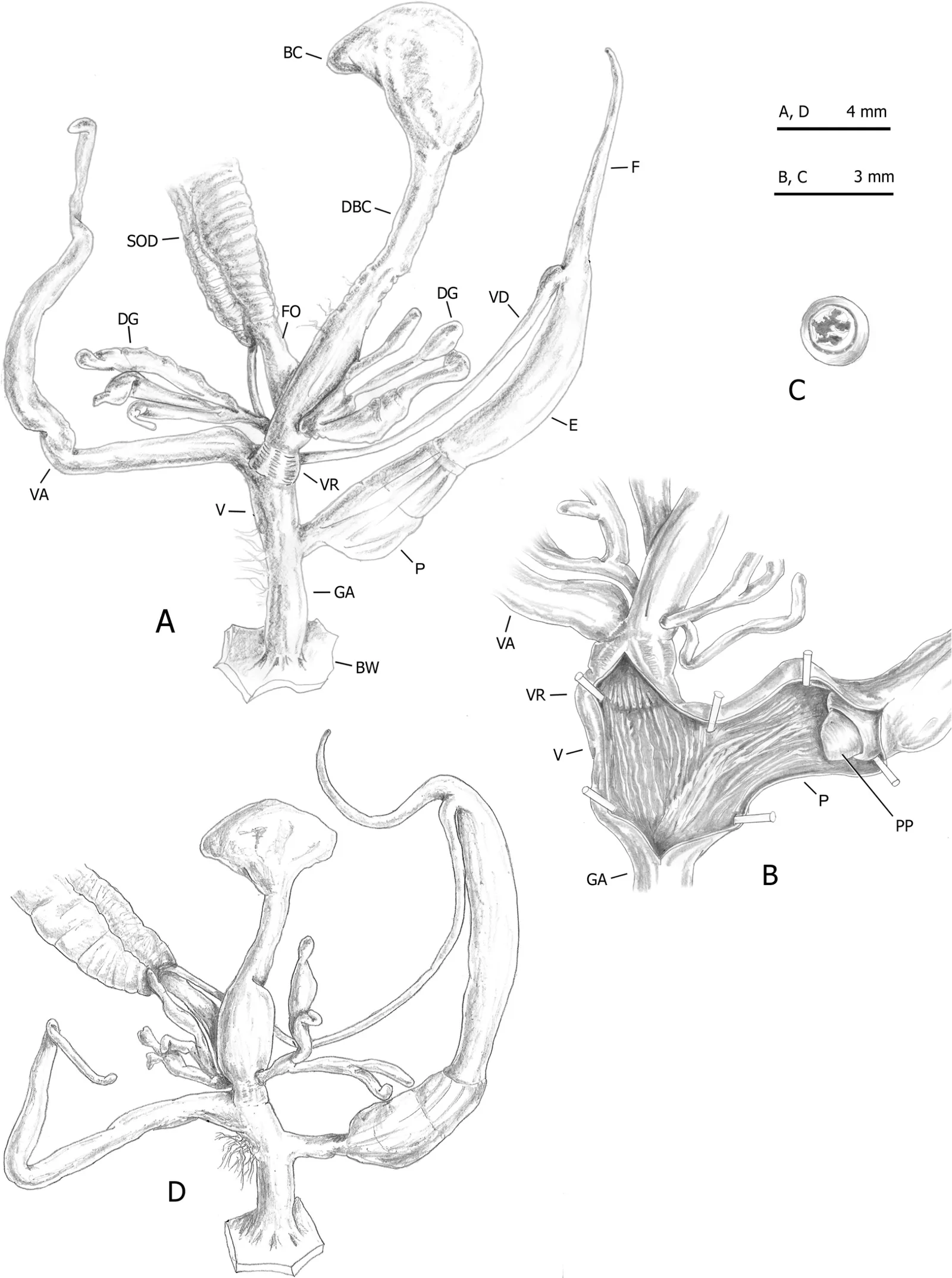
Genital anatomy of *Monacha parumcincta* from Corfu. Specimen from Corfu, Paleokastritsa, along road to monastery of Paleokastritsa, A. Benocci, G. Manganelli & L. Manganelli leg. 25 Oct. 2022 (FGC 52333) (**A–C**) and (SMF 383166, neotype) (**D**). Distal genitalia (**A, D**), internal structure of vagina and penis (**B**) and transverse section of apical penial papilla (**C**).

Figs 5, 6, 20, 21, 27

*Helix parumcincta* Rossmässler, 1834: 5. Type locality: Corfu [Kérkyra], Greece and Dalmatia. Type material: unknown and probably lost (see below and Remarks). Neotype SMF 383166 (Figs 5A, 6D).

**Justification for neotype designation (ICZN Art. 75.3)**

75.3.1. Purpose. A neotype for *Monacha parumcincta* (Rossmässler, 1834) is designated with the purpose of stabilising and clarifying the taxonomic status and type locality of this nominal species-group taxon.

75.3.2. Differential diagnosis. *Monacha parumcincta* is distinguished from the other species of the genus by the characters listed/discussed in the Remarks (below).

75.3.3. Data ensuring recognition of the neotype. The shell is illustrated in Fig. 5A (apical, apertural and umbilical views); distal genitalia are illustrated in Fig. 6D. Dimensions of the shell and distal genitalia are summarised (Table 3). Mitochondrial and nuclear sequence data are deposited in GenBank under accession numbers (Table 1). The following additional features distinguish the neotype: shell with three collabral scars on penultimate whorl and small chip in external margin of peristome above the periphery; body devoid of hind part of foot which was used for molecular analysis; distal genitalia with duct of bursa copulatrix inflated in the first half of its length.

**Table 3.** Shell and genitalia dimensions (in mm) of the neotype of *Monacha parumcincta* (SMF 383166).

**Shell**						**Genitalia**					
SH	SD	AH	AW	UD	NW	DBC	V	VA	F	E	P
9.3	12.9	4.8	7.4	0.0	5 ^1^/_4_	5.7	1.6	15.2	6.1	8.0	5.0

**Figure 7.**
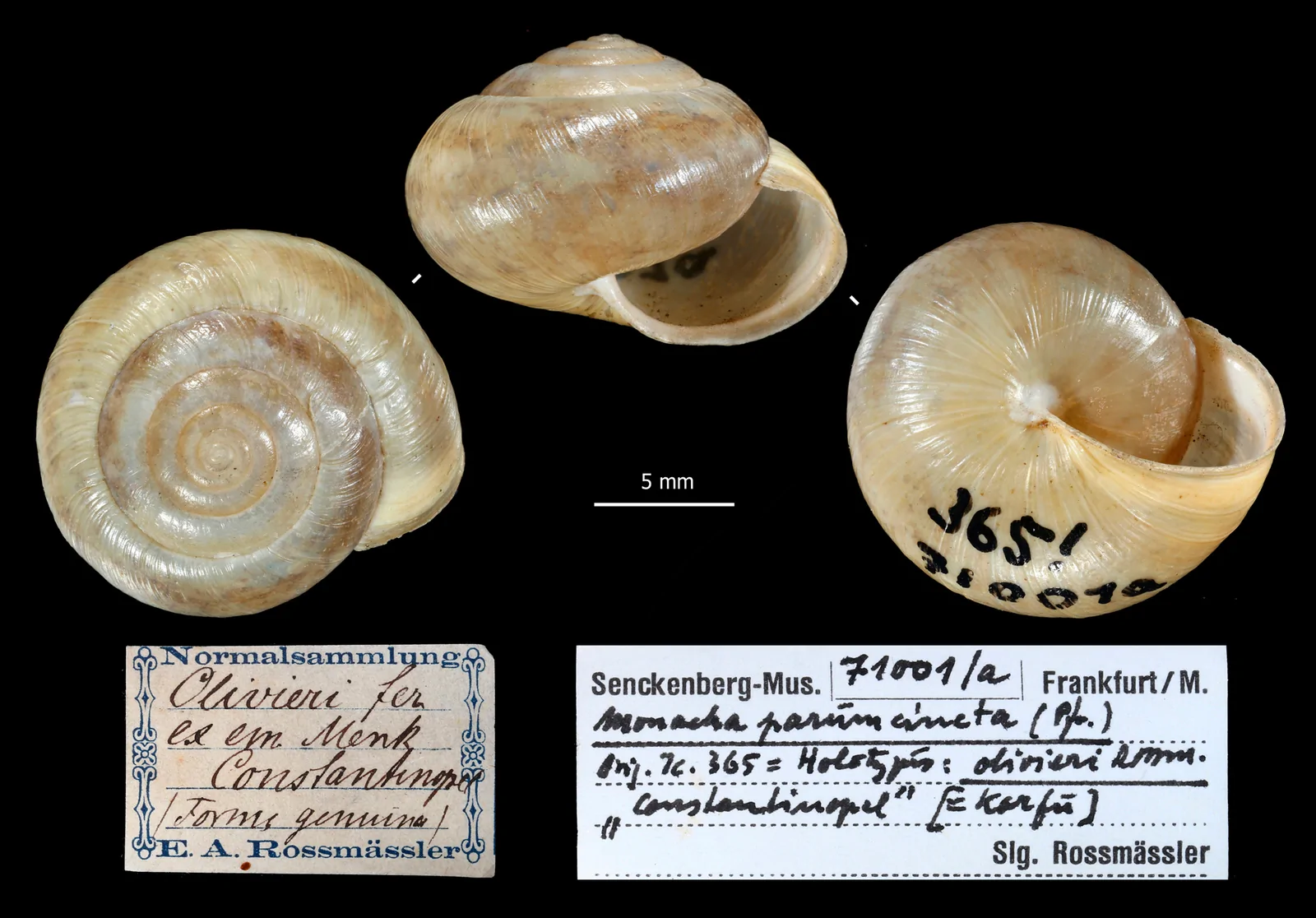
Voucher and type specimens of *Monacha* species. Specimen of *Helix olivieri* figured in Rossmässler’s Iconographie as fig. 365 (SMF 71001/a). Photo credits: Sigrid Hof.

75.3.4. Evidence that the original name-bearing type is lost, and steps taken to trace it. Rossmässler’s collection is at the Senckenberg Forschungsinstitut (Frankfurt am Main) (Zilch 1967). In 2024, we contacted Sigrid Hof of the Section Malacology of SMF for specimens of *Helix parumcincta* or Helix olivieri var. parumcincta from Corfu or Dalmatia in Rossmässler’s collection. Rossmässler’s collection does not include any specimen originally labelled *Helix parumcincta* or Helix olivieri var. parumcincta from Corfu or Dalmatia. Incidentally, a shell (SMF 71001a), labelled “*Olivieri* Fer. | ex em Menk | Costantinopel | (Forma genuina)” by W. Kobelt when he wrote the new labels for Rossmässler’s collection, is regarded by A. Zilch as the specimen in fig. 365 of the Iconographie (see, his handwritten label in Fig. 7) collected on “Korfù” (Sigrid Hof pers. comm. 10 October 2024). The identity and the origin of this specimen is uncertain. Zilch assigned it to *Monacha parumcincta*, labelling it as the holotype of *Helix olivieri* Rossmässler, 1837, which, as explained in the Remarks, is not an available name but only a subsequent usage of *Helix olivieri* Férussac, 1821a. So we can reasonably consider that the original name-bearing type is lost. It must be added that had it been present, and represented by a shell, it would still not have allowed us to establish its identity with absolute certainty, since this group of *Monacha* with imperforate sub-globose shells includes several species that can only be distinguished anatomically (see also the case of *Helix gregaria* Rossmässler, 1839, below).

75.3.5. Consistency with the former name-bearing type. The neotype is consistent with the description of the former name-bearing type characterised by a globose, imperforate shell, with two faded white bands on light brown background (see Rossmässler’s description: “Diese (*H. parumcincta*) ist gut von unserer Art verschieden durch den gänzlichen Mangel des Nabel, kugeligern Bau und stets 2 verwaschene weisse Binden auf hellbraunem Grunde”).

75.3.6. Provenance relative to the type locality. Rossmässler (1834: 5) reported *Helix parumcincta* from Corfu and Dalmatia. Forcart (1965) intended to restrict the type locality to Corfu but type locality restriction can only be the outcome of designation of a lectotype or neotype (ICZN 1999, Art. 76.1), which he did not do. However, since the action of Forcart (1965) has somehow influenced the interpretation of this species, we decided to designate a neotype collected on Corfu. Since the species is widespread on Corfu, the choice fell on a location that was easily accessible and where the species is particularly abundant.

75.3.7. Deposition. The neotype was deposited in the malacological collection of Senckenberg Forschungsinstitut (Frankfurt am Main, Germany), to be kept with what remains of Rossmässler’s collection. The catalogue number is SMF 383166.

**Neotype**. SMF 383166 (Figs 5A, 6D), a specimen collected at Paleokastritsa, along the road to monastery of Paleokastritsa, Corfu [Kérkyra], Greece (39°40'17.43"N, 19°42'04.51"E) by A. Benocci, G. Manganelli and L. Manganelli on 25 October 2022. The body of the neotype was extracted from the shell and dissected to isolate the distal genitalia; the hind part of the foot was used for molecular analysis. Shell and body are preserved separately. The body and distal genitalia are in two distinct 0.5 ml plastic centrifuge tubes with snap caps.

**Topotypes**. FGC 52333 (Figs 5B, 6A–C), 15 spirit specimens collected at Paleokastritsa, along the road to monastery of Paleokastritsa, Corfu [Kérkyra], Greece (39°40'17.43"N, 19°42'04.51"E) by A. Benocci, G. Manganelli and L. Manganelli on 25 October 2022.

**Other material examined**. Greece • 8 spirit specimens; Corfu [Kérkyra], Paleokastritsa, Bimbos supermarket; 39°40'49.64"N, 19°44'12.86"E; 90 m asl; 25 Oct. 2022; A. Benocci, G. Manganelli & L. Manganelli leg.; FGC 52306 • 2 shells, 5 spirit specimens; Corfu [Kérkyra], Pantokrator; 39°44'50.71"N, 19°52'19.23"E; 900 m asl; 24 Oct. 2022; A. Benocci, G. Manganelli & L. Manganelli leg.; FGC 52284.

**Morphological description**. Shell (Fig. 5A–C; Manganelli et al. 2025: fig. 5) dextral, small to medium in size, subglobose to globose, rather robust, opaque, horn-yellow or brownish in colour with two whitish spiral bands – one subsutural, one peripheral – and white and reddish collabral band near aperture. Whorls 5–5^1^/_2_, slightly convex and separated by superficial sutures; last whorl ~ 8/10 of shell height, not dilated and descending slightly near aperture. Aperture rather large, round to oval (AW/AH ~ 1.3) and slightly prosocline. Peristome interrupted, slightly reflected on columella, thickened and with variably evident whitish callous rim on internal outer margin. Umbilicus closed by reflected columellar peristome. Protoconch and teleoconch smooth, with very faint scattered collabral growth lines. Shell dimensions: H – 10.1 ± 0.9 mm; D – 14.4 ± 1.2 mm (*n* = 14).

Female distal genitalia (Fig. 6A, B, D; Manganelli et al. 2025: figs 13–15) include free oviduct, bursa copulatrix and its duct, digitiform glands, vagina, and vaginal appendix. Free oviduct short and variably wide. Bursa copulatrix rather large, triangular, shoe- or bean-like, with long duct (usually much longer than vagina). Vagina short, with refringent ring between digitiform glands and vaginal appendix, and with series of narrow internal pleats, raised in correspondence with the proximal ring. Digitiform glands disposed on opposite sides of vagina in two groups of two tufts, each with one or two branches. Vaginal appendix very long, inserted approx. half way along vagina, without dilated basal portion but tapering progressively towards tip.

Male distal genitalia (Fig. 6A–D; Manganelli et al. 2025: figs 13–15) include vas deferens, flagellum, epiphallus and penis. Vas deferens very long and very slender. Flagellum short, slender and pointed at tip. Epiphallus rather long and wide. Penis short and wide, enveloped by thin sheath. Penial papilla cylindrical, dilated at tip, with apical opening, usually rather thick external walls and internal lumen almost completely occupied by large central duct, round in section.

Genital atrium (Fig. 6A, B, D; Manganelli et al. 2025: figs 13–15) variably short and wide, internally smooth or with variably developed, slightly defined pleats.

**Molecular description**. Neotype sequences (COI – PZ535807, 16SrDNA – PZ539313, ITS2 with flanking fragments – PZ539315) and other nucleotide sequences obtained from specimens from the type locality of *M. parumcincta* are treated as typical for this species: COI – PP947895–PP947905, 16SrDNA – PP949409–PP949419, ITS2 (with flanking fragments of 5.8SrDNA and 28SrDNA) – PP947973–PP947983.

**Remarks**. The second *Monacha* species reported from Corfu has a complex taxonomic and nomenclatural history, due to being related to the interpretation of taxa first established in the distant past as species, varieties or synonyms without adequate description or information. Current use of the specific name “*parumcincta*” to denote a *Monacha* species with globose imperforate shell from the central-eastern Mediterranean goes back to Forcart (1965). Prior to that date, the species was denoted as “*olivieri*” or “*olivierii* var. *parumcincta*”. The status of these nominal taxa was broadly summarised by Welter-Schultes (2012a) and the following remarks are largely from his contribution.

The specific name “*olivieri*” was introduced by Férussac (1821a: 43 quarto edition; 47 folio edition; no. 255 in the species account), as Helix (Helicella) olivieri, without any description, definition, indication or other information. Férussac included three variants (α, β, and γ) in *Helix olivieri*, giving a short definition of each and in the case of γ, also an indication together with a list of occurrences: α) “Majore, pellucens, unicolor” from île de Zante [Zákinthos Greece] (C.^te^ Mercati), Naples [Italy] (D. Ferry) and Gemleck [Gemlik, Turkey] (Olivier); β) “Opaca, alba, fasciata” from Constantinople [Turkey] (Olivier); γ) “Minor; *Helix carthusianella*, β) Draparn., *Hist*., pl. VIII, fig. 3 à 5 [3–4: varieté β of *Helix carthusianella*; 5: *Helix plebeium*]” from Seyde [? Seyde Germany or Sidon, Lebanon], Larnaca [Larnaka, Cyprus]; Baruth [? Baruth Germany or Beirut, Lebanon], Lauzanne [Lausanne, Switzerland] and Neuwied [Germany] (Charpentier); Cette [Sète, France] (Draparnaud).

This nominal species was subsequently described and depicted by Pfeiffer (1828: 25, pl. 6, fig. 4), Michaud (1831: 25) and Rossmässler (1837: 36–37, pl. 27, fig. 365).

Pfeiffer (1828) used the name *Helix olivieri* for a species from Ehrenbreitstein near Koblenz, cited in the synonymy “*Helix Olivieri*, γ, Férussac, Prod. p. 47 No. 255”, and stated that it also occurred at Neuwied, near Koblenz according to Férussac and at Trieste and in Gottschen according to Müller [a mistake, actually Ziegler; see p. 19]; finally Pfeiffer (1828) also stated that a beautiful banded variety, corresponding to “Var. *β*. *Fér.* ”, occurred in Dalmatia.

Michaud (1831: 25) used the name *Helix olivieri* for a species from “Cette [today Sète], les Cabanes, Herault”, cited in the synonymy “*Helix* (*helicella*) *olivieri*, Fér. prod. page 43. n.° 255 *V. G*.” and “Fér. Hist. Moll. Pl. 7. F. 3–5” [a mistake, actually Drap. Hist. Moll.] and observed that this species had initially been dubiously reunited with *Helix carthusianella* by Draparnaud.

Rossmässler (1837: 37) used the name *Helix olivieri* for a species from Dalmatia, Corfu, and “Afrika”, cited in the synonymy “*H. Olivieri Fér*., *prodr*. No. 255” and commented that Pfeiffer (1828) and Michaud (1831) used this name for a form which he regarded as conspecific with *Helix carthusianella* Draparnaud, 1801. Rossmässler cited Férussac’s (1821a) original description, so Rossmässler used *Helix olivieri* Férussac, 1821a subsequently and did not establish a new name *Helix olivieri* Rossmässler, 1837.

As for the other specific name “*parumcincta*”, it was first reported by Menke (1828: 12, 1830: 21), as *Helix parumcincta*, in synonymy of a subordinate taxon (variant “a”) of *Helix olivieri* Férussac, 1821a attributing it to the shell dealer Franz A. Ziegler. This nominal species was subsequently described by Rossmässler (1834) and Mousson (1854).

Rossmässler (1834: 5) reported *Helix parumcincta* from Corfu and Dalmatia, giving a short description (shell globose, imperforate, with two faded white bands on light brown background) and attributing the name to the shell collector and dealer Ludwig Parreyss. He established this species in the discussion of *Helix carthusianella* Draparnaud, 1801, stating that the distinction between the latter and *Helix olivieri* was not clear because Férussac did not give descriptions and figures of both and that Menke thought that *Helix olivieri* was the same as *Helix parumcincta* from Corfu and Dalmatia.

Between 1834 and 1854, “*Helix parumcincta* Parr.” was mentioned as a synonym of *Helix olivieri* by Rossmässler (1837: 37), Pfeiffer (1841: 68, 1842: 93), Jay (1852: 160) and Bourguignat (1853: 25), of *Helix olivieri* [var.] β by Pfeiffer (1848b: 132) or of *Helix olivieri* var. A by Pfeiffer (1848a: 121) always referenced to the unpublished name by Parreyss (and never to Menke, 1828, 1830, or Rossmässler, 1834). In 1854, Mousson (1854: 4, 5) again described Helix olivieri var. parumcincta from southern Italy (Calabria), Sicily, Dalmatia, Corfu and Greece, giving a short description (shell more robust, with two faded white bands, one bordering the suture and the other the periphery, umbilicus closed, basal margin concave, surface more irregular [plus solide; deux bandes blanchâtres, lavées, l’une bordant la suture, l’autre la circonférence des tours; perforation entièrement fermée; bord basal concave; inégalités de la surface plus marquées]), again attributing the name to Parreyss, and stating that it was in the sense used by Menke (1830: 21).

Subsequently *Helix parumcincta* was again reported as a variety of *Helix olivieri* in faunal lists, handbooks, catalogues and so on by many authors such as Mousson (1859a: 16–17, 28; 1859b: 158; 1863: 280), Albers and Martens (1860: 105), Kobelt (1871: 11, 1881: 24), Tryon (1887: 191), Westerlund (1889: 86), Pilsbry (1894: 266), Sturany (1902a: 127–128, 1902b: 403) and Germain (1921: 198–199). However some authors, such as Degner (1927: 65), began to realise that this group of *Monacha* lacked clarity. The puzzle was tackled by Forcart (1965) who undertook the first modern taxonomic nomenclatural revision of the group.

First, Forcart (1965) defined the status of *Helix olivieri* Férussac, 1821a, selecting the specimen depicted by Draparnaud (1805: pl. 7, figs 3, 4) as its lectotype, thus considering this nominal taxon a junior subjective synonym of *Monacha cartusiana*. Regarding its variants, Forcart stated that *Helix olivieri* var. α corresponded to *Monacha cartusiana*, *Helix olivieri* var. β to *Monacha syriaca* (Ehrenberg, 1831) and *Helix olivieri* var. γ to *Monacha cartusiana*. Then he judged *Helix olivieri* Rossmässler, 1837 an invalid name, regarding it a junior homonym of *Helix olivieri* Férussac, 1821a. With regard to *Helix parumcincta*, which he assigned to Pfeiffer, 1848b, he intended to restrict its type locality to Corfu, however without designating a lectotype. Finally he attributed to this species the specimens of “*Theba olivieri*” from southern Italy studied anatomically by Degner (1927: 62–69, fig. 9a–d) and those from Akbes in Syria examined by Hesse (1931: 37–38, pl. 5, fig. 43a–c).

Despite Forcart’s action (1965), the status of *Helix olivieri* is still not definitive, since according to the current edition of the Code, his lectotype designation is invalid because he selected a specimen assigned by Férussac (1821a) to the variant γ and specimens assigned to variants are excluded from the type material of a taxon (ICZN 1999, Art. 72.4.1).

More recent inputs include two contributions with opposite conclusions. The former, by Hausdorf (2000: 78–79), regarded Férussac’s name being unavailable because it is not accompanied by a description, definition or indication (ICZN 1999, Art. 12.1) and concluded that this nominal taxon was made available by Pfeiffer (1828) as a junior synonym of *Monacha cartusiana*. The latter, by Welter-Schultes (2012a: 504), considered Férussac’s name available, because even if there was no description of the “main form”, there were short definitions and a bibliographical reference for the three variants (α, β, and γ), making the name available under Art. 12.1 (taxa require a description and according to Code glossary, a taxon comprises all subordinate taxa).

Welter-Schultes’ interpretation leaves the problem of the type series unsettled, because specimens assigned to variants are excluded from the type series of a taxon (incidentally Férussac’s taxonomic approach to subordinate variants did not include the nominotypical one). This shortfall will probably be addressed and settled in the next Code edition with the new Art.72 (Determination of the type series for names proposed without a description, but with descriptions for distinct variants) (Welter-Schultes 2024). If Art. 72 of the new Code edition is approved, the type specimens of the three variants will be the type series for *Helix olivieri* Férussac, 1821a. This could in turn lead to acceptance of the validity of Forcart’s 1965 lectotype designation, from which it follows that this taxon is a junior synonym of *Monacha cartusiana*.

The authorship of *Helix parumcincta* must be attributed to Rossmässler (1834). Indeed, contrary to Falkner’s (1996) claim, the name cannot be assigned to Menke (1828, 1830) because he reported it as a synonym of variant “a”, for which no name was used as valid (ICZN 1999, Art. 11.6).

Rossmässler (1834: 5) reported *Helix parumcincta* from Corfu and Dalmatia. Forcart (1965) intended to restrict the type locality to Corfu but type locality restriction – a practice used up to the end of the 1950s (cf. Dunn and Stuart 1951; Smith 1953) – can only be the outcome of designation of a lectotype or neotype (ICZN 1999, Art. 76.1). In Rossmässler’s collection at the Senckenberg Forschungsinstitut, Frankfurt am Main, there is no specimen originally labelled *Helix parumcincta* or Helix olivieri var. parumcincta from Corfu or Dalmatia. However Sigrid Hof found a shell (SMF 71001a), labelled by W. Kobelt “*Olivieri* Fer. | ex em Menk | Costantinopel | (Forma genuina)” when he wrote the new labels for Rossmässler’s collection and regarded by A. Zilch as the specimen in fig. 365 of the Iconographie (see Fig. 7) collected on “Korfù” (Sigrid Hof pers. comm. 10 October 2024). Zilch assigned it to *Monacha parumcincta*, labelling it as the holotype of *Helix olivieri* Rossmässler, 1837, which, as explained above, is not an available name but only a subsequent usage of *Helix olivieri* Férussac, 1821a.

So in this state of uncertainty, we selected a neotype in line with Forcart’s first revision in order to define the identity of Rossmässler’s species. The neotype was selected from specimens collected in Corfu, at Paleokastritsa (A. Benocci, G. Manganelli & L. Manganelli leg. 25 October 2022). Data ensuring recognition of the neotype are given above. The neotype is kept in the malacological collection of SMF with the registration number 383166.

Based on Corfu populations, *Monacha parumcincta* is a species of the nominotypical subgenus (according to the subgeneric division proposed by Neiber and Hausdorf 2017) with a globose, imperforate shell and distal genitalia characterised by a very short to short vagina, with refringent ring between digitiform glands and vaginal appendix; vaginal appendix very long, inserted approx. half way along vagina and without dilated basal portion and progressively tapering towards tip.

The most remarkable feature with respect to other species of the nominotypical subgenus is the refringent ring of the proximal vagina. This structure could correspond to the “muscular swelling” described by Hausdorf (2000) in two species from the eastern Pontus: *Monacha comata* Hausdorf, 2000 (Hausdorf 2000: 82, 84, text-fig. 20, map 7, pl. 5, fig. 21) and *Monacha riedeli* Hausdorf, 2000 (Hausdorf 2000: 99, text-fig. 35, map 7, pl. 8, fig. 38) and in one from southeastern Turkey (Hatay province), western Syria and Lebanon: *Monacha saninensis* (Pallary, 1939) (Hausdorf 2000: 99–100, text-fig. 36, map 5, pl. 8, fig. 39); a similar structure is also present in the Sicilian *Monacha gregaria* (Rossmässler, 1839) (Fig. 14A, C, D). The first two species are easily distinguished morphologically from *Monacha parumcincta* by virtue of their hairy umbilicate shell and the voluminous “muscular swelling” of the proximal vagina. Incidentally, Neiber and Hausdorf (2017) assigned *Monacha comata* to the new genus-group taxon *Trichotheba* Neiber & Hausdorf, 2017. The morphological distinction of *Monacha gregaria* is also easy by virtue of the absence in this species of the digitiform glands and the vaginal appendix and the presence of a C-shaped transverse section of the central duct of the penial papilla. On the contrary, morphological distinction with respect to *Monacha saninensis* is problematical: both species have globose imperforate shells and like-structured distal genitalia apart from the insertion of the appendix (mid vaginal in *Monacha parumcincta*; atrial in *Monacha saninensis*).

Mitochondrial (COI and 16SrDNA) and nuclear (ITS2 with 5.8S and 28SrDNA flanking fragments) gene sequences obtained from specimens identified in the Corfu populations clustered in separate clades on phylogenetic trees analysed separately for particular genes (Manganelli et al. 2025: figs 18, 20) or in concatenated sets (Figs 20, 21; Manganelli et al. 2025: figs 19, 21). Hence, our molecular analyses strongly support the conclusion that Corfiot *M. parumcincta* is a distinct species.

There is certainly still much work to do on central-eastern Mediterranean *Monacha* but clarification of the status of *Monacha parumcincta* finally enables us to define the taxonomic and nomenclatural placement of the *Monacha* populations from central-southern Italy so far assigned to this species. Indeed all the data collected (see above) proves that these populations belong to another species, morphologically and molecularly distinct from Corfiot *Monacha parumcincta*, for which the name Helix olivieri var. manfredonica (Kobelt, 1891) is available.

#### Monacha manfredonica

Taxon classificationAnimaliaStylommatophoraHygromiidae

(Kobelt, 1891)

A43EEE7C-DDF2-5084-81CD-545D681B20DE

Figs 8, 9, 10, 11, 12, 20, 21, 27

Helix (Carthusiana) olivieri var. manfredonica Kobelt, 1891: 12, pl. 123, fig. 741. Type locality: bei Manfredonia am Südfusse des Monte Gargano. Type material: 12 syntypes (SMF 6507/11 and 6519/1), including the specimen figured by Kobelt, are kept in Kobelt’s collection at Senckenberg Forschungsinstitut, Frankfurt am Main (SMF 6519/1; Fig. 12).

##### Material examined.

Italy – **Tuscany** • 2 spirit specimens; Pistoia province, Chiesina; 43°54'18.00"N, 10°49'13.63"E; Oct. 2013; A. Hallgass leg.; FGC 41562 • 8 spirit specimens; Arezzo province, A1 highway, rest area Romita east; 43°30'19.55"N, 11°38'54.92"E; Oct. 2013; A. Hallgass leg.; FGC 41561 • 11 spirit specimens; Siena province, SS 73 “Senese-Aretina”, La Casella; 43°18'59.40"N, 11°30'04.20"E; 04 Oct. 2015; G. Manganelli leg.; FGC 44077 – **Latium** • 3 spirit specimens; Rieti province, Valle del Tronto, Umimar S.p.a.; 42°44'23.05"N, 13°15'58.50"E; 30 Sept. 2012; A. Hallgass leg.; FGC 42957 – **Basilicata** • 3 spirit specimens; Potenza province, Gole del Fiume Platano; 33TWE39; 18 Sept. 1984; F. Giusti & G. Manganelli leg.; FGC 24510 • 2 spirit specimens; Potenza province, Lavello; 33TWF64; 07 Sept. 1978; FGC 24499 • 10 spirit specimens; Potenza province, Valle dell’Agri, between Moliterno and Fontana d’Eboli; 40°13'25.49"N, 15°52'17.07"E; Oct. 2012; A. Hallgass leg.; FGC 42962 – **Calabria** • 3 spirit specimens; Cosenza province, Gole del Raganello; 39°49'45.14"N, 16°19'00.62"E; 13 Oct. 2023; W. Renda leg.; FGC 59131 • 5 shells, 3 spirit specimens; Catanzaro province, Monte Tiriolo; 33SXD31; 600–800 m asl; 27 Sept. 1967; F. Giusti leg.; FGC 24435 • 1 spirit specimen; Vibo Valentia province, Certosa di Serra San Bruno; 38°34'01.16"N, 16°19'15.32"E; 17 Sept. 1984; L. Castagnolo, F. Giusti & G. Manganelli leg.; FGC 24508 • 5 shells, 1 spirit specimen; Reggio Calabria province, Monte Consolino; 33SXC26; 17 Sept. 1984; L. Castagnolo, F. Giusti & G. Manganelli leg.; FGC 24462 – **Apulia** • 2 spirit specimens; Foggia province, Rignano Garganico; 33TWG41; 21 Sept. 1957; S. Ruffo leg.; FGC 24490 • 2 shells, 2 spirit specimens; Foggia province, San Giovanni Rotondo; 33TWG51 / 33TWG61; 22 Sept. 1957; S. Ruffo leg.; FGC 24469 • 14 shells, 3 spirit specimens; Foggia province, Coppa di Pulsano; 33TWG71; 11 Sept. 1981; F. Giusti leg.; FGC 24489 • 1 shell, 4 spirit specimens; Foggia province, Macchia; 33TWG81; 20 Sept. 1984; G. Mariano leg.; FGC 24476 • 1 shell, 3 spirit specimens; Taranto province, Gravina di Ginosa; 33TXE49; Oct. 1988; P. Parenzan leg.; FGC 24537 • 1 spirit specimen; Taranto province, Taranto; 33TXE88 / 33TXE98; 03 Dec. 1973; FGC 24449 • 5 spirit specimens; Taranto province, Gravina di Statte; 33TXE89; 02 Dec. 1973; FGC 24494 • 1 shell, 4 spirit specimens; Taranto province, Martina Franca; 33TXF90; 18 Aug. 1978; FGC 24466 • 15 spirit specimens; Lecce province, Monte Mattia; 34TBK83; 25 Nov. 2008; D. Ferreri leg.; FGC 37371 – **Sicily** • 2 shells, 9 spirit specimens; Messina province, Isole Eolie, island of Salina, Monte dei Porri; 33SVC86; 450 m asl; 26 Apr. 1970; F. Giusti leg.; FGC 24459 • 4 shells, 4 spirit specimens; Messina province, Milazzo; 33SWC22; 22 Oct. 1969; FGC 24437.

##### Morphological description.

**Figure 8.**
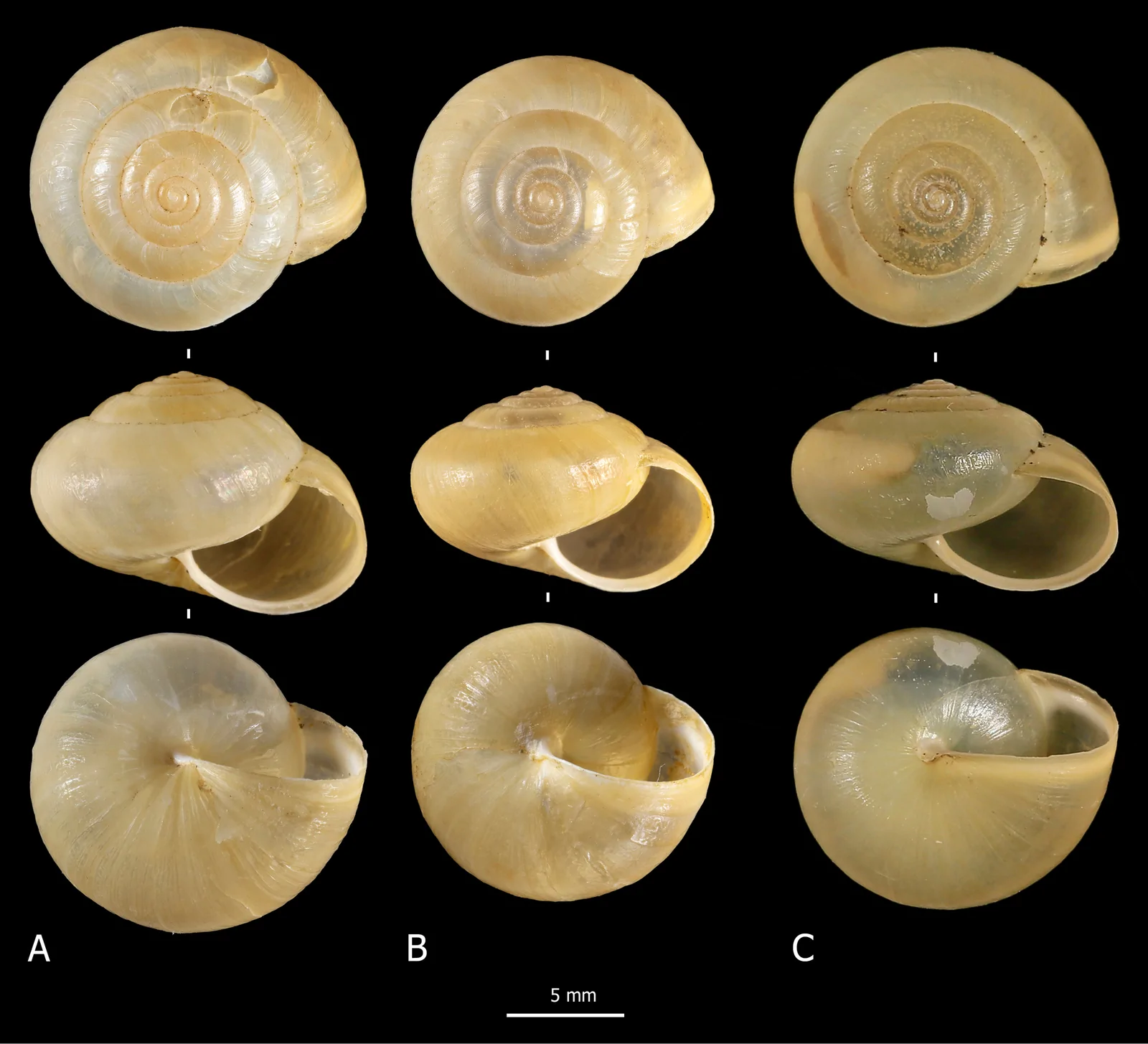
Shells of *Monacha manfredonica*. Specimens from Coppa di Pulsano, F. Giusti leg. 11 Sept. 1981 (FGC 24489) (**A, B**) and A1 highway, rest area Romita east, A. Hallgass leg. Oct. 2013 (FGC 41561) (**C**).

Shell (Figs 8A–C, 12; Manganelli et al. 2025: fig. 6) dextral, small to medium in size, subglobose to globose, rather robust, opaque, horn-yellow or brownish in colour with two whitish spiral bands – one subsutural, one peripheral – and white and reddish collabral band near aperture. Whorls 5–5^1^/_2_, slightly convex and separated by superficial sutures; last whorl ~ 9/10 of shell height, not dilated and descending slightly near aperture. Aperture rather large, round to oval (AW/AH ~ 1.7) and slightly prosocline. Peristome interrupted, slightly reflected on columella, thickened and with variably evident whitish callous rim on internal outer margin. Umbilicus closed by reflected columellar peristome. Protoconch and teleoconch smooth, with very faint scattered collabral growth lines. Shell dimensions: H – 8.5 ± 0.8 mm; D – 12.9 ± 1.4 mm (*n* = 12).

**Figure 9.**
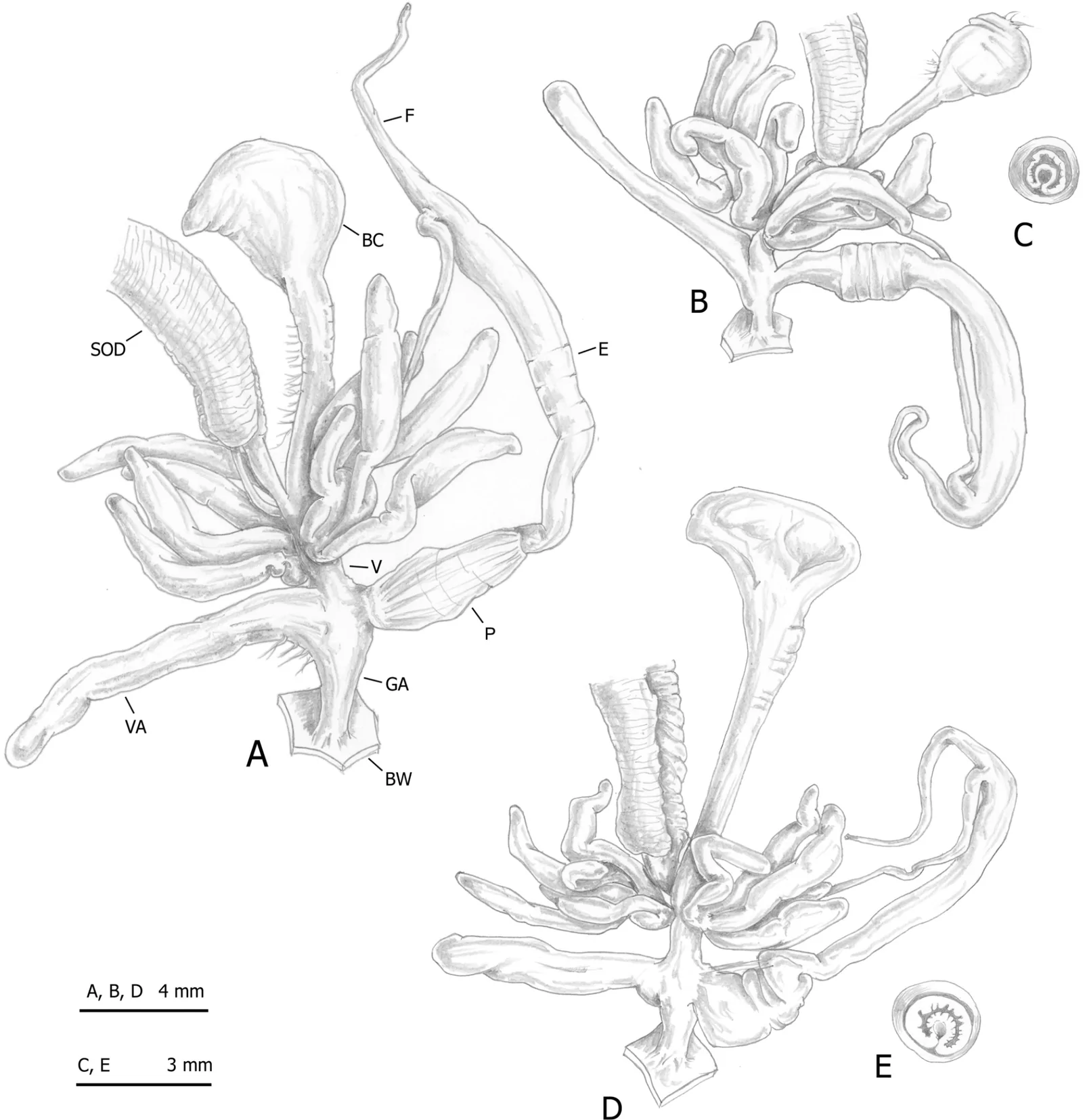
Genital anatomy of *Monacha manfredonica*. Specimens from Coppa di Pulsano, F. Giusti leg. 11 Sept. 1981 (FGC 24489) (**A, D, E**) and San Giovanni Rotondo, S. Ruffo leg. 22 Sept. 1957 (FGC 24469) (**B, C**). Distal genitalia (**A, B, D**) and transverse sections of apical penial papilla (**C, E**).

**Figure 10.**
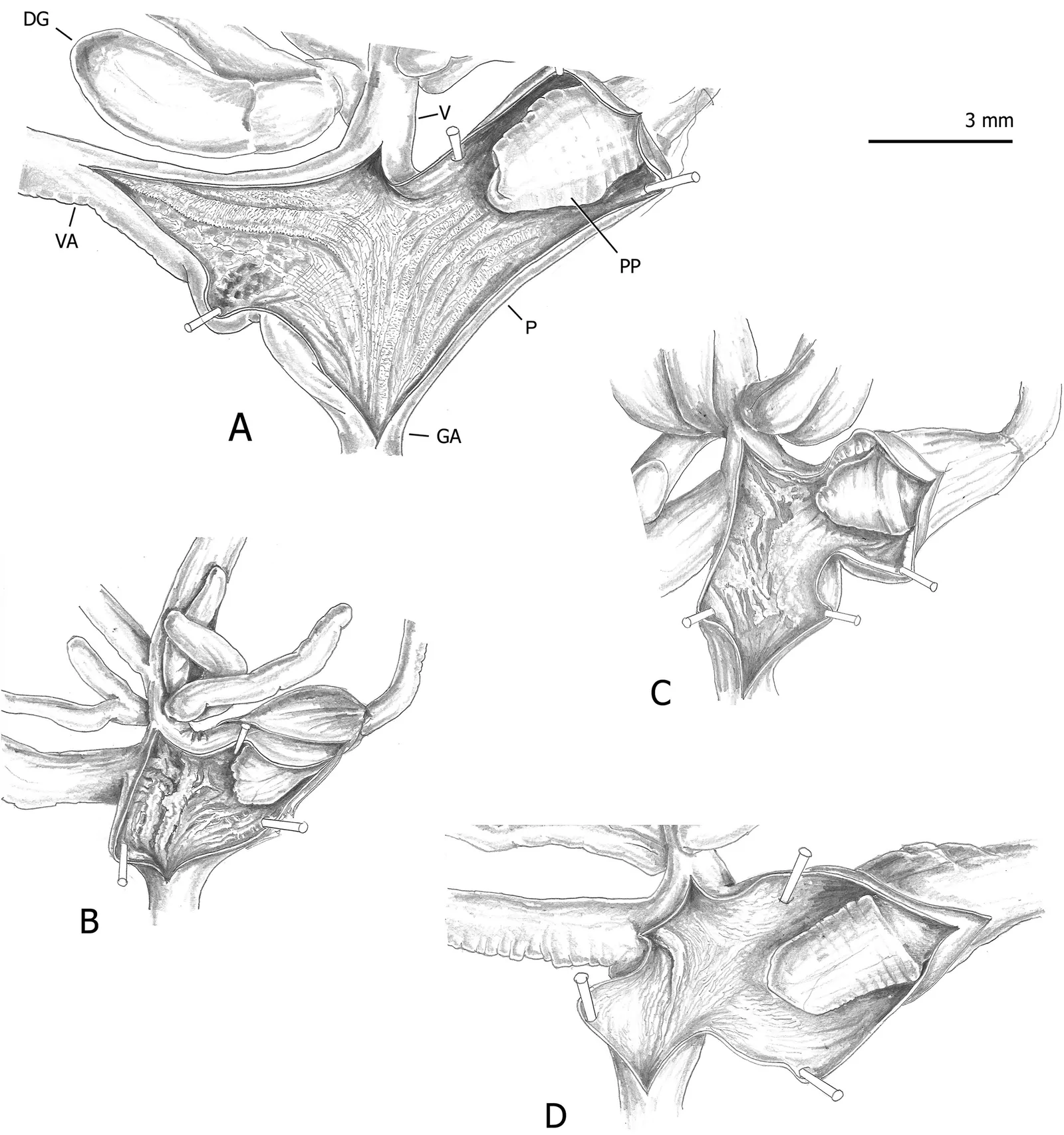
Genital anatomy of *Monacha manfredonica* (internal structure of vagina and penis). Specimens from Valle del Tronto, Umimar S.p.a., A. Hallgass leg. 30 Sept. 2012 (FGC 42957) (**A**); Gravina di Statte, unknown leg. 02 Dec. 1973 (FGC 24494) (**B**); Gravina di Ginosa, P. Parenzan leg. Oct. 1988 (FGC 24537) (**C**); Monte Mattia, D. Ferreri leg. 25 Nov. 2008 (FGC 37371) (**D**).

Female distal genitalia (Figs 9A, 9B, 9D, 10A–D; Manganelli et al. 2025: fig. 16) include free oviduct, bursa copulatrix and its duct, digitiform glands, vagina, and vaginal appendix. Free oviduct short and variably wide. Bursa copulatrix rather large, triangular, shoe- or bean-like, with rather long duct (usually much longer than vagina). Vagina very short, without refringent ring or lateral sac between digitiform glands and vaginal appendix, and with series of narrow irregular internal pleats one of which sometimes more robust. Digitiform glands disposed on opposite sides of vagina in two groups of one or two tufts, each with one or two branches. Vaginal appendix rather short, inserted near distal end of vagina without dilated basal portion, and of uniformly wide calibre.

**Figure 11.**
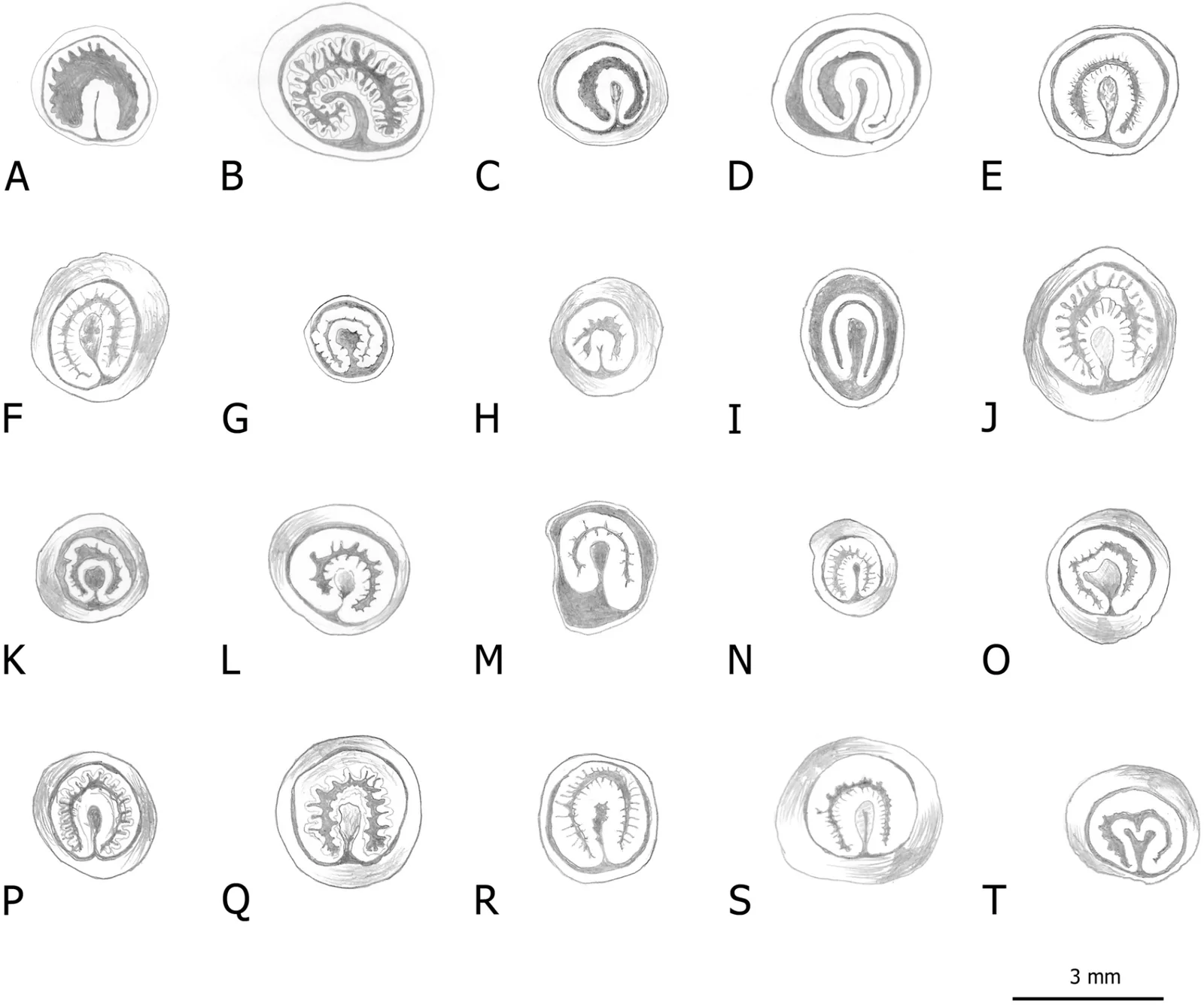
Genital anatomy of *Monacha manfredonica* (sections of penial papilla). Specimens from Chiesina, A. Hallgass leg. Oct. 2013 (FGC 41562) (**A**); A1 highway, rest area Romita east, A. Hallgass leg. Oct. 2013 (FGC 41561) (**B**); Strada statale 73 Senese-Aretina, La Casella, G. Manganelli leg. 04 Oct. 2015 (FGC 44077) (**C**); Valle del Tronto, Umimar S.p.a., A. Hallgass leg. 30 Sept. 2012 (FGC 42957) (**D**); Gole del Fiume Platano, L. Castagnolo, F. Giusti & G. Manganelli leg. 18 Sept. 1984 (FGC 24510) (**E**); Lavello, unknown leg. 07 Sept. 1978 (FGC 24499) (**F**); Valle dell’Agri, between Moliterno and Fontana d’Eboli, A. Hallgass leg. Oct. 2012 (FGC 42962) (**G**); Monte Tiriolo, F. Giusti leg. 27 Sept. 1967 (FGC 24435) (**H**); Certosa di Serra San Bruno, L. Castagnolo, F. Giusti & G. Manganelli leg. 17 Sept. 1984 (FGC 24508) (**I**); Martina Franca, unknown leg. 18 Aug. 1978 (FGC 24466) (**J**); San Giovanni Rotondo, S. Ruffo leg. 22 Sept. 1957 (FGC 24469) (**K**); Coppa di Pulsano, F. Giusti leg. 11 Sept. 1981 (FGC 24489) (**L**); Macchia, G. Mariano leg. 20 Sept. 1984 (FGC 24476) (**M**); Rignano Garganico, S. Ruffo leg. 21 Sept. 1957 (FGC 24490) (**N**); Gravina di Ginosa, P. Parenzan leg. Oct. 1988 (FGC 24537) (**O**); Monte Mattia, D. Ferreri leg. 25 Nov. 2008 (FGC 37371) (**P, Q**); Gravina di Statte, unknown leg. 02 Dec. 1973 (FGC 24494) (**R**); Taranto, unknown leg. 03 Dec. 1973 (FGC 24449) (**S**); Milazzo, unknown leg. 22 Oct. 1969 (FGC 24437) (**T**).

Male distal genitalia (Figs 9A–E, 10A–D, 11A–T; Manganelli et al. 2025: fig. 16) include vas deferens, flagellum, epiphallus and penis. Vas deferens very long and very slender. Flagellum short, slender, and pointed at tip. Epiphallus rather long and wide. Penis short and wide, enveloped by thin sheath. Penial papilla cylindrical, conical at tip, with apical opening, usually rather thick external walls and internal lumen almost completely occupied by large central duct, C-shaped in section.

Genital atrium (Figs 9A, 9B, 9D, 10A–D; Manganelli et al. 2025: fig. 16) rather short and wide, internally smooth or with variably developed, faintly defined pleats.

##### Molecular description.

The following molecular sequences obtained from specimens formerly assigned to Italian *M. parumcincta* could be used for identification of *M. manfredonica*: COI – MG208944, MG208947, MG208949, MG208950, MG208956, MG208959; 16SrDNA – PP949420–PP949425; ITS2 (with flanking fragments of 5.8SrDNA and 28SrDNA) – PP947984–PP947988.

##### Remarks.

Clarifying the status of *Monacha parumcincta* has finally made it possible to address the taxonomic and nomenclatural placement of the *Monacha* populations from central-southern Italy assigned to this species so far. These populations share a globose imperforate shell with *Monacha parumcincta* from Corfu but are anatomically distinct by virtue of lack of the vaginal refringent ring, and by virtue of a C-shaped transverse section of the central duct of the penial papilla and the vaginal appendix usually rather short, without dilated basal portion and of uniformly wide calibre, inserted near the distal end of vagina. Some ratios identified by linear discriminant analysis (LDA), such as E/VA and F/V, are also appropriate for distinguishing the two groups (see Manganelli et al. 2025). Molecular evidence also confirms that the two species are distinct.

Populations with these anatomical features (C-shaped transverse section of the central duct of the penial papilla and the vaginal appendix of uniformly wide calibre) occur in central-southern Italy, northeastern Sicily and the Pontine and the Aeolian archipelagos. Two other *Monacha* species with imperforate shell are currently reported from this area: the western Sicilian *Monacha gregaria* (Rossmässler, 1839) and the southern Italian and Sicilian *Monacha rizzae* (Aradas, 1843), but they differ in anatomy (see below). The Italian populations formerly assigned to *Monacha parumcincta* therefore belong to a third species.

Many taxa have been established for *Monacha* from southern Italy and Sicily by past authors and although revising them is challenging work beyond the aim of this paper, we performed a tentative screening to select a possible name to denote this third species. The fact that it occurs only on the north-eastern coast of Sicily (Giusti 1973; Liberto et al. 2025), where it could have been introduced, induced us to ignore the taxa described from this island and to concentrate on those established for southern Italy.

New nominal taxa for the south Italian *Monacha* with imperforate shell were described by Paulucci (1880), Kobelt (1891), and Bellini (1900, 1915). Paulucci (1880) introduced four varietal taxa from Calabria, i.e. Helix gregaria forma major (p. 70), Helix olivieri var. major (p. 71, pl. 1, figs 6, 6a), Helix olivieri var. pallida (pp. 71, 72), Helix olivieri var. nana (pp. 71, 72, pl. 1, fig. 7), but unfortunately their names are all preoccupied by a plethora of senior homonyms (cf. Sherborn 1922–1931; Richardson 1980; Molluscabase eds 2025).

**Figure 12.**
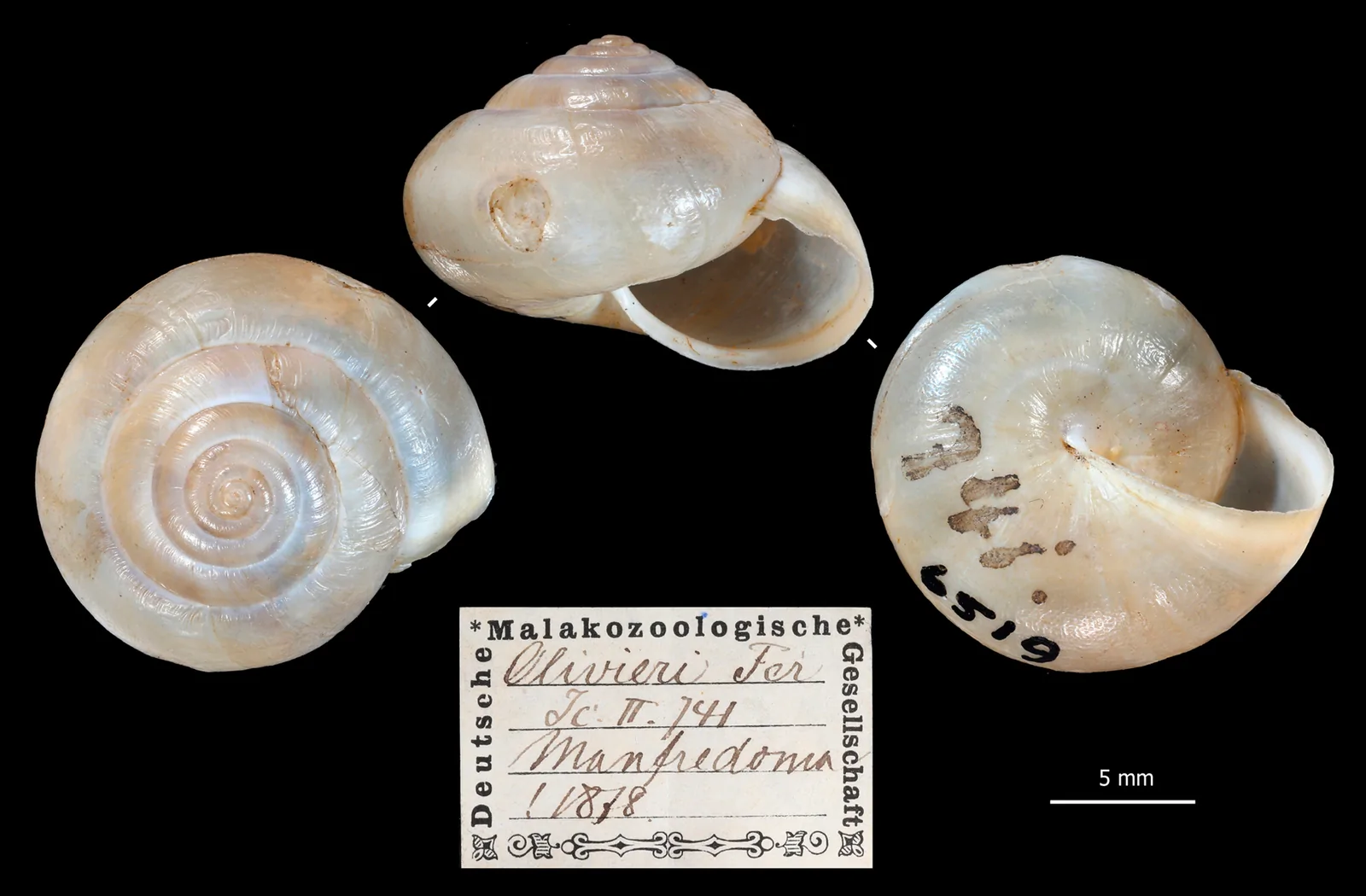
Voucher and type specimens of *Monacha* species. Syntype of Helix olivieri var. manfredonica figured in Rossmässler’s Iconographie as fig. 741 (SMF 6519/1). Photo credits: Sigrid Hof.

Subsequently Kobelt (1891: 12, pl. 123, fig. 741) described Helix (Carthusiana) olivieri var. manfredonica from Gargano peninsula in Apulia, and Bellini (1900, 1915) described *Helix cerioi* (1900: 29) and Helix (Carthusiana) bellinii (1915: 166) from the island of Capri in Campania. Consequently Kobelt’s (1891)Helix olivieri var. manfredonica is the oldest name available for the Italian populations assigned to *Monacha parumcincta* up to now. Shell features of the type material (Fig. 12) and anatomical traits of Gargano specimens (Figs 9, 11K–N) agree with those of the Italian populations formerly assigned to *Monacha parumcincta*.

*Monacha manfredonica* is anatomically distinct due to the C-shaped transverse section of the central duct of the apical penial papilla and vaginal appendix rather short with uniformly wide calibre. A C-shaped transverse section of the central duct of the apical penial papilla has only been found in three other species among those studied so far: *Monacha gregaria* (Fig. 14B, E), *Monacha ocellata* (Roth, 1839) (see Anderson et al. 2018: fig. 6F) and *Monacha* sp. 3, a large, undetermined species from central and southern Calabria (Figs 25B, 26B–G).

*Monacha* specimens with these features are known from Tuscany (many localities), Latium (Valle del Tronto), Apulia (many localities), Basilicata (some localities), Calabria (some localities), Sicilia (Milazzo and Tindari), and the Pontine and Aeolian archipelagos. The populations of this species show remarkable shell variations that mainly concern size, but also colour and hairs or faded bands and a slit-like umbilicus. At present it is not clear whether these differences are due to inter-population variation or to phylogenetic divergence which could be indicative of some differentiation into subclades, as in the case of *Monacha cantiana* (see Pieńkowska et al. 2018b, 2019). The section of the penial papilla also varies considerably between specimens/populations, in particular: the thickness of the external walls and the central duct; the width of the lumen of the central duct and the space between the external walls and those of the central duct; the number and size of pleats covering the internal walls of the central duct. However, the overall appearance of the apical penial papilla section is constant (Fig. 11).

Our molecular studies have shown that the specimens from the Italian populations identified as *Monacha parumcincta* belong to a species distinct from *M. claustralis*, *M. cartusiana*, *M. pantanellii* and *M. cantiana* s.l. and, above all, from the populations of *M. parumcincta* occurring on Corfu (Figs 20, 21; Manganelli et al. 2025: figs 18–21). This is consistent with the results of morphological studies (primarily the anatomy of the proximal genitalia). Sequences of COI (MG208944–MG208959), 16SrDNA (AY741418, MG209016–MG209030), H3 (MG209061–MG209071), ITS2 (MH137985–MH137992) deposited in GenBank for *M. parumcincta* in our previous studies (Manganelli et al. 2005; Pieńkowska et al. 2018b) must be assigned to *M. manfredonica* now. Similarly, the 16SrDNA (PP949420–PP949425) and ITS2 with flanking fragments of 5.8S and 28SrDNA (PP947984–PP947988) sequences deposited in GenBank for Italian *M. parumcincta* (Manganelli et al. 2025) represent *M. manfredonica*.

Historically this species occurred in central-southern Italy, the northern limit of its geographical range coinciding with central-northern Latium, according to some misidentified reports of *Helix gregaria* or *Helix olivieri* dating back to the late 19^th^ – early 20^th^ centuries (Statuti 1882; Lepri 1910). In the early 1990s it was found in southern Tuscany and in the late 1990s in central Tuscany. Its range seems to be gradually expanding northwards; in central-southern Tuscany today, it is the most common, abundant, and widespread *Monacha* species. Its distribution on the Adriatic side of Italy, north of Apulia, is much less known. Outside central and southern Italy, it has only been reported from central Liguria (Birindelli et al. 2020) but the report is based on a misidentification of specimens of *Monacha cf. rizzae*.

#### Monacha gregaria

Taxon classificationAnimaliaStylommatophoraHygromiidae

(Rossmässler, 1839)

4C8C7A80-7279-5004-ACEA-1EF8C832B9C6

Figs 13, 14, 15 B

Helix gregaria Rossmässler, 1839: 7, pl. 43, fig. 569. First introduced as a synonym of Helix onychina Rossmässler, 1839 and then reported in the comments as its variety, with a short diagnosis. Type locality: “Sicilien”. Type material: a syntype in Rossmässler’s collection, Senckenberg Forschungsinstitut, Frankfurt am Main (SMF 71106/1) (Fig. 15B).

##### Material examined.

Italy – **Sicily** • 10 shells; Trapani province, Island of Favignana, Bosco; 33STC60; 25 Mar. 1983; G. Manganelli leg.; FGC 17689 • 1 spirit specimen; Trapani province, Island of Levanzo; 33STC60 / 33STC61; 28 Oct. 1967; S. Riggio leg.; FGC 17703 • 3 shells, 14 spirit specimens; Trapani province, Island of Levanzo, Cala Tramontana; 33STC61; 01 Apr. 1985; G. Manganelli leg.; FGC 17695 • 6 shells, 2 spirit specimens; Trapani province, Calatafimi; 33SUB19; 12 Sept. 1984; F. Giusti & G. Manganelli leg.; FGC 24777 • 2 spirit specimens; Trapani province, Monte Inici, Pizzo Brando; 33SUC10; 03 Apr. 1985; P.P. Fanciulli, E. Malatesta & G. Manganelli leg.; FGC 24486 • 3 shells, 4 spirit specimens; Palermo province, Lo Zucco, near Giardinello; 33SUC31; Oct. 1979; V.E. Orlando leg.; FGC 24775 • 1 spirit specimen; Palermo province, Monte Pellegrino, eastern slope; 33SUC52; 01 May 1972; V.E. Orlando leg.; V.E. Orlando collection; specimen reported by Giusti (1973: fig. 37A) • 9 spirit specimens; Palermo province, Monte Pellegrino, near Arenella; 33SUC52; 24 Apr. 1979; V.E. Orlando & S. Palazzi leg.; FGC 24774 • 1 spirit specimen; Palermo province, Monte Pellegrino, near Santuario di Santa Rosalia; 33SUC52; 28 Oct. 1985; I. Sparacio leg.; FGC 24504.

##### Morphological description.

**Figure 13.**
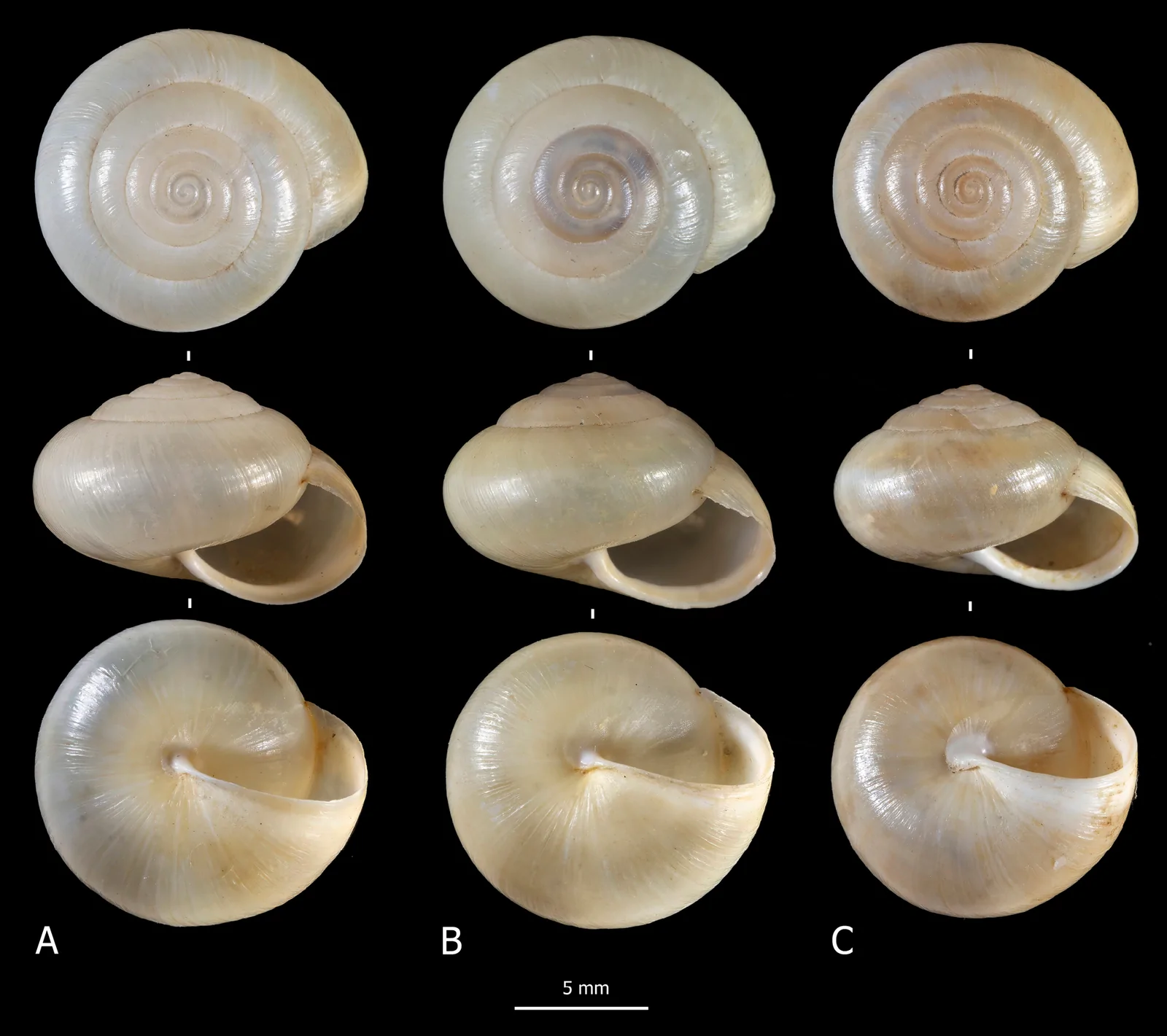
Shells of *Monacha gregaria*. Specimens from Calatafimi, F. Giusti & G. Manganelli leg. 12 Sept. 1984 (FGC 24777) (**A–C**).

Shell (Figs 13A–C, 15B) dextral, small to medium in size, subglobose to globose, rather robust, opaque, whitish to yellowish in colour without spiral bands or collabral band near aperture. Whorls 5–5^1^/_2_, slightly convex and separated by superficial sutures; last whorl ~ 8/10 of shell height, not dilated and descending slightly near aperture. Aperture rather large, round to oval (AW/AH ~ 1.6) and slightly prosocline. Peristome interrupted, slightly reflected on columella, thickened and with evident whitish callous rim on internal outer margin. Umbilicus closed by reflected columellar peristome. Protoconch and teleoconch smooth, with very faint scattered collabral growth lines. Shell dimensions: H – 7.8 ± 1.5 mm; D – 11.7 ± 1.4 mm (*n* = 18).

**Figure 14.**
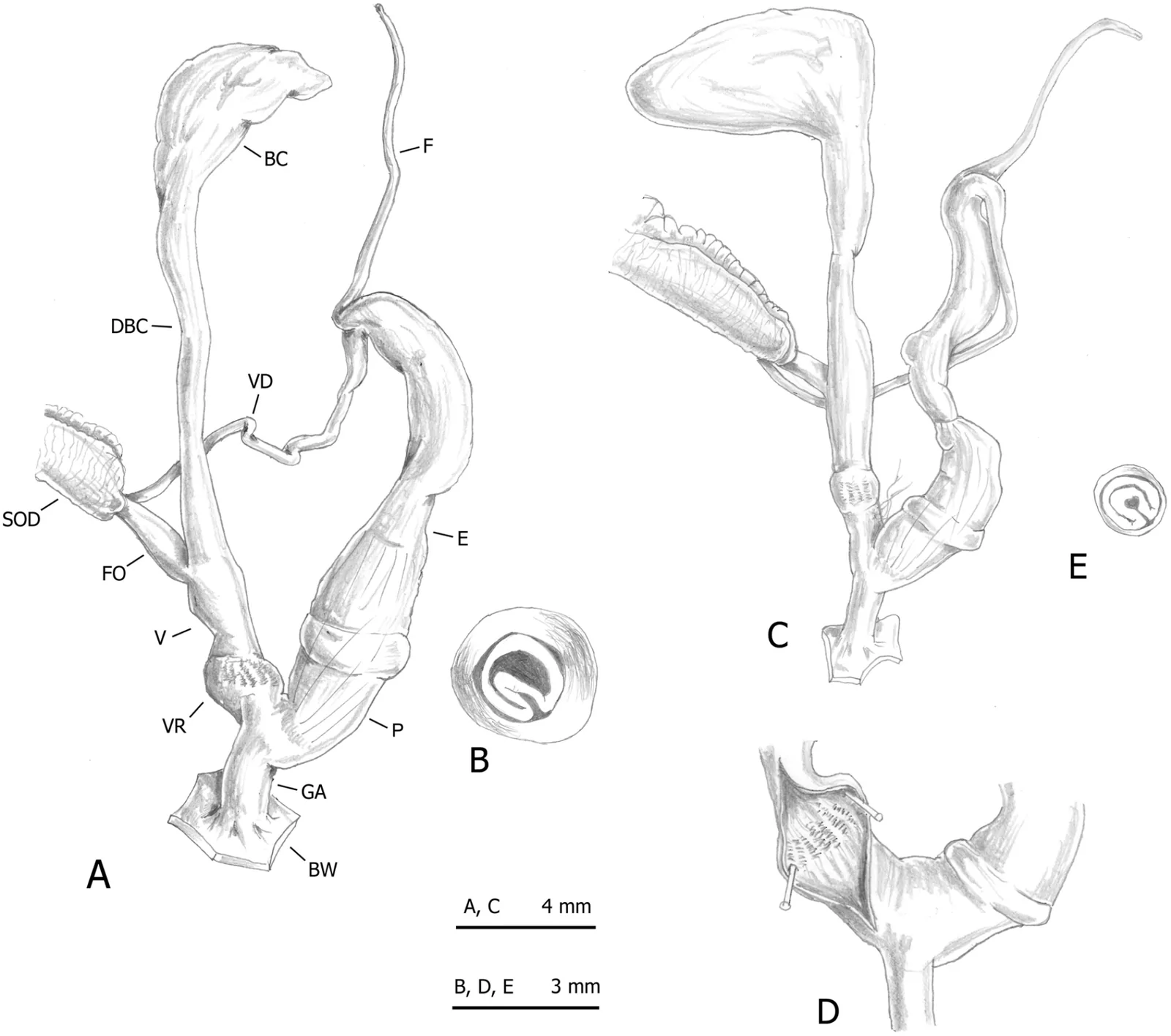
Genital anatomy of *Monacha gregaria*. Specimens from Calatafimi, F. Giusti & G. Manganelli leg. 12 Sept. 1984 (FGC 24777) (**A, B**) and Lo Zucco, V.E. Orlando leg. Oct. 1979 (FGC 24775) (**C, D, E**). Distal genitalia (**A, C**), transverse sections of apical penial papilla (**B, E**) and internal structure of distal vagina, level with muscular ring (**D**).

Female distal genitalia (Fig. 14A, C, D) include free oviduct, bursa copulatrix and its duct, and vagina. Free oviduct short and variably wide. Bursa copulatrix rather large, triangular, shoe- or bean-like, with long duct (usually slightly longer than vagina). Vagina rather long with refringent muscular ring, half its length, and with series of narrow internal pleats. Digitiform glands absent. Vaginal appendix absent.

Male distal genitalia (Fig. 14A–E) include vas deferens, flagellum, epiphallus and penis. Vas deferens very long and very slender. Flagellum short to rather long, slender, and pointed at tip. Epiphallus rather long and wide. Penis short and wide, enveloped by thin sheath. Penial papilla cylindrical, conical at tip, with apical opening, usually rather thick external walls and internal lumen almost completely occupied by large central duct, C-shaped in apical section.

Genital atrium (Fig. 14A, C, D) rather short and wide, internally smooth or with variably developed, slightly defined pleats.

##### Molecular description.

No data available, but see Remarks.

##### Remarks.

This species was first introduced as *Helix gregaria* in the synonymy of *Helix onychina* Rossmässler, 1839 (7, pl. 43, figs 568, 569) and then cited in the paragraph on varieties, with a short diagnosis (small, fairly monochromatic, but otherwise not different variety) and a fig. (pl. 43, fig. 569). Rossmässler (1839) specified that it is reported by Parreyss as “*H. gregaria* Z.” and that *Helix onychina* occurs at “Algier” and its variety in Sicily. Rossmässler did not make clear whether he regarded *H. gregaria* as part of his own classification; however, there is no unambiguous evidence that he did not. So the name should be regarded as having been made available at this occasion.

**Figure 15.**
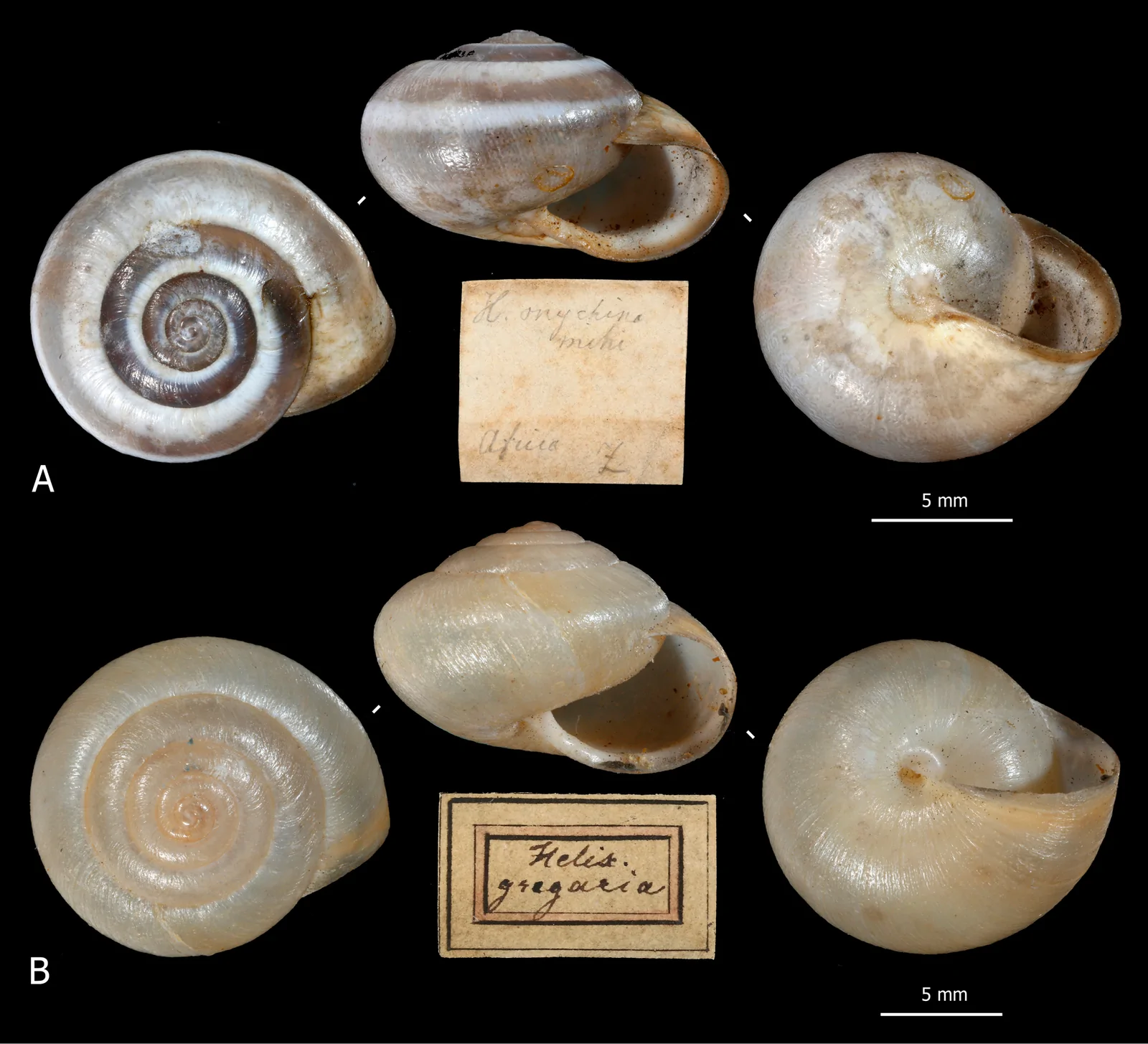
Voucher and type specimens of *Monacha* species. Syntype of *Helix onychina* figured in Rossmässler’s Iconographie as fig. 568 (SMF 70996/1) (**A**). Syntype of *Helix gregaria* figured in Rossmässler’s Iconographie as fig. 569 (SMF 71106/1) (**B**). Photo credits: Sigrid Hof.

The “main form” of *Helix onychina* described from Algiers has never since been found in Algeria (Bourguignat 1864: 150). It could have been described on mislabelled material from the eastern Mediterranean: indeed Ziegler’s original label mentions “Africa”, not “Algier” (Fig. 15A). Bourguignat stated that it was similar to *Helix syriaca* and Tryon (1887: 197), Westerlund (1889: 87), Pilsbry (1894: 267) and Germain (1921: 193) mentioned it as a synonym of *Helix syriaca*.

*Helix gregaria* has been variously interpreted in the past: a variety of *Helix syriaca* (e.g. Westerlund 1876: 62), a variety of *Helix olivieri* (e.g. Westerlund 1889: 86; Pilsbry 1894: 266; Germain 1921: 203–204) and a distinct species (e.g. Tryon 1887: 196; Bellini 1900: 38).

Hesse (1931: 39, pl. 15, fig. 120) identified it as a *Monacha* species characterised by absence of digitiform glands and vaginal appendix, based on the study of a specimen from Realmonte (south-western Sicily). Other specimens examined by him from Segesta and Santa Maria di Gesù near Palermo (western Sicily) were juvenile, while one from Tropea (southern Calabria) perfectly matched that from Realmonte (but see below).

Forcart (1965) confirmed the validity of the species, moved it to the genus-group taxon *Ashfordia* Taylor, 1917, and reported it from several sites in southern Italy based on specimens identified from shells. Subsequently other reports based on anatomical identifications were mentioned by Giusti (1973: fig. 37A) from Monte Pellegrino (western Sicily) and by Pintér (1977) from Monte Santa Caterina, Favignana (Egadi archipelago, western Sicily). Pintér moved it from the genus-group taxon *Ashfordia* to the new genus-group taxon *Szentgalya* Pintér, 1977.

The type material consists of one syntype kept in Rossmässler’s collection at Senckenberg Forschungsinstitut, Frankfurt am Main (SMF 71106/1) (Fig. 15B). Unfortunately, it does not allow the actual identity of the species to be established. If it was indeed collected in Sicily, this shell could belong to more than one species, such as *Monacha gregaria*, *Monacha rizzae*, *Monacha cf. rizzae*. Given this uncertainty, it is preferable to accept the interpretation of this nominal taxon given by Hesse (1931) and Forcart (1965), and to continue to use the name *Helix gregaria* for a Sicilian *Monacha* species lacking vaginal appendix and digitiform glands.

Although *Monacha gregaria* has been reported from various regions and archipelagos of central-southern Italy (Alzona 1971, with references), a *Monacha* species with globose imperforate shell and genitalia lacking digitiform glands and vaginal appendix is currently only known from central western Sicily and the Egadi archipelago. The only report outside this area, that of Hesse (1931) from Tropea, is probably a mistake: indeed Hesse (1931: 38) said he received three specimens from Tropea from F. Settepassi, only one of which was adult, but he reported it as “*Theba bicinta*” (cf. Hesse 1931: 38–39, pl. 5, fig. 39a, b).

Neiber and Hausdorf (2017) sequenced a *Monacha* sp. from “Macari towards San Vito Lo Capo” (ZMH 92200-3484), an area where to our knowledge only *Monacha gregaria* is known. This specimen (Figs 20, 21: Sicilian *Monacha* sp. 2) groups into a clade which contains all the specimens of *Monacha manfredonica* examined as sister-group. Unfortunately, examination of this specimen did not allow us to clarify its identity. Were it confirmed that the specimen belongs to *Monacha gregaria*, then this species would be a *Monacha* (s.s.) without digital glands and vaginal appendix and should therefore not be included in a distinct genus-group taxon (i.e. *Szentgalya*).

From a morphological point of view, *Monacha gregaria* is the most easily recognised *Monacha* species of those living in Italy, due to the absence of digitiform glands and vaginal appendix.

#### Monacha rizzae

Taxon classificationAnimaliaStylommatophoraHygromiidae

(Aradas, 1843)

842CF776-5E0F-5009-A4D2-A2F2FE3B096D

**Figure 16.**
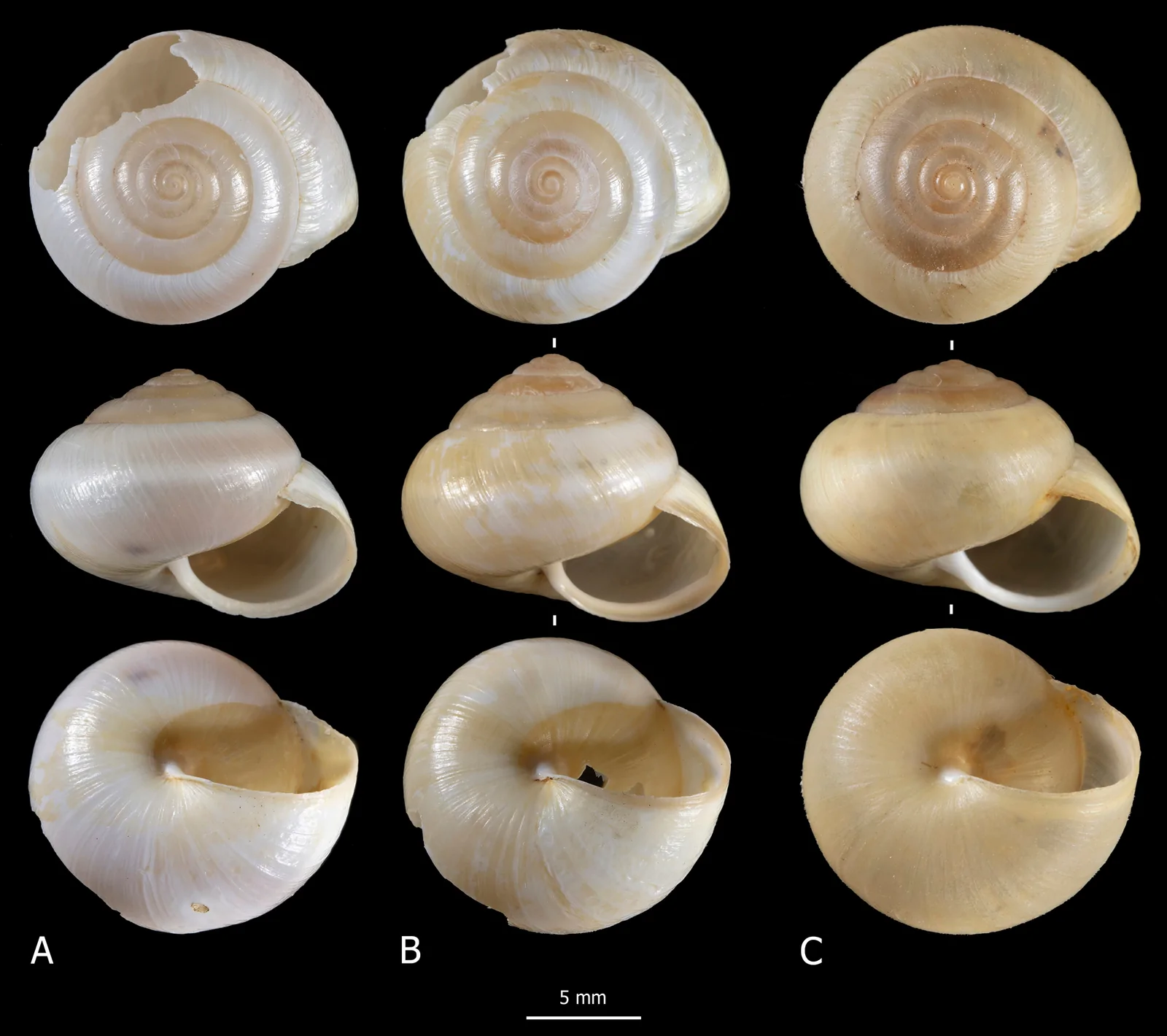
Shells of *Monacha rizzae* and *Monacha cf. rizzae*. Specimens of *Monacha rizzae* from Fontane Bianche, A. Giudice leg. 21 Oct. 1984 (FGC 24485) (**A, B**), and *Monacha cf. rizzae* from Monte Pellegrino, F. Giusti & G. Manganelli leg. 14 Sept. 1984 (FGC 24433) (**C**).

**Figure 17.**
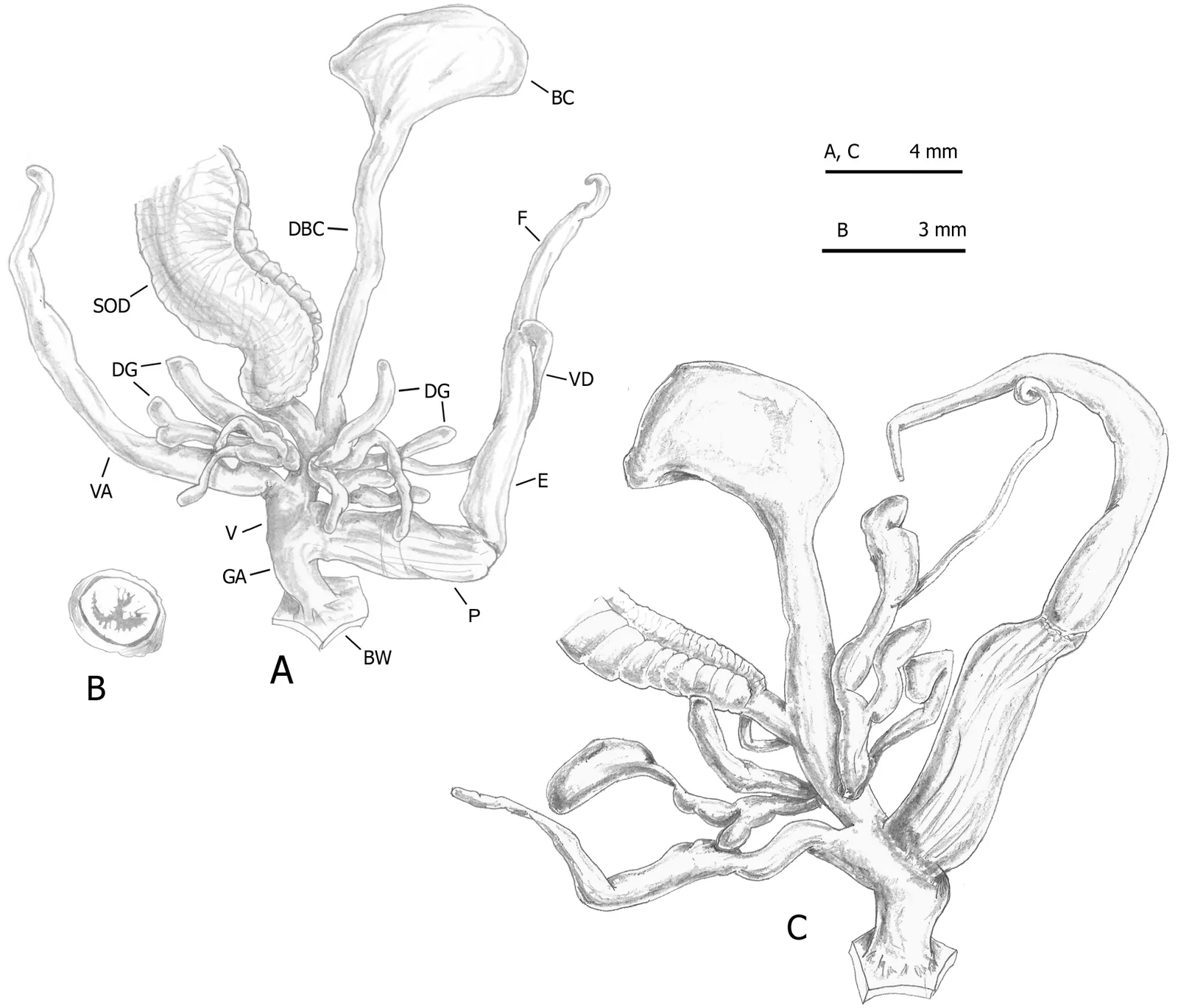
Genital anatomy of *Monacha rizzae* and *Monacha cf. rizzae*. Specimens of *Monacha rizzae* from Fontane Bianche, A. Giudice leg. 21 Oct. 1984 (FGC 24485) (**A, B**), and *Monacha cf. rizzae* from Monte Pellegrino, F. Giusti & G. Manganelli leg. 14 Sept. 1984 (FGC 24433) (**C**). Distal genitalia (**A, C**) and transverse section of apical penial papilla (**B**).

**Figure 18.**
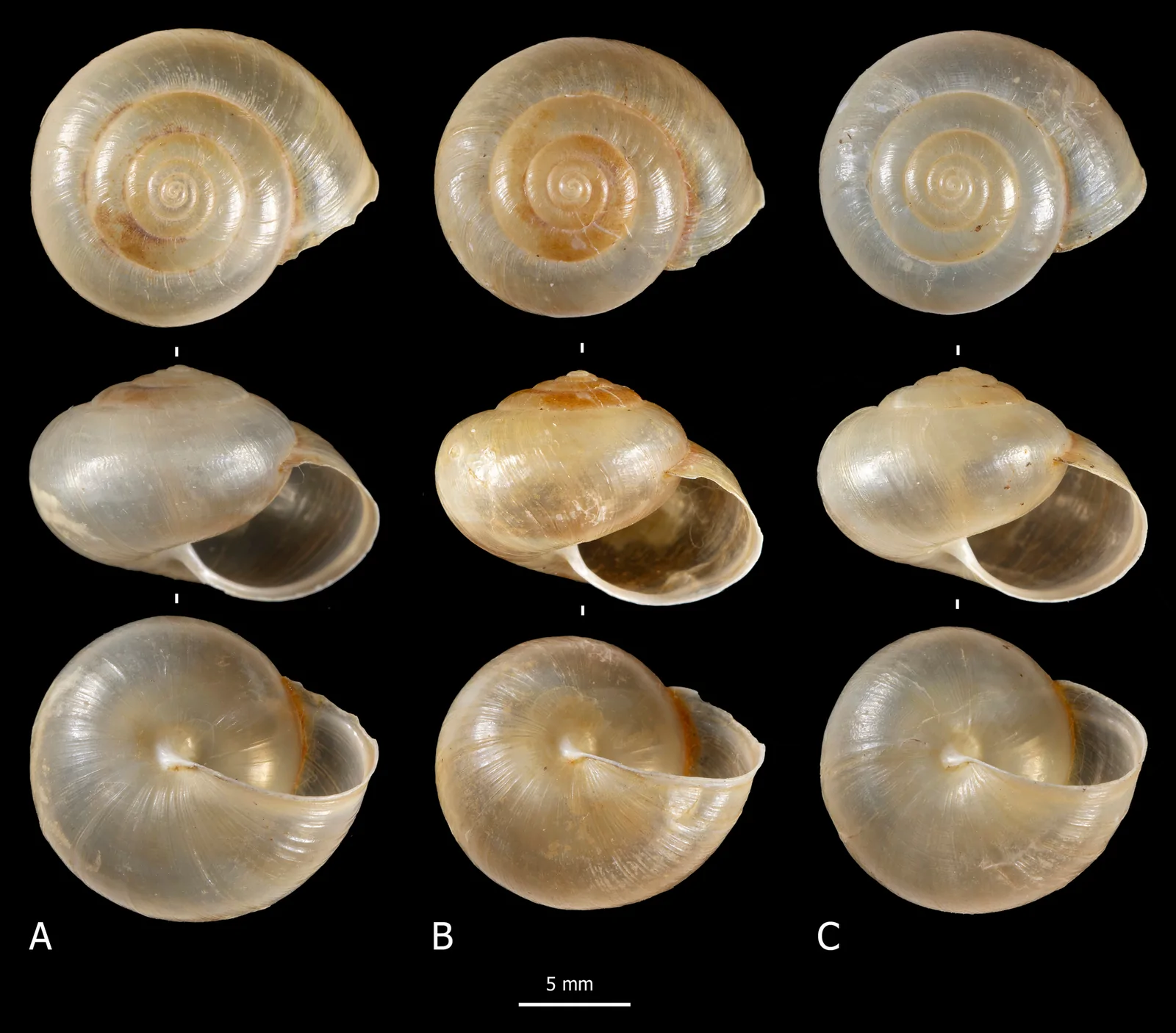
Shells of *Monacha cf. rizzae*. Specimens from Strada statale Fondo Valle del Noce, at km 23, A. Halgass leg. Oct. 2010 (FGC 42958) (**A–C**).

**Figure 19.**
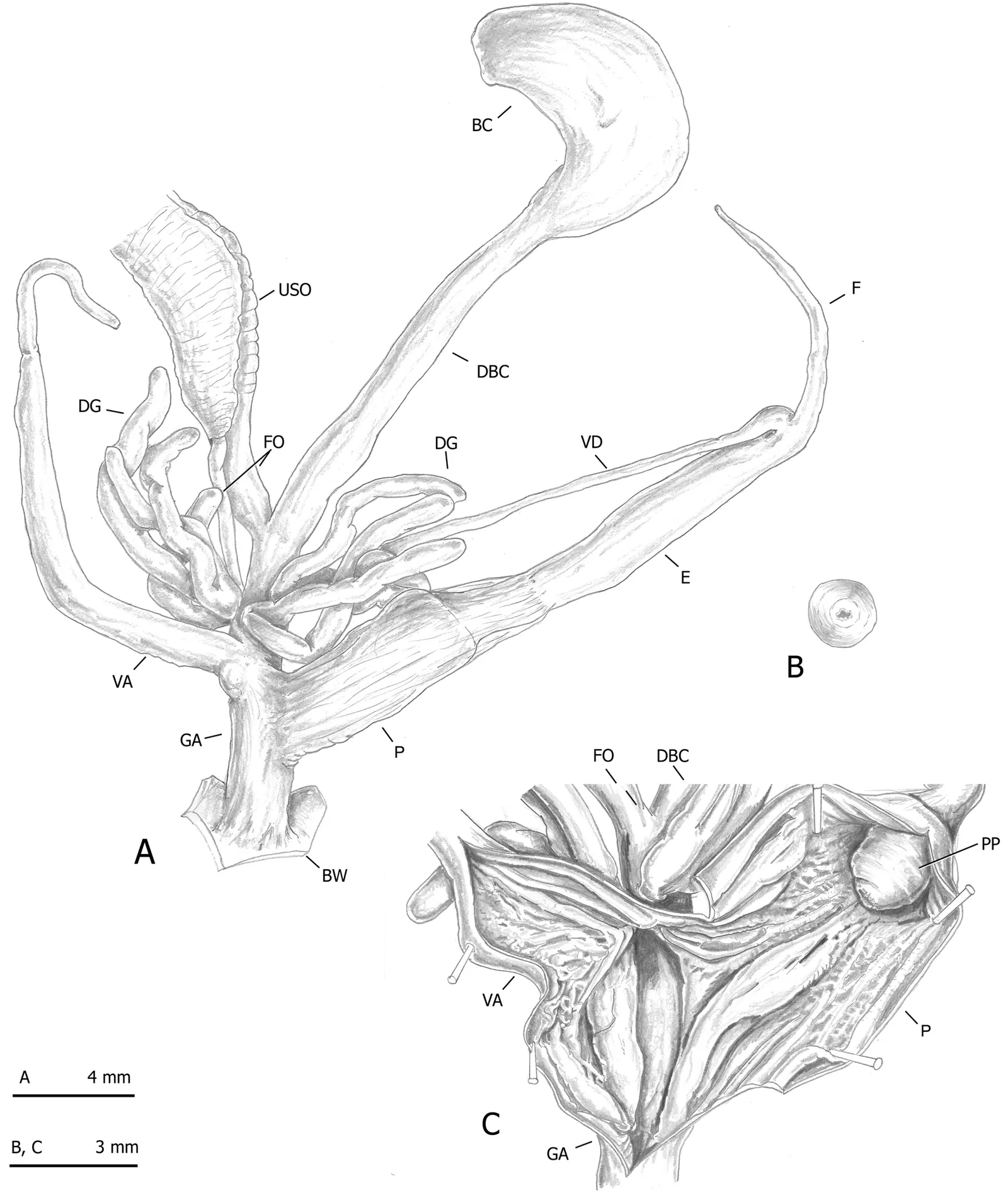
Genital anatomy of *Monacha cf. rizzae*. Specimens from Strada statale Fondo Valle del Noce, at km 23, A. Halgass leg. Oct. 2010 (FGC 42958) (**A, B**) and Strada statale di Sapri, at km 18, A. Halgass leg. Oct. 2010 (FGC 38945) (**C**). Distal genitalia (A), transverse section of apical penial papilla (B) and internal structure of vagina and penis (**C**).

**Figure 20.**
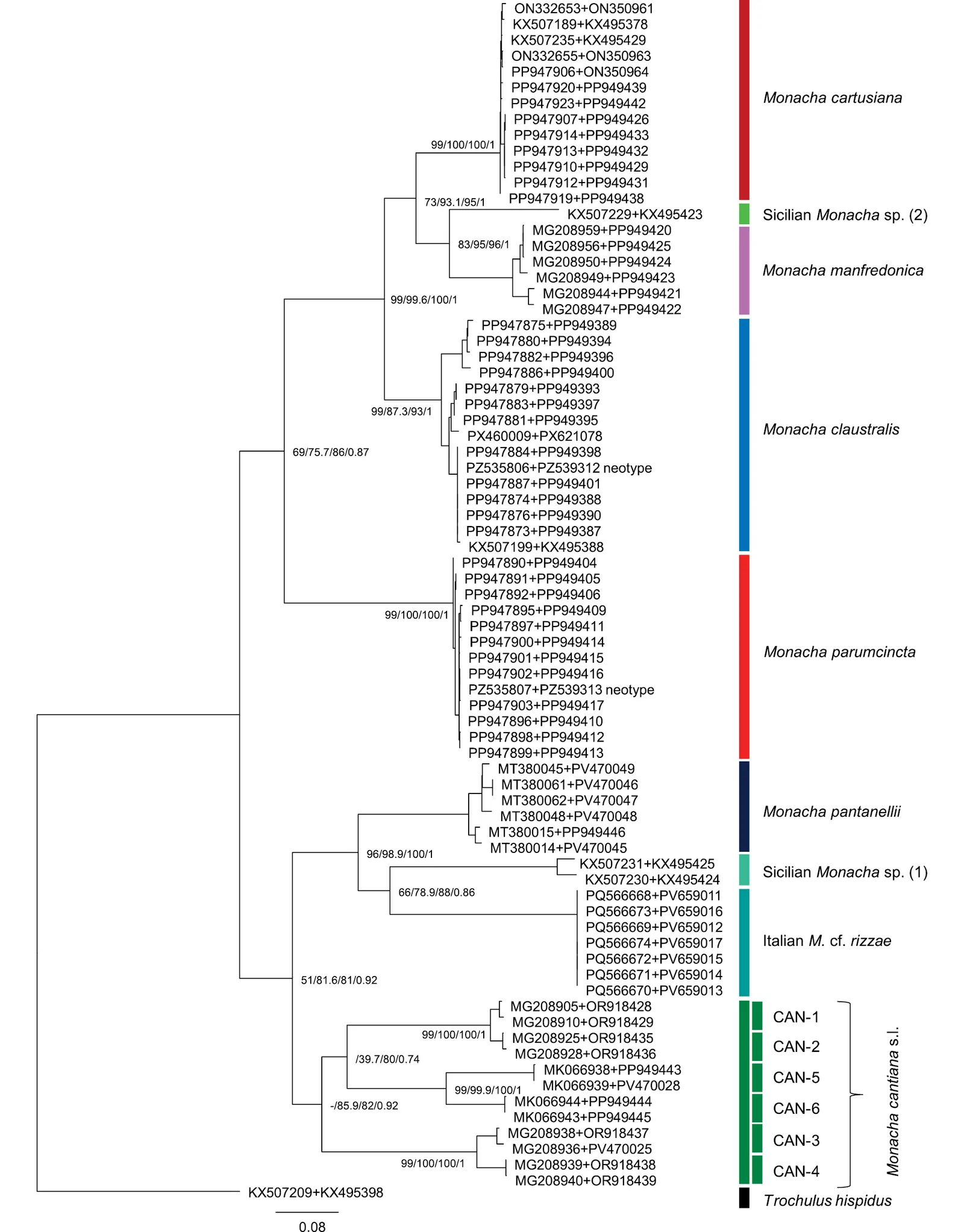
Maximum Likelihood (ML) tree of concatenated sequences of COI and 16SrDNA of *Monacha* species analysed in this paper. Concatenated COI and 16SrDNA sequences of Sicilian *Monacha* spp. 1 & 2 and Italian *M. cf. rizzae* were compared with COI+16SrDNA sequences of *M. claustralis*, *M. manfredonica*, *M. cartusiana*, *M. parumcincta*, *M. pantanellii* and *M. cantiana* s.l. obtained from GenBank (Table 1). Numbers next to main branches indicate (left to right): bootstrap supports above 50% calculated by ML-MEGA12 (Kumar et al. 2024) on 1000 replicates (Felsenstein 1985), SH-aLRT and ultrafast bootstrap in IQ-Tree (Nguyen et al. 2015; Hoang et al. 2018), and posterior probabilities by BI (Ronquist et al. 2012). The tree was rooted with *Trochulus hispidus* concatenated sequences obtained from GenBank (Table 1).

**Figure 21.**
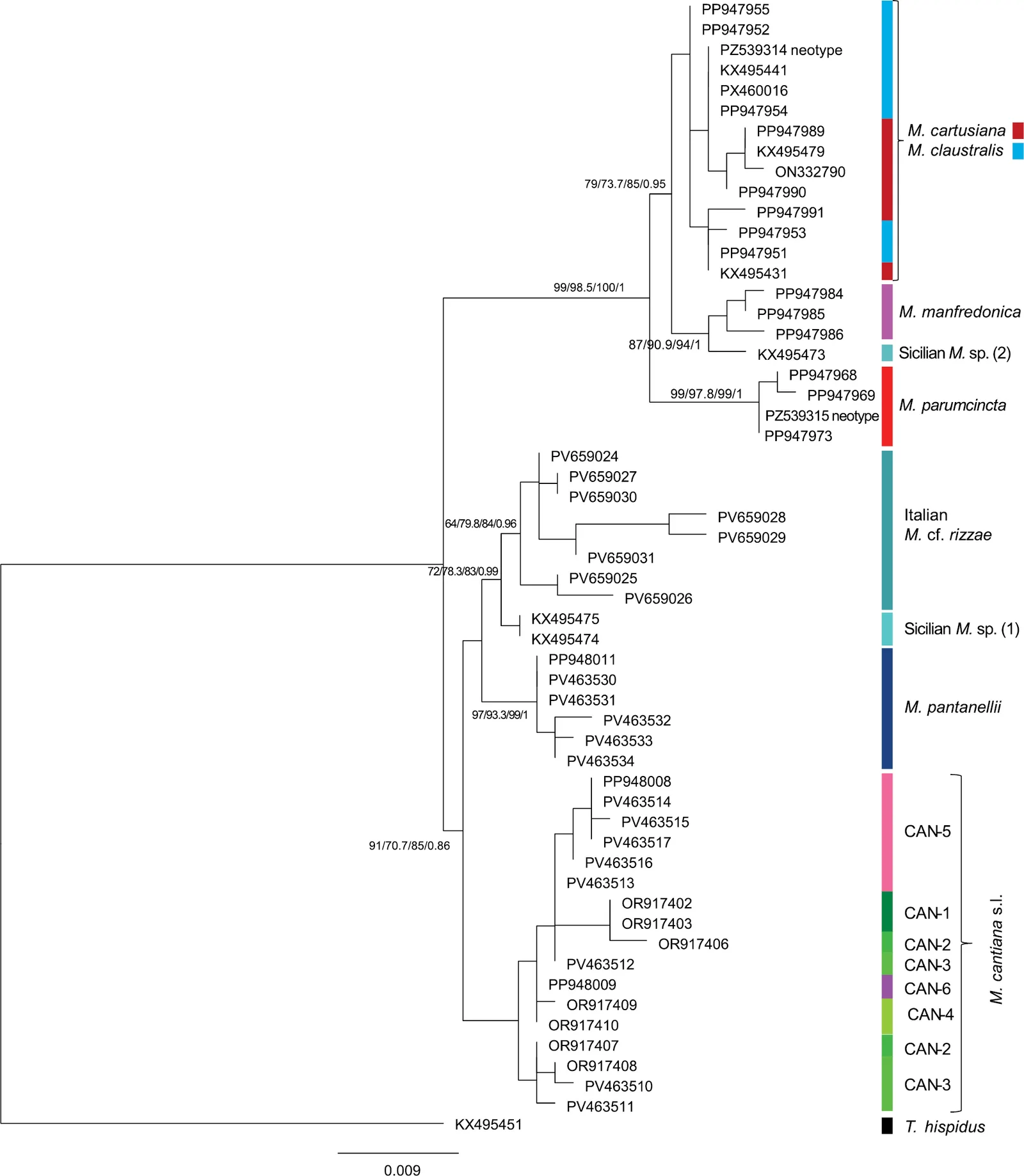
Maximum Likelihood (ML) tree of ITS2 (flanked with 5.8S and 28SrDNA) sequences of *Monacha* species analysed in this paper. ITS2 sequences of Sicilian *Monacha* spp. 1 & 2 and Italian *M. cf. rizzae* (Table 1) were compared with ITS2 sequences of *M. claustralis*, *M. cartusiana*, *M. manfredonica*, *M. parumcincta*, *M. pantanellii* and *M. cantiana* s.l. obtained from GenBank (Table 1). Numbers next to main branches indicate (left to right): bootstrap supports above 50% calculated by ML-MEGA12 (Kumar et al. 2024) on 1000 replicates (Felsenstein 1985), SH-aLRT and ultrafast bootstrap in IQ-Tree (Nguyen et al. 2015; Hoang et al. 2018), and posterior probabilities by BI (Ronquist et al. 2012). The tree was rooted with *Trochulus hispidus* sequences from GenBank (Table 1).

Figs 16, 17, 18, 19, 20, 21, 27

Helix bicincta Benoit, 1843 [25 July]: 255, plate 2, fig. 11; Type locality: Syracuse, Sicily. Type material: unknown. Junior homonym with Helix bicincta Menke, 1830 (currently Jeanneretia bicincta (Menke, 1830) from Cuba).
Helix rizzae Aradas, 1843 [9 September]: [1]; Type locality: Syracuse, Sicily. Type material: unknown.

##### Material examined.

Italy – **Sicily** • 2 spirit specimens; Siracusa province, Fontane Bianche; 33SWA19; 21 Oct. 1984; A. Giudice leg.; FGC 24485 • 1 shell; Siracusa; 33SWA20; 16 Mar. 1985; A. Giudice leg.; FGC 24534; • 2 shells; same locality; 16 May 1985; A. Giudice leg.; FGC 24481.

Material examined tentatively assigned to *M. rizzae*. Italy – **Sicily** • 1 spirit specimen; Palermo province, Pizzo Sant’Angelo; 33SVC10; 09 Oct. 1994; G. Manganelli leg.; FGC 6637 • 3 shells, 7 spirit specimens; Palermo province, Monte Pellegrino; 33SUC52; 14 Sept. 1984; F. Giusti & G. Manganelli leg.; FGC 24433.

Material examined from peninsular Italy, tentatively assigned to *M. rizzae*. Italy – **Liguria** • 3 specimens; Genova province, Paggi; 32TNQ21; 02 Nov. 2018; M. Bodon & G. Vezzani leg.; FGC 60087 – **Tuscany** • 3 specimens; Livorno province, near railway station of Livorno; 32TPP02; May 2008; F. Giusti leg.; FGC37037 • 28 specimens; Salviano (Livorno); 32TPP02; Oct. 2009; A. Margelli leg.; FGC 37852 – **Apulia** • 8 specimens; Lecce province, Serra di Caprarica del Capo; 34TBK72; 26 Oct. 1997; D. Ferreri leg.; FGC 35006 • 4 specimens; Lecce province, Valle dell’Idro; 34TBK84; 22 Sept. 1998; D. Ferreri leg.; FGC 35012 – **Basilicata** • 5 specimens; Potenza province, Strada statale di Sapri (S.S. n. 104), at km 18; 40°04'37.15"N, 15°43'26.66"E; Oct. 2010; A. Halgass leg.; FGC 38945 (population 1 used in molecular analysis, see Table 1) • 8 specimens; Potenza province, Strada statale Fondo Valle del Noce (S.S. n. 585), at km 23; 40°05'21.00"N, 15°43'53.67"E; Oct. 2010; A. Halgass leg.; FGC 42958 (population 2 used in molecular analysis, see Table 1) – **Calabria** • 5 specimens; Cosenza province, 500 m N of Laino Castello; 39°56'36.61"N, 15°58'41.15"E; 04 Oct. 2020; L. Favilli leg.; FGC 50338.

##### Morphological description.

Shell (Fig. 16A–C) dextral, small to medium in size, subglobose to globose, rather robust, opaque, horn-yellow to brownish in colour with two whitish spiral bands – one subsutural, one peripheral – and white and reddish collabral band near aperture. Whorls 5–5^1^/_2_, slightly convex and separated by superficial sutures; last whorl ~ 8/10 of shell height, not dilated and descending slightly near aperture. Aperture rather large, round to oval (AW/AH ~ 1.5) and slightly prosocline. Peristome interrupted, slightly reflected on columella, thickened and with variably evident whitish callous rim on internal outer margin. Umbilicus closed by reflected columellar peristome. Protoconch and teleoconch smooth, with very faint scattered collabral growth lines. Shell dimensions: H – 9.2–11.8 mm; D – 13.0–14.4 mm (*n* = 5).

Female distal genitalia (Fig. 17A, C) include free oviduct, bursa copulatrix and its duct, digitiform glands, vagina, and vaginal appendix. Free oviduct short and variably wide. Bursa copulatrix rather large, triangular, shoe- or bean-like, with long duct (usually much longer than vagina). Vagina very short, without refringent muscular ring or lateral sac between digitiform glands and vaginal appendix, and with series of narrow irregular internal pleats. Digitiform glands disposed on opposite sides of vagina in two groups of two tufts, each with one or two branches. Vaginal appendix very long, inserted near distal end of vagina, without dilated basal portion but tapering progressively towards tip.

Male distal genitalia (Fig. 17A–C) include vas deferens, flagellum, epiphallus and penis. Vas deferens very long and very slender. Flagellum short to rather long, slender, and pointed at tip. Epiphallus rather long and wide. Penis short and wide, enveloped by thin sheath. Penial papilla cylindrical, dilated at tip, with apical opening, usually rather thick external walls and internal lumen almost completely occupied by large central duct, round in section.

Genital atrium (Fig. 17A, C) rather short and wide, internally smooth or with variably developed, slightly defined pleats.

##### Molecular description.

No data available, but see Remarks.

##### Remarks.

This species was described as *Helix rizzae* by Aradas (1843: [1]) on a shell collected near Syracuse received from Alessandro Rizza and as *Helix bicincta* by Benoit (1843: 255) on material from Syracuse. Much of the early literature on this species concerns the dispute between Benoit and Aradas on the priority of the discovery (Germain 1921). Both nominal species were described in local journals: *Helix rizzae* in issue 143 of L’Occhio – Giornale di Scienze Arti Commercio published in Palermo on 9 September, *Helix bicincta* in the issues XI and XVI of volume 1 of Farfalletta, Opera Periodica Scientifica Letteraria Artistica published in Messina on 10 May (issue XI) and 25 July (issue XVI). Thus Benoit’s name was published first, but being a junior primary homonym of *Helix bicincta* Menke, 1830 (now *Jeanneretia bicincta*, Cepolidae Ihering, 1909 according to MolluscaBase eds 2025), the valid name for this species is the one proposed by Aradas.

*Helix rizzae* (often reported as “*Helix bicincta*”) has long been regarded as a variety of *Helix olivieri* (e.g. Tryon 1887: 192; Westerlund 1889: 86; Germain 1921: 199–201). Hesse (1931) re-evaluated it (as “*Theba bicinta*”) based on anatomical study of three specimens from Tropea received from F. Settepassi, only one of which was an adult (cf. Hesse 1931: 38–39, pl. 5, fig. 39a, b), that he regarded as belonging to a species different from those assigned to “*Theba olivieri*” (cf. Hesse 1931: pl. 5, fig. 43a, b). Forcart (1965) confirmed its validity, arguing that *Monacha rizzae* was very similar to *Monacha parumcincta*, the difference residing in the position of the distal female genital appendix: atrial in the former, vaginal in the latter. Actually, the vaginal appendix in the specimen depicted by Hesse (1931) is also vaginal not atrial and it is also very likely that the specimen from Tropea examined by Hesse (1931) did not belong to *Monacha rizzae* but to *Monacha manfredonica* due to the short vaginal appendix.

Reitano et al. (2009) first published a detailed study on the genital anatomy of specimens collected around Syracuse (the type locality) (another previous anatomical report on topotypical specimens by Pintér and Szigethy (1976) was unfortunately very poor). The anatomical report by Reitano et al. (2009) is consistent with our investigation on specimens from Syracuse having a shell corresponding to the one illustrated. Based on the study of topotypical specimens, *Monacha rizzae* has a globose imperforate shell (Fig. 16A, B) and *cantiana*-like anatomy (Fig. 17A, B). A *Monacha* species with these shell and anatomical features has been reported from western Sicily (Figs 16C, 17C), Calabria, Basilicata (Figs 18, 19), Apulia (see also Ferreri et al. 2005: 106, 108–109, figs 45–48), and central Italy, where it was introduced near Leghorn (Tuscany) and Genoa (Liguria).

Unfortunately no molecular data is available for topoptypical specimens from Syracuse. Molecular data for Sicilian specimens which could belong to this group was reported by Neiber and Hausdorf (2017) (ZMH 92197, ZMH 92199 – both collected near Palermo). Molecular data is instead available for two Italian populations from Basilicata (“Strada statale di Sapri, at km 18” and “Strada statale Fondo Valle del Noce, at km 23”; Figs 20, 21).

The sequences COI+16SrDNA (KX507230+KX495424, KX507231+KX495425) and ITS2 (flanked by fragments of 5.8S and 28SrDNA) (KX495474, KX495475), deposited by Neiber and Hausdorf (2017) for *Monacha* sp. 1 probably represent the western Sicilian *Monacha cf. rizzae* group in clades separate from our Italian *M. cf. rizzae* (Figs 20, 21). This separateness is also shown by K2P distances (Table 4) which lead us to consider them to probably be different species. In our previous papers (Pieńkowska et al. 2018b, 2020, 2022) we emphasised the need for caution in the use of K2P genetic distances for inferring species distinctness in stylommatophoran gastropods. Consequently, the effective conspecificity of the Sicilian populations and those from southern Italy is a hypothesis to be verified. Given this uncertainty, the redescription of *Monacha rizzae* is based only on the topotypical specimens from Syracuse.

**Table 4.** Ranges of K2P genetic distances between the COI sequences analysed (mean value in brackets).

**Comparison**	**COI (%)**
Within *Monacha manfredonica* (MG2089xx)	1.5–4.1 (3.2)
Within Sicilian *Monacha* sp. 1 (KX507230–KX507231)	3.3 (3.3)
Within Italian *Monacha cf. rizzae* (PQ566664–PQ566667)	0.0–0.3 (0.2)
Within Sicilian *Monacha* sp. 2 (KX507229)	0 (0)
Within Fiume Alli *Monacha* sp. (PX651375–PX651376)	5.2 (5.2)
Between *M. manfredonica* and Sicilian *Monacha* sp. 1	18.9–19.2 (19.1)
Between *M. manfredonica* and Italian *Monacha cf. rizzae*	20.5–22.1 (21.3)
Between *M. manfredonica* and Sicilian *Monacha* sp. 2	14.1–14.6 (14.3)
Between *M. manfredonica* and Fiume Alli *Monacha* sp.	18.2–19.6 (18.6)
Between Sicilian *Monacha* sp. 1 and Italian *Monacha cf. rizzae*	19.0–20.1 (19.5)
Between Sicilian *Monacha* sp. 1 and *Monacha* sp. 2	19.6–20.4 (20.0)
Between Sicilian *Monacha* sp. 1 and Fiume Alli *Monacha* sp. 3	18.2–19.4 (18.9)
Between Sicilian *Monacha* sp. 1 and *Monacha cartusiana*	20.1–21.7 (21.0)
Between Sicilian *Monacha* sp. 1 and *Monacha claustralis*	15.5–17.4 (16.5)
Between Sicilian *Monacha* sp. 1 and *Monacha pantanellii*	17.0–18.4 (17.6)
Between Sicilian *Monacha* sp. 1 and *Monacha parumcincta*	18.8–19.9 (19.3)
Between Italian *Monacha cf. rizzae* and Sicilian *Monacha* sp. 2	23.2 (23.2)
Between Italian *Monacha cf. rizzae* and Fiume Alli *Monacha* sp.	17.4–18.2 (17.8)
Between Italian *Monacha cf. rizzae* and *Monacha cartusiana*	21.2–21.9 (21.6)
Between Italian *Monacha cf. rizzae* and *Monacha claustralis*	19.8–21.5 (20.7)
Between Italian *Monacha cf. rizzae* and *Monacha pantanellii*	17.5–18.4 (17.9)
Between Italian *Monacha cf. rizzae* and *Monacha parumcincta*	20.2–21.6 (20.9)
Between Sicilian *Monacha* sp. 2 and Fiume Alli *Monacha* sp. 3	16.5 (16.5)
Between Sicilian *Monacha* sp. 2 and *Monacha cartusiana*	13.8–14.0 (14.0)
Between Sicilian *Monacha* sp. 2 and *Monacha claustralis*	15.5–16.0 (15.8)
Between Sicilian *Monacha* sp. 2 and *Monacha pantanellii*	17.7–18.0 (17.9)
Between Sicilian *Monacha* sp. 2 and *Monacha parumcincta*	16.7–16.9 (16.8)
Between Fiume Alli *Monacha* sp. and *Monacha cartusiana*	17.8–18.8 (18.3)
Between Fiume Alli *Monacha* sp. and *Monacha claustralis*	16.5–17.8 (17.0)
Between Fiume Alli *Monacha* sp. and *Monacha pantanellii*	13.6–16.8 (15.1)
Between Fiume Alli *Monacha* sp. and *Monacha parumcincta*	10.8–16.4 (13.6)

For other K2P distances see our previous papers Pieńkowska et al. (2018b, 2020) and Manganelli et al. (2025).

**Figure 22.**
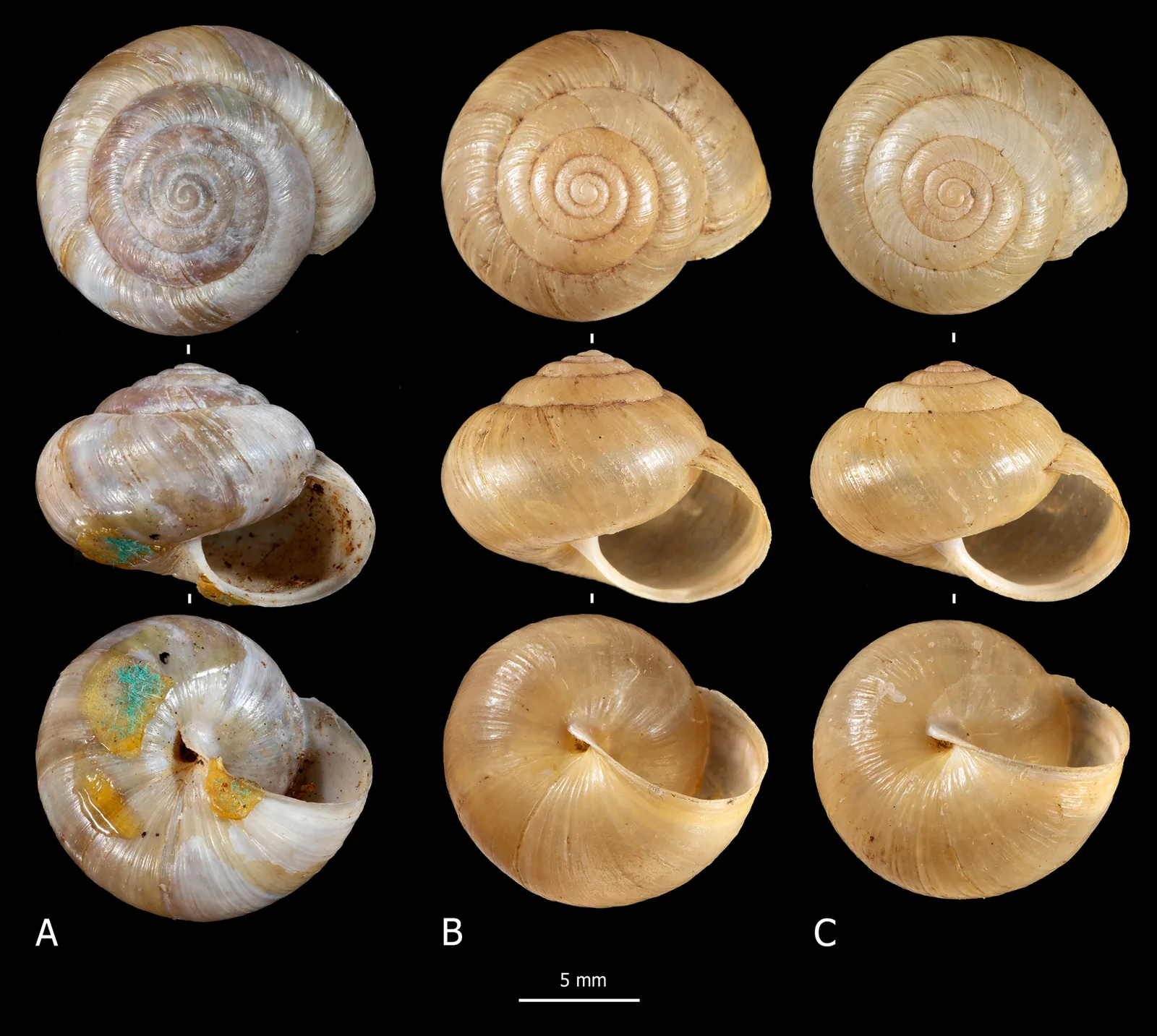
Shells of *Monacha cf. consona*. Syntype of *Helix consona* figured in Rossmässler’s Iconographie as fig. 572 (SMF 6642/1) (**A**) and two specimens from Monte Soro, F. Giusti & G. Manganelli leg. 10 Oct. 1994 (FGC 6640) (**B, C**). Photo credits: Sigrid Hof (**A**).

**Figure 23.**
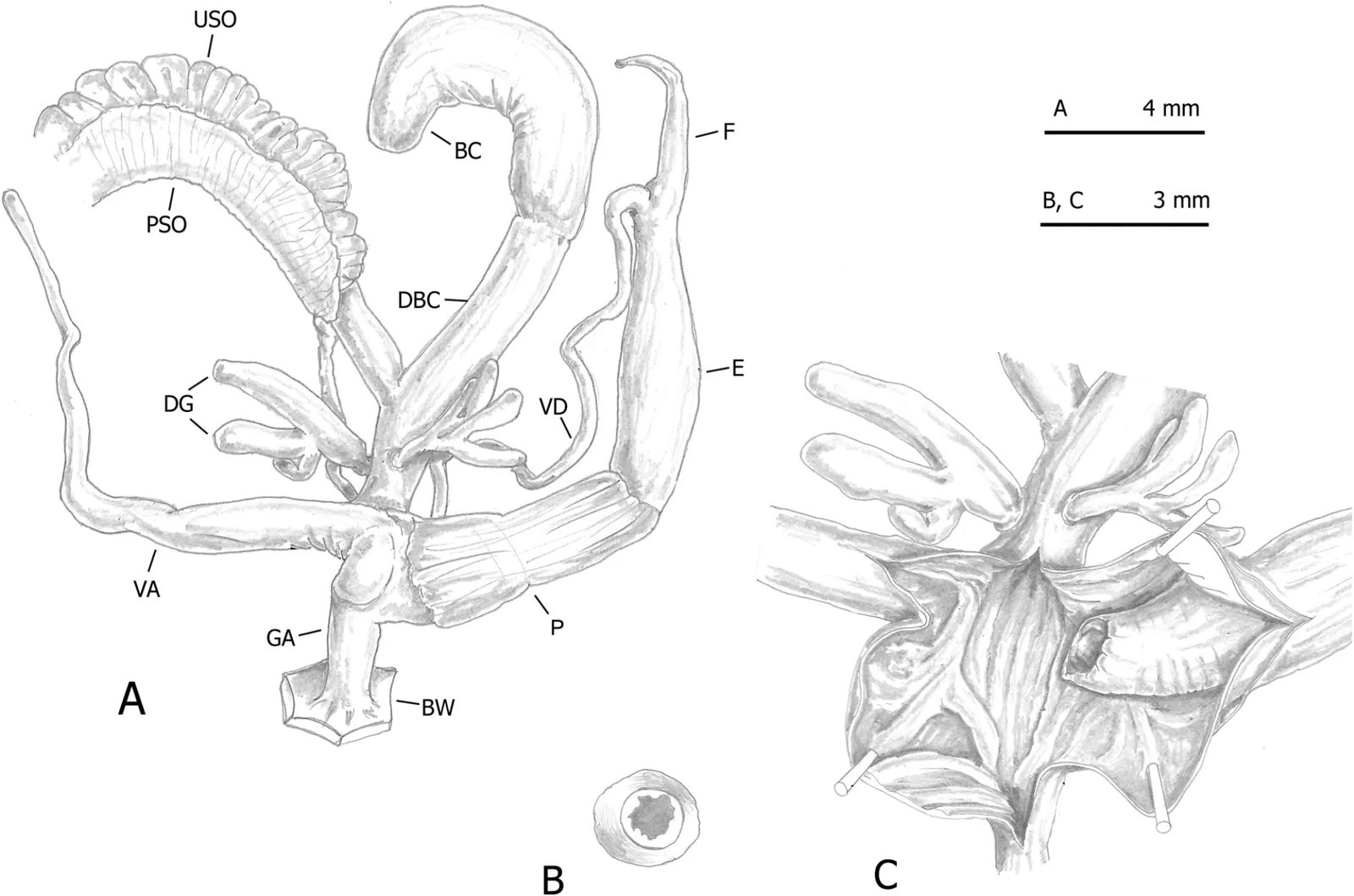
Genital anatomy of *Monacha cf. consona*. Specimen from Monte Soro, F. Giusti & G. Manganelli leg. 10 Oct. 1994 (FGC 6640). Distal genitalia (**A**), transverse section of apical penial papilla (**B**) and internal structure of vagina and penis (**C**).

Finally, there is another putative Sicilian *Monacha* species (Figs 22A–C, 23A–C), different from all the other Sicilian species, characterised by an umbilicated shell: *Helix consona* Rossmässler, 1839 (8, pl. 43, figs 572, 573). It was interpreted as a Sicilian species by most early authors (e.g. Philippi 1844; Pfeiffer 1848b, 1852; Tryon 1887) and according to Benoit (1859, 1881) occurred in the neighbourhood of Palermo. However, it was also reported from southern Italy (e.g. Paulucci 1880; Westerlund 1889; Bellini 1900, 1915; Degner 1927; Forcart 1965), Dalmatia (Walderdorff 1864), Greece (Bourguignat 1853; Westerlund 1889) and even western Turkey (Bourguignat 1855). Degner (1927: 58–61, figs 7, 8) examined specimens from four localities in Campania and Basilicata, providing an anatomical description based on specimens collected at Sicignano (Salerno). Forcart (1965) listed it as a valid species, intended to restrict the type locality to the environs of Palermo (“Umgebung von Palermo”) and reported it from several sites in southern Italy (Apulia, Basilicata, and Calabria). However, neither Degner (1927) nor Forcart (1965) clarified how this species could be distinguished from the others from southern Italy and Sicily and from the widespread *Monacha cantiana*. It is therefore not by chance that Giusti (1973) considered it a junior synonym of *Monacha gregaria*, the name under which he grouped all the Sicilian *Monacha*, while Manganelli et al. (1995) considered it a species in need of revision.

The type material consists of a syntype kept in Rossmässler’s collection at Senckenberg Forschungsinstitut, Frankfurt am Main (SMF 6642/1) (Fig. 22A) corresponding to the shell illustrated in fig. 572 according to Zilch’s handwritten label. Unfortunately, it does not allow the actual identity of the species to be established. It is reported from the neighbourhood of Palermo in the past (Benoit 1859, 1881) and recently (Reitano et al. 2012) but the only materials at our disposal that match the shell features of this species are from north-eastern Sicily (Nebrodi Mountains) and show *cantiana*-like anatomy (Fig. 23). The relationships between this form, the true *Monacha cantiana*, other species with imperforate shell and *cantiana*-like anatomy and the possible similar-shelled species reported from western Sicily remain unknown.

#### Monacha

Taxon classificationAnimaliaStylommatophoraHygromiidae

sp. 3

F60E3060-0FA1-5464-87D9-0BE6697E6D41

Figs 24, 25, 26, 27

##### Material examined.

Italy – **Calabria** • 3 shells, 5 spirit specimens; Reggio Calabria province, Stilo; 33SXC25; 17 Sept. 1984; L. Castagnolo, F. Giusti & G. Manganelli leg.; FGC 24455 • 1 shell, 13 spirit specimens; Vibo Valentia province, near Lago Angitola; 33SXC08; 15 Jul. 1987; N. Pirozzi leg.; FGC 24529 • 1 spirit specimen; Vibo Valentia province, Pizzo Calabro; 33SXC08; 18 Sept. 1981; F. Giusti leg.; FGC 24487 • 6 spirit specimens; Vibo Valentia province, Briatico; 38°43'34.00"N, 16°01'19.38"E; 17 Oct. 2024; W. Renda leg.; FGC 58475 • 10 spirit specimens; Vibo Valentia province, Pizzo Calabro; 38°45'47.30"N, 16°11'45.20"E; 12 May 2007; W. Renda leg.; FGC 36559 • 8 spirit specimens; Vibo Valentia province, Certosa di Serra San Bruno; 38°34'01.16"N, 16°19'15.32"E; 10 Oct. 2024; W. Renda leg.; FGC 58681• 10 spirit specimens; Catanzaro province, Fiume Alli, downstream confluence with Fosso Sarbato; 38°57'55.57"N, 16°36'43.71"E; 20 Oct. 2007; A. Hallgass leg.; FGC 36609 (population used in molecular analysis, see Table 1).

##### Morphological description.

**Figure 24.**
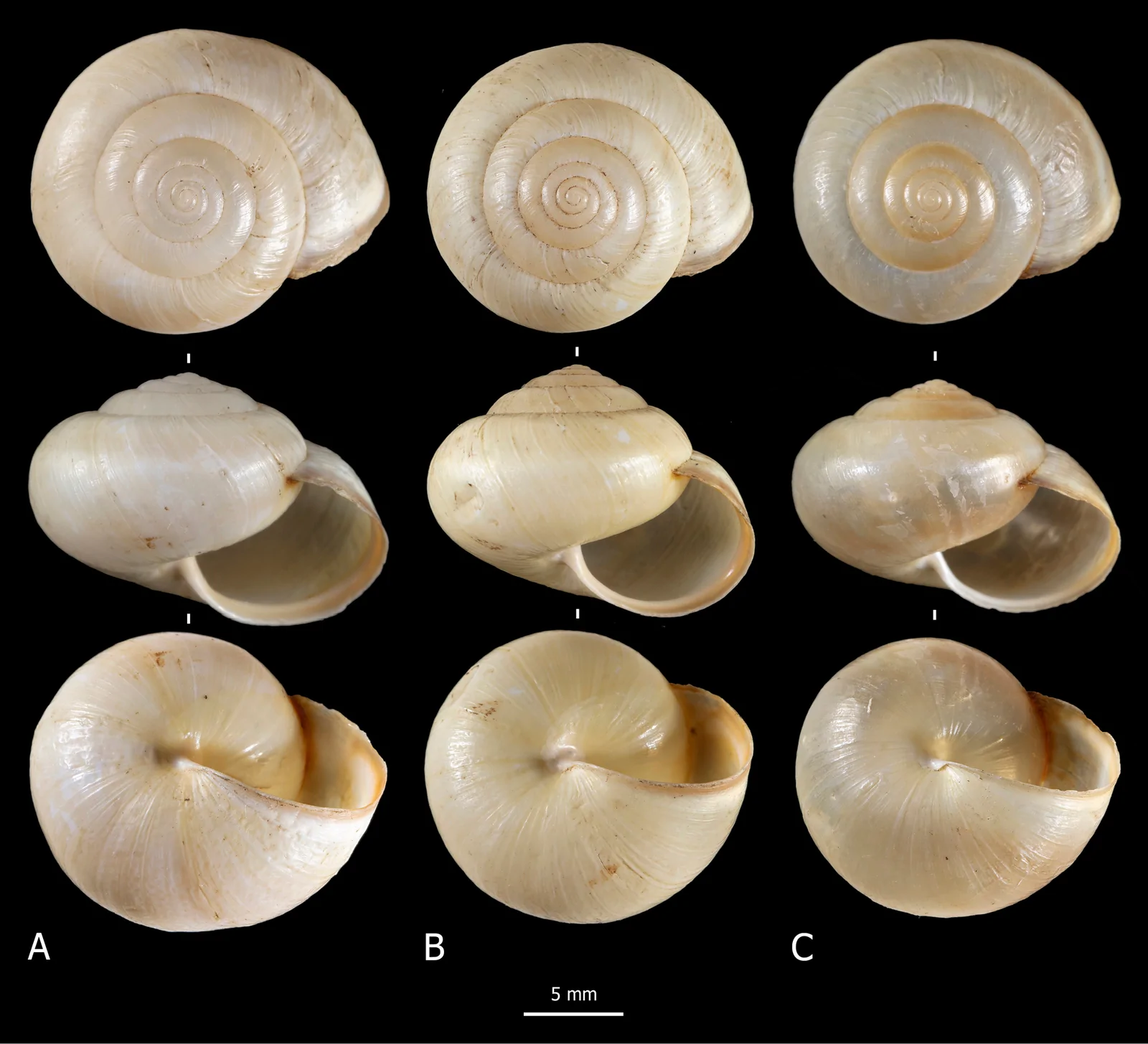
Shells of *Monacha* sp. 3. Specimens from Fiume Alli, downstream confluence with Fosso Sarbato, A. Hallgass leg. 20 Oct. 2007 (FGC 36609) (**A–C**).

Shell (Fig. 24A–C) dextral, medium in size, subglobose to globose, rather robust, opaque, horn-yellow to brownish in colour with two whitish spiral bands – the subsutural one larger, the peripheral one smaller – and white and reddish collabral band near aperture. Whorls 5–5^1^/_2_, slightly convex and separated by superficial sutures; last whorl ~ 8–9/10 of shell height, not dilated and descending slightly near aperture. Aperture rather large, round to oval (AW/AH ~ 1.3–1.5) and slightly prosocline. Peristome interrupted, slightly reflected on columella, thickened and with variably evident whitish callous rim on internal outer margin. Umbilicus closed by reflected columellar peristome. Protoconch and teleoconch smooth, with very faint scattered collabral growth lines. Shell dimensions: H – 12.8 ± 0.8 mm; D – 17.3 ± 0.9 mm (*n* = 15).

**Figure 25.**
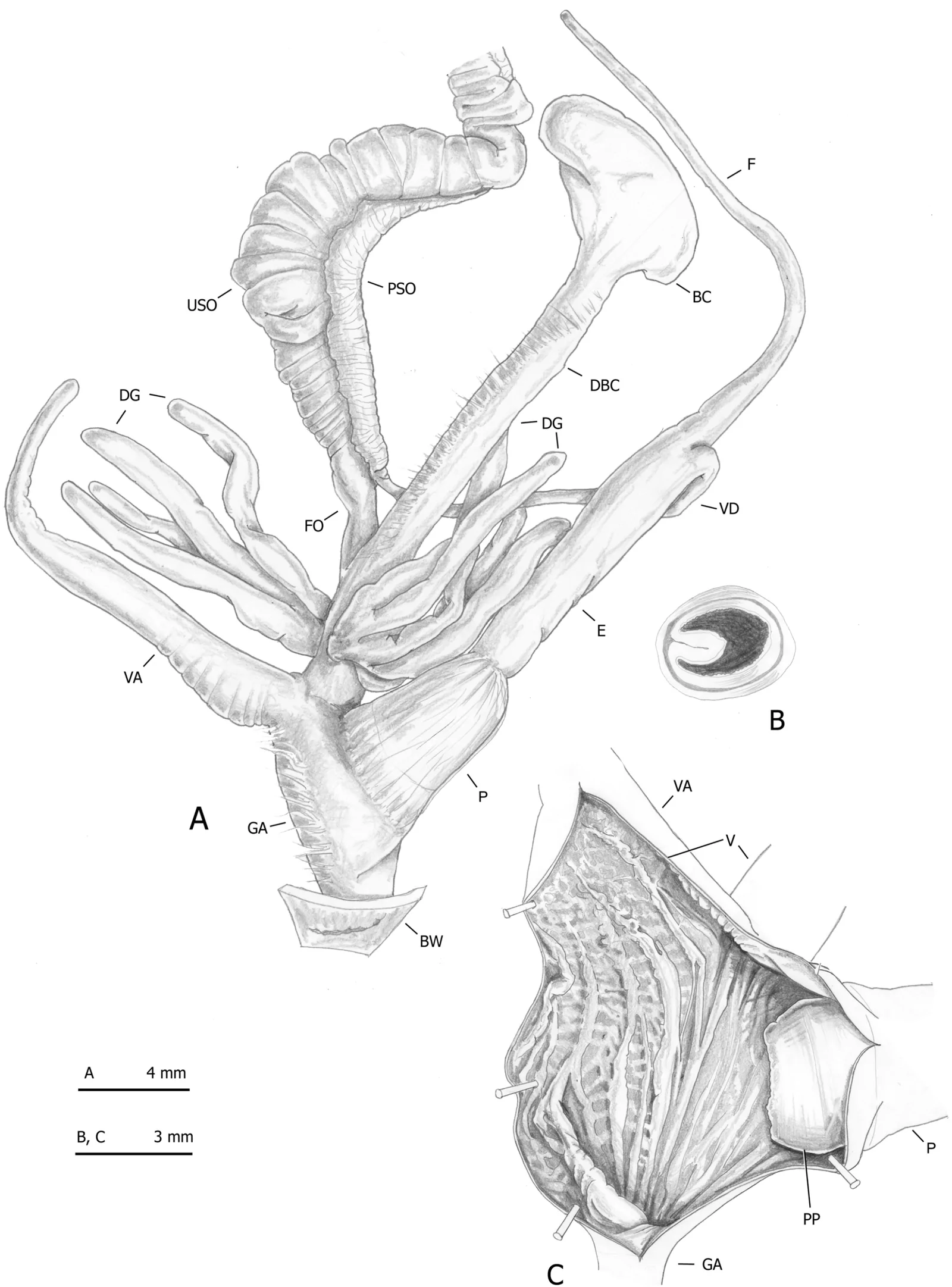
Genital anatomy of *Monacha* sp. 3. Specimens from Fiume Alli, downstream confluence with Fosso Sarbato, A. Hallgass leg. 20 Oct. 2007 (FGC 36609). Distal genitalia (**A**), transverse section of apical penial papilla (**B**) and internal structure of vagina and penis (**C**).

**Figure 26.**
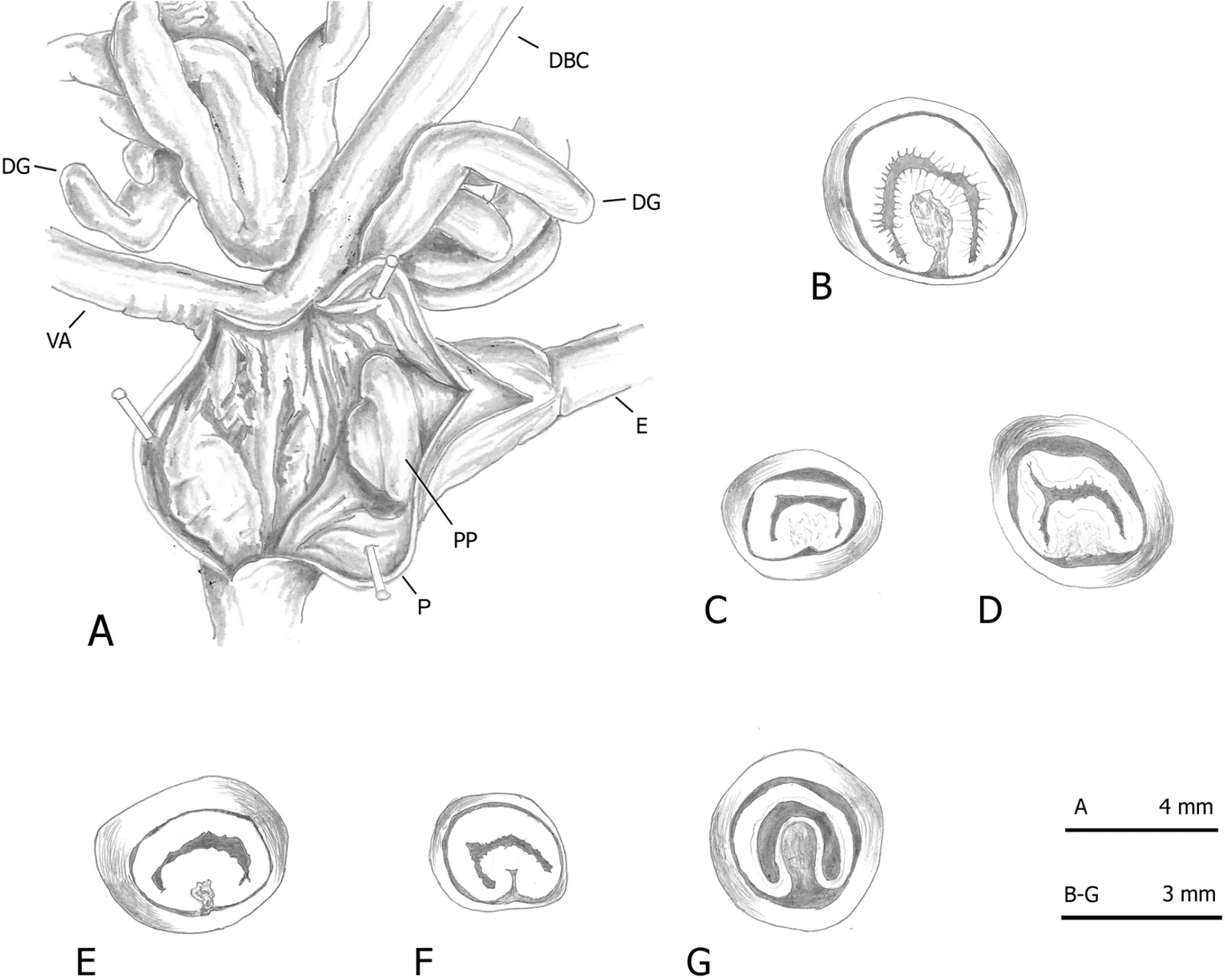
Genital anatomy of *Monacha* sp. 3. Specimens from Pizzo Calabro, F. Giusti leg. 18 Sept. 1981 (FGC 24487) (**A, B**), Certosa di Serra San Bruno, W. Renda leg. 10 Oct. 2024 (FGC 58681) **C, D**, Stilo, L. Castagnolo, F. Giusti & G. Manganelli leg. 17 Sept. 1984 (FGC 24455) (**E**), near Lago Angitola, N. Pirozzi leg. 15 Jul. 1987 (FGC 24529) (**F**), Briatico, W. Renda leg. 17 Oct. 2024 (FGC 58475) (**G**). Internal structure of vagina and penis (**A**) and sections of the penial papilla (**B–G**).

Female distal genitalia (Figs 25A, 25C, 26A) include free oviduct, bursa copulatrix and its duct, digitiform glands, vagina, and vaginal appendix. Free oviduct short and variably wide. Bursa copulatrix rather large, triangular, shoe- or bean-like, with long duct (usually much longer than vagina). Vagina very short, without refringent muscular ring or lateral sac between digitiform glands and vaginal appendix, but with series of narrow irregular internal pleats, one of which more robust or knob-shaped distally. Digitiform glands disposed on opposite sides of vagina in two groups of two tufts, each with one or two branches. Vaginal appendix very long, inserted near distal end of vagina, without dilated basal portion, but tapering progressively towards tip.

Male distal genitalia (Figs 25A–C, 26A–G) include vas deferens, flagellum, epiphallus and penis. Vas deferens very long and very slender. Flagellum short to rather long, slender, and pointed at tip. Epiphallus rather long and wide. Penis very short, cone-shaped, and wide, enveloped by thin sheath. Penial papilla cylindrical, conical at tip, with apical opening, usually rather thick external walls and internal lumen almost completely occupied by large central duct, C-shaped or roughly C-shaped in apical section.

Genital atrium (Figs 25A, 25C, 26A) rather short and wide, internally smooth or with variably developed, vaguely defined pleats.

##### Molecular description.

**Figure 27.**
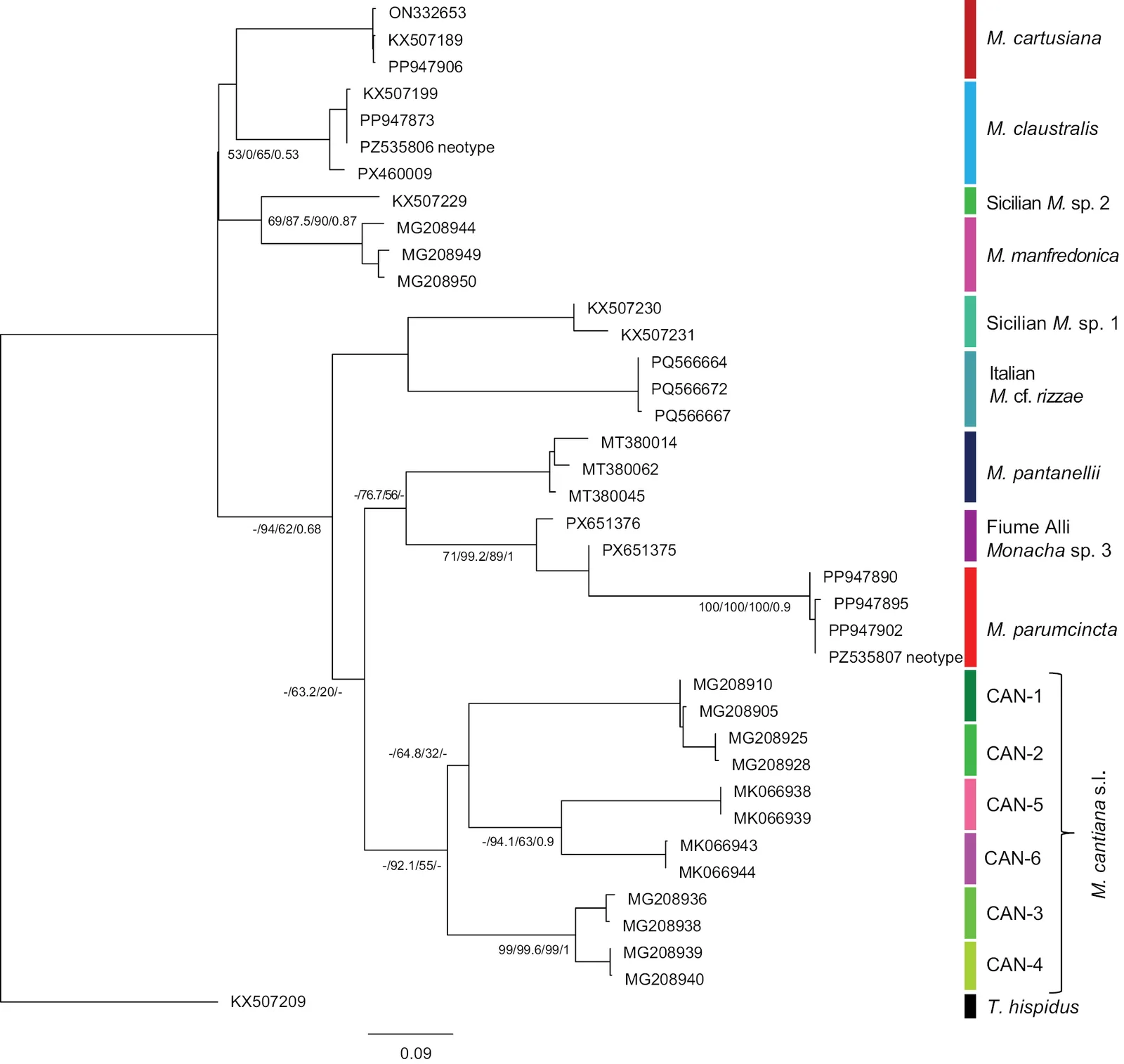
Maximum Likelihood (ML) tree of COI sequences of all *Monacha* species analysed in this paper. COI sequences of *Monacha* sp. 3 from Fiume Alli were compared with COI sequences of *M. claustralis*, *M. cartusiana*, *M. parumcincta*, *M. manfredonica*, *M. pantanellii*, Sicilian *Monacha* spp. 1 & 2, Italian *M. cf. rizzae* and *M. cantiana* s.l. obtained from GenBank (Table 1). Numbers next to main branches indicate (left to right): bootstrap supports above 50% calculated by ML-MEGA12 (Kumar et al. 2024) on 1000 replicates (Felsenstein 1985), SH-aLRT and ultrafast bootstrap in IQ-Tree (Nguyen et al. 2015; Hoang et al. 2018), and posterior probabilities by BI (Ronquist et al. 2012). The tree was rooted with *Trochulus hispidus* concatenated sequences obtained from GenBank (Table 1).

Two sequences of COI gene were obtained from specimens collected in Fiume Alli. They were deposited in GenBank with accession numbers PX651375, PX651376 (Table 1) and compared with selected COI sequences of all other *Monacha* species analysed in this paper (Fig. 27). They appeared separately in the phylogenetic tree obtained and did not group with any COI sequences for other *Monacha* species.

##### Remarks.

We have listed separately some populations of a large *Monacha* from the extreme tip of peninsular Italy (central and southern Calabria). These populations are characterised by an imperforate shell and *cantiana*-like anatomy but with short cone-shaped penis and C-like or roughly C-like transverse section of the apical penial papilla. They could form another species but the available evidence is not definitive as only partial molecular examination has been performed.

The penial papilla of these specimens sometimes has a sort of apical ring (Figs 25C, 26A). Such a ring has also been reported in specimens assigned to *Monacha rizzae* / *Monacha cf. rizzae* (Ferreri et al. 2005: figs 42, 43; Reitano et al. 2009: fig. 11e). Sometimes considered distinctive of *Monacha rizzae*, this feature could be due to a fixation artifact. As in the case of the transverse section of the penial papilla, prudence is therefore required in evaluating anatomical features which may differ due to different degrees of sexual maturity or fixation artifacts. Molecular analysis indicates that *Monacha* sp. 3 from Fiume Alli bears a closer relationship to *M. parumcincta* from Corfu, and populations representing *M. pantanellii*, Italian *M. cf. rizzae*, and Sicilian *Monacha* sp. 1. The separation of Calabrian *Monacha* sp. from all other species of *Monacha* studied in this paper is also reflected by the values of the K2P genetic distances (Table 4). However, populations of *Monacha* sp. from central and southern Calabria definitely require further anatomical and molecular study to determine the species they belong to.

## Supplementary Material

XML Treatment for Monacha cartusiana

XML Treatment for Monacha manfredonica

XML Treatment for Monacha gregaria

XML Treatment for Monacha rizzae

XML Treatment for Monacha

## Additional information

### Artificial Intelligence (AI) use

The authors accept full responsibility for the content of the manuscript, including the disclosure of any use of AI.

No AI tools were used in the preparation of this manuscript.

### Author ORCIDs

G. Manganelli https://orcid.org/0000-0002-8453-280X

J.R. Pieńkowska https://orcid.org/0000-0003-0372-121X

D. Barbato https://orcid.org/0000-0003-1105-1711

A. Benocci https://orcid.org/0000-0003-3145-215X

K. Sosnowska-Kałasz https://orcid.org/0000-0002-7506-4231

A. Lesicki https://orcid.org/0000-0002-1924-1934

F. Giusti https://orcid.org/0000-0001-8722-4653

### Data availability

All of the data that support the findings of this study are available in the main text.
